# Hepatic Steatosis Severity Prediction in Nonobese Individuals: Machine Learning Model Development and Validation

**DOI:** 10.2196/82529

**Published:** 2026-06-19

**Authors:** Yitong Zhu, Yongshuai Wang, Shenyu Zhang, Jian Yang, Feng Zhang, Yan Liu, Jun Shang, Yongliang Zhang, Jizhou Wang, Lianxin Liu

**Affiliations:** 1 Department of Hepatobiliary Surgery, Centre for Leading Medicine and Advanced Technologies of IHM, The First Affiliated Hospital of USTC, Division of Life Sciences and Medicine, University of Science and Technology of China, No.96 Jinzhai Road, Hefei, Anhui, 230001, China, 86 13845159888; 2 Anhui Province Key Laboratory of Hepatopancreatobiliary Surgery, Hefei, Anhui, China; 3 Anhui Provincial Clinical Research Center for Hepatobiliary Diseases, Hefei, Anhui, China; 4 Department of Health Management Center, The First Affiliated Hospital of USTC, Division of Life Sciences and Medicine, University of Science and Technology of China, Hefei, Anhui, China; 5 Department of Nephrology, The Second Hospital of Anhui Medical University, Hefei, Anhui, China

**Keywords:** steatotic liver disease, hepatic steatosis, nonobese population, machine learning, controlled attenuation parameter, risk stratification

## Abstract

**Background:**

Steatotic liver disease affects 40% of nonobese individuals, but existing screening tools inadequately detect and stage disease severity in this population because of the limited sensitivity of conventional ultrasound and the lack of dedicated prediction models.

**Objective:**

This study aimed to develop and validate an interpretable machine learning model specifically for multiclass hepatic steatosis severity prediction in nonobese individuals to support early risk stratification in this underrecognized group.

**Methods:**

Health examination data from 215,145 nonobese participants (BMI <28 kg/m²) were randomly divided into training (n=150,601, 70%) and test (n=64,544, 30%) sets. Hepatic steatosis was diagnosed and graded using the controlled attenuation parameter with established thresholds (none: <248 dB/m; mild: 248‐268 dB/m; and moderate to severe: >268 dB/m). From 42 candidate variables, 14 predictors were selected using Least Absolute Shrinkage and Selection Operator regression and Recursive Feature Elimination based on Random Forest importance. Six machine learning algorithms—k-nearest neighbors, naive Bayes, multilayer perceptron, random forest, support vector machine, and Extreme Gradient Boosting (XGBoost)—were developed using 10-fold cross-validation, with hyperparameters optimized for maximal area under the receiver operating characteristic curve (ROC-AUC). Model interpretability was assessed using Shapley Additive Explanations analysis. External validation was conducted in non-Hispanic Asian participants from the National Health and Nutrition Examination Survey (n=726). Model performance was evaluated using accuracy, Cohen κ, ROC-AUC, area under the precision-recall curve, *F*_1_-score, precision, sensitivity, and specificity.

**Results:**

The final cohort included 215,145 participants, with steatosis severity classified as none (n=92,944, 43.2%), mild (n=54,121, 25.2%), and moderate to severe (n=68,080, 31.6%). Among the 6 machine learning models, XGBoost achieved the best discrimination on the test set, with an accuracy of 0.824 and a macro-average ROC-AUC of 0.941. In external validation, the model maintained strong performance (macro-average ROC-AUC=0.874). Shapley Additive Explanations analysis identified BMI, waist circumference, liver enzymes (alanine aminotransferase and aspartate aminotransferase), renal function indicators (uric acid and serum creatinine), and metabolic indices (triglycerides, continuous metabolic syndrome score, and triglyceride-glucose index) as key contributors to model predictions. The model has been implemented as an online prediction platform to facilitate clinical use.

**Conclusions:**

This interpretable XGBoost model accurately predicts controlled attenuation parameter–defined hepatic steatosis severity in nonobese individuals and demonstrates robust performance in both internal and external validation cohorts, providing a practical tool for early risk stratification in this underrecognized population.

## Introduction

Steatotic liver disease (SLD) is a heterogeneous condition influenced by genetic susceptibility, epigenetic factors, diet, and lifestyle [1]. It has become a leading cause of chronic liver disease globally, with a prevalence of 32.4% that has risen to 37.8% since 2016 [1,2]. Traditional screening strategies have primarily targeted obesity-associated metabolic risks [3]. Recent epidemiological data, however, indicate that up to 40% of SLD cases occur in nonobese individuals, of whom 20% are classified as lean [4], challenging conventional diagnostic paradigms. Despite the absence of overt obesity, these individuals face risks of liver fibrosis, cirrhosis, and cardiometabolic complications comparable to their obese counterparts, with evidence suggesting potentially accelerated disease progression [5-8]. Frequent diagnostic delays, driven by lower clinical suspicion in nonobese individuals, exacerbate this issue. The dissociation between BMI and metabolic dysregulation in this group highlights the urgent need for screening strategies tailored to their distinct metabolic phenotype.

Although liver biopsy remains the diagnostic gold standard, its invasiveness precludes widespread screening. Conventional ultrasound, the primary screening modality, has well-documented limitations, including operator dependence and low sensitivity for detecting mild steatosis (5%‐33% fat content); its accuracy for grading steatosis is only 51.7% [9-11]. The controlled attenuation parameter (CAP), measured via transient elastography using FibroScan (Echosens), provides a more reliable quantitative alternative, enabling standardized classification into 3 severity grades [12-14]. CAP correlates with histological steatosis and metabolic dysfunction severity, offering stratification that guides management—from lifestyle modification for mild disease to combined pharmacological approaches for moderate-to-severe cases [1,15-17]. However, the cost and technical requirements of CAP limit its broad adoption in primary care. Consequently, a diagnostic gap persists, allowing many early-stage nonobese SLD cases to go undetected. This gap underscores the need for nonimaging, cost-effective tools capable of multiclass severity discrimination, particularly for mild steatosis, to facilitate early intervention in metabolically at-risk nonobese populations.

Machine learning (ML) has emerged as a valuable tool for predicting fatty liver disease (FLD) in large-scale studies. For instance, Chen et al [18] and Deng et al [19] used ML models, including Extreme Gradient Boosting (XGBoost), to predict FLD and metabolic dysfunction–associated SLD (MASLD) with high accuracy, achieving areas under the receiver operating characteristic curve (ROC-AUCs) of 0.882 and 0.86, respectively. Other studies by Weng et al [20] and Huang et al [21] further demonstrated that ML models can outperform traditional indices such as the Fatty Liver Index (FLI) in FLD detection. A notable advance by Su et al [22] developed a neural network model specifically for lean individuals (BMI <23 kg/m²), reporting an ROC-AUC of 0.885. While representing progress in population-specific modeling, this approach was limited to binary classification and relied on ultrasound—a modality with recognized sensitivity limitations, especially for mild steatosis. These limitations highlight the need for models capable of multiclass severity stratification in nonobese populations based on a more sensitive quantitative standard.

To address these gaps, this study aimed to develop and validate an optimal multiclass ML model for predicting hepatic steatosis severity in nonobese populations, using large-scale health examination data from Eastern China. Six ML models were constructed and compared. The proposed approach offers several key advancements: (1) the use of CAP for accurate steatosis grading, overcoming the sensitivity limitations of ultrasound; (2) a specific focus on the underrecognized nonobese population; (3) multiclass severity prediction (none, mild, and moderate to severe) for precise risk stratification; and (4) deployment as an open-access online platform offering both a full and a simplified model to accommodate varied resource settings. By enabling multiclass risk stratification in nonobese individuals, this work provides a practical tool to support early identification and may facilitate a shift from passive imaging-based diagnosis toward more proactive metabolic risk assessment.

## Methods

### Study Population

Retrospective health examination data from 269,240 individuals (2018‐2024) were obtained from the Health Management Center database of the First Affiliated Hospital of University of Science and Technology of China*.* After applying inclusion criteria (completion of transient elastography, BMI <28 kg/m²) and exclusion criteria (missing BMI, age <18 or >100 y, pregnancy, liver cirrhosis, liver tumors, or other malignancies), 215,145 participants were included. The BMI cutoff of <28 kg/m² aligns with the Chinese adult obesity threshold (BMI ≥28 kg/m²) defined by the Working Group on Obesity in China [23]. This cutoff is also consistent with the Asian-specific obesity criterion (27.5 kg/m²) recommended in international guidelines [24], reflecting the distinct anthropometric and metabolic profile of Asian populations.

For the external validation cohort extracted from the National Health and Nutrition Examination Survey (NHANES) 2017 to 2020 cycle, only non-Hispanic Asian participants with complete data for all variables required by the final model (including CAP, anthropometric measures, laboratory tests, and derived composite indices) were retained. Participants with any missing values in these key variables were excluded, resulting in a final sample of 726 individuals with fully observed data.

### Ethical Considerations

This study was conducted in accordance with the Declaration of Helsinki and was approved by the Ethics Committee of the First Affiliated Hospital of University of Science and Technology of China (approval 2024-RE-312). The requirement for obtaining individual informed consent was waived by the approving ethics committee. All personal identifiers were removed prior to analysis to protect participant privacy and confidentiality. No compensation was provided for this secondary use of data. The manuscript and supplementary materials contain no personally identifiable information of any participant.

### Grading Diagnosis of Hepatic Steatosis

Hepatic steatosis was diagnosed and graded using CAP measured via transient elastography. CAP quantifies ultrasound wave attenuation, correlating with liver fat content. Severity was graded using established thresholds: none (CAP <248 dB/m), mild (248‐268 dB/m), and moderate to severe (>268 dB/m) [12]. The reliability of CAP is well established; validation against liver biopsy in multicenter studies has shown excellent ROC-AUC (0.87 for ≥S1 steatosis) [13]. This quantitative approach ensures objective and reproducible assessment, overcoming limitations of conventional ultrasonography.

### Variable Processing and Selection

Initially, 42 potential predictors were collected from questionnaires and routine examinations. Variables with >30% missing data were excluded. The missing data pattern in the final cohort (n=215,145) is shown in Multimedia Appendix 1. After splitting the data into training (n=150,601, 70%) and test (n=64,544, 30%) sets, missing values were handled using multiple imputation with the random forest (RF) algorithm (mice package, method=“rf”). To account for imputation uncertainty, we generated 5 independent imputed datasets from the training set only. The imputation models learned from the training set were then applied once to the test set—this reflects real deployment, where new data are observed only once, while multiple imputation at the training stage and the subsequent model ensemble already capture missing data uncertainty. Continuous features were normalized using z-score standardization based on training set means and SDs, with the same parameters applied to the test set. Categorical features were label encoded to avoid high dimensionality from one-hot encoding.

Additionally, the following composite indicators were calculated: the aspartate aminotransferase (AST)-to-alanine aminotransferase ratio (ALT); mean arterial pressure as (2×diastolic blood pressure+systolic blood pressure [SBP])/3; the continuous metabolic syndrome score (CMetS), derived from weighted calculations of waist circumference (WC), high-density lipoprotein (HDL) cholesterol, mean arterial pressure, triglycerides, and fasting blood glucose (FBG) [25]; and the triglyceride-glucose index (TyG) as Ln[fasting triglycerides (mg/dL)×fasting glucose (mg/dL)/2]. As the original laboratory measurements of FBG and triglycerides in our cohort were recorded in mmol/L, these values were converted to mg/dL prior to TyG calculation by multiplying by the conversion factors: 18 for glucose and 88.57 for triglycerides.

### Feature Selection

To identify key predictors robust to imputation uncertainty, feature selection was performed within each of the 5 imputed training datasets. In each dataset, 2 complementary methods were applied in parallel: Least Absolute Shrinkage and Selection Operator (LASSO) regression with 10-fold cross-validation and Recursive Feature Elimination based on Random Forest importance (RFE-RF). For a given dataset, the intersection of variables selected by LASSO and RFE-RF was taken as its candidate set. Across the 5 imputed datasets, variables that appeared in at least three candidate sets were retained as the final predictors.

### Model Development and Explanation

After multiple imputation, 5 complete training datasets were generated. For each ML algorithm (XGBoost, multilayer perceptron [MLP], k-nearest neighbors [KNN], RF, support vector machine [SVM], and naive Bayes [NB]), we trained 5 separate models—one on each imputed training set. Each of these 5 models then produced predicted probabilities for all classes on the test set. To account for uncertainty arising from missing data imputation, we pooled these predictions by averaging the 5 probability vectors for each test sample, yielding a single pooled probability per class. The final predicted class was assigned as the class with the highest average probability. All performance metrics (accuracy, Cohen κ, macro-average ROC-AUC, macro-average PR-AUC, as well as class-wise precision, recall, specificity, and *F*_1_-score) were derived from these pooled predictions. To enhance interpretability, Shapley Additive Explanations (SHAP) analysis was applied to the best-performing XGBoost model. Grounded in game theory, SHAP values quantify the magnitude and direction of each feature’s contribution to individual predictions, providing a mathematically consistent framework for model explanation.

### Statistical Analysis

All statistical analyses were performed using R software and Python. The Kolmogorov-Smirnov test assessed normality for continuous variables. For nonnormally distributed data, group comparisons used the Kruskal-Wallis H test (multiple groups) or Mann-Whitney *U* test (2 groups). Categorical variables were summarized as frequencies and percentages, with differences assessed by the Pearson chi-square test. Two-sided *P* values <.05 were considered statistically significant.

## Results

### Basic Characteristics

**Figure 1.**
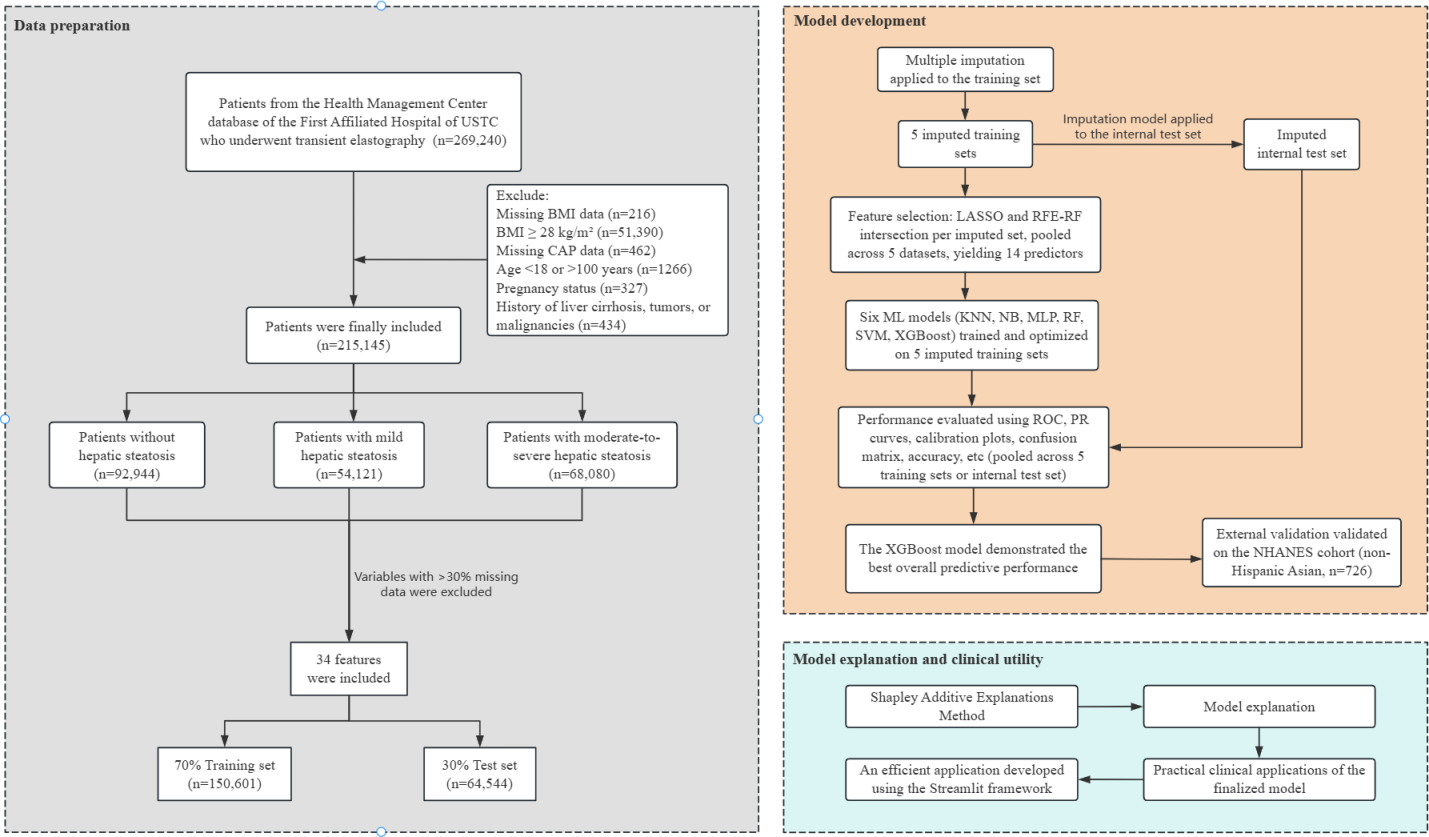
Study flowchart of participant selection and model development workflow. CAP: controlled attenuation parameter; KNN: k-nearest neighbors; LASSO: Least Absolute Shrinkage and Selection Operator; MLP: multilayer perceptron; NB: naive Bayes; NHANES: National Health and Nutrition Examination Survey; RF: random forest; RFE-RF: Recursive Feature Elimination based on Random Forest importance; ROC: receiver operating characteristic; SVM: support vector machine; USTC: University of Science and Technology of China; XGBoost: Extreme Gradient Boosting.

The final cohort comprised 215,145 participants, stratified by hepatic steatosis grade: none (n=92,944, 43.2%), mild (n=54,121, 25.2%), and moderate to severe (n=68,080, 31.6%). The cohort had a median age of 44 (IQR 34‐54) years, a balanced sex distribution (men: 51.07%; Table 1). The distributions of the 6 most influential predictors across grades are shown in Multimedia Appendix 2, displaying clear, clinically expected trends. The training and test sets exhibited balanced baseline characteristics (Multimedia Appendix 3). Density plots confirmed that the distributions of key variables remained consistent before and after multiple imputation (Multimedia Appendix 4). Detailed information about the research design is available in Figure 1.

**Table 1.** Baseline characteristics of participants stratified by hepatic steatosis grade.

Variables	Total (N=215,145)	None (n=92,944)	Mild (n=54,121)	Moderate to severe (n=68,080)	*P*^a^ value
Age (y), median (IQR)	44 (34 to 55)	42 (32 to 53)	46 (35 to 55)	46 (36 to 55)	<.001
Gender, n (%)					<.001
Female	1,03,979 (48.33)	48,089 (51.74)	25,594 (47.29)	30,296 (44.50)	
Male	1,09,875 (51.07)	44,269 (47.63)	28,213 (52.13)	37,393 (54.9)	
Miss	1291 (0.60)	586 (0.63)	314 (0.58)	391 (0.58)	
CAP^b^ (dB/m), median (IQR)	251 (227 to 275)	224 (212 to 233)	257 (250 to 262)	283 (276 to 291)	<.001
LSM^c^ (kPa), median (IQR)	5.80 (4.90 to 6.70)	5.50 (4.70 to 6.40)	5.80 (5 to 6.70)	6.20 (5.30 to 7.10)	<.001
Miss, n (%)	27,582 (12.82)	12,403 (13.34)	6897 (12.74)	8282 (12.17)	
WC^d^ (cm), median (IQR)	88 (83 to 92)	83 (79 to 87)	89 (86 to 91)	92 (90 to 95)	<.001
BMI (kg/m^2^), median (IQR)	24.30 (22.47 to 25.90)	22.47 (20.93 to 23.90)	24.50 (23.40 to 25.70)	26.10 (25.10 to 27)	<.001
SBP^e^ (mm Hg), median (IQR)	124 (113 to 136)	120 (110 to 131)	125 (115 to 137)	128 (118 to 140)	<.001
Miss, n (%)	49,419 (22.97)	24,317 (26.16)	13,488 (24.92)	11,614 (17.06)	
DBP^f^ (mm Hg), median (IQR)	77 (70 to 85)	74 (67 to 82)	78 (71 to 85)	81 (73 to 88)	<.001
Miss, n (%)	6648 (3.09)	3592 (3.86)	1914 (3.54)	1142 (1.68)	
Lym^g^ (%), median (IQR)	33.60 (28.70 to 38.70)	33.70 (28.70 to 38.90)	33.30 (28.60 to 38.50)	33.50 (28.80 to 38.40)	<.001
Miss, n (%)	59,380 (27.6)	26,893 (28.93)	15,327 (28.32)	17,160 (25.21)	
PLT^h^ (×10⁹/L)	224 (191 to 261)	221 (188 to 258.75)	223 (191 to 260)	229 (195 to 266)	<.001
Miss, n (%)	61,962 (28.8)	27,789 (29.9)	15,876 (29.33)	18,297 (26.88)	
Mono^i^ (%), median (IQR)	6.50 (5.50 to 7.70)	6.50 (5.50 to 7.60)	6.60 (5.60 to 7.70)	6.60 (5.60 to 7.70)	<.001
Miss, n (%)	61,295 (28.49)	27,543 (29.63)	15,832 (29.25)	17,920 (26.32)	
Neu^j^ (%), median (IQR)	56.60 (51.20 to 61.80)	56.50 (51 to 62)	56.70 (51.50 to 61.80)	56.50 (51.40 to 61.60)	.08
Miss, n (%)	59,272 (27.55)	26,987 (29.04)	15,234 (28.15)	17,051 (25.05)	
WBC^k^ (×10⁹/L), median (IQR)	5.85 (5 to 6.84)	5.56 (4.74 to 6.52)	5.89 (5.08 to 6.88)	6.19 (5.35 to 7.17)	<.001
Miss, n (%)	54,797 (25.47)	26,714 (28.74)	15,189 (28.06)	12,894 (18.94)	
Hb^l^ (g/L), median (IQR)	148 (136 to 156)	142 (131 to 153)	149 (140 to 157)	153 (145 to 160)	<.001
Miss, n (%)	63,339 (29.44)	27,853 (29.97)	16,207 (29.95)	19,279 (28.32)	
FBG^m^ (mmol/L), median (IQR)	5.08 (4.76 to 5.47)	4.96 (4.68 to 5.29)	5.11 (4.80 to 5.50)	5.23 (4.89 to 5.72)	<.001
Miss, n (%)	10,004 (4.65)	5396 (5.81)	3012 (5.57)	1596 (2.34)	
Triglycerides (mmol/L), median (IQR)	1.32 (0.93 to 1.92)	1.05 (0.78 to 1.45)	1.40 (1.02 to 1.96)	1.76 (1.26 to 2.53)	<.001
Miss, n (%)	3249 (1.51)	1807 (1.94)	893 (1.65)	549 (0.81)	
TC^n^ (mmol/L), median (IQR)	4.78 (4.22 to 5.39)	4.68 (4.14 to 5.29)	4.83 (4.27 to 5.44)	4.88 (4.33 to 5.49)	<.001
Miss, n (%)	39,479 (18.35)	19,428 (20.9)	10,973 (20.27)	9078 (13.33)	
HDL^o^ cholesterol (mmol/L), median (IQR)	1.13 (0.96 to 1.35)	1.24 (1.05 to 1.47)	1.10 (0.94 to 1.30)	1.02 (0.88 to 1.18)	<.001
Miss, n (%)	9488 (4.41)	5114 (5.5)	2786 (5.15)	1588 (2.33)	
LDL^p^ cholesterol (mmol/L), median (IQR)	2.84 (2.35 to 3.36)	2.71 (2.25 to 3.21)	2.91 (2.43 to 3.42)	2.97 (2.47 to 3.49)	<.001
Miss, n (%)	1441 (0.67)	794 (0.85)	406 (0.75)	241 (0.35)	
VLDL^q^ cholesterol (mmol/L), median (IQR)	0.72 (0.54 to 0.95)	0.67 (0.51 to 0.87)	0.73 (0.55 to 0.97)	0.79 (0.58 to 1.07)	<.001
Miss, n (%)	51,592 (23.98)	25,213 (27.13)	14,286 (26.4)	12,093 (17.76)	
HbA_1c_^r^ (%), median (IQR)	5.50 (5.30 to 5.80)	5.50 (5.30 to 5.70)	5.60 (5.40 to 5.80)	5.60 (5.40 to 6)	<.001
Miss, n (%)	58,197 (27.05)	26,345 (28.35)	15,234 (28.15)	16,618 (24.41)	
ALT^s^ (U/L), median (IQR)	21 (15.50 to 31)	18 (13 to 24)	22 (16 to 30)	28 (20 to 41)	<.001
Miss, n (%)	559 (0.26)	308 (0.33)	164 (0.3)	87 (0.13)	
AST^t^ (U/L), median (IQR)	22 (19 to 26.70)	21 (18 to 25)	22 (19 to 26)	24 (20 to 29)	<.001
Miss, n (%)	387 (0.18)	219 (0.24)	108 (0.2)	60 (0.09)	
GGT^u^ (U/L), median (IQR)	22.80 (15 to 36.20)	17.70 (12.90 to 26.10)	24 (16.70 to 37)	32 (21.90 to 51)	<.001
Miss, n (%)	60,972 (28.34)	27,489 (29.58)	15,930 (29.43)	17,553 (25.78)	
ALP^v^ (IU/L), median (IQR)	72 (61 to 86)	69 (58 to 83)	73 (62 to 87)	75 (64 to 88)	<.001
Miss, n (%)	5658 (2.63)	3089 (3.32)	1713 (3.17)	856 (1.26)	
BUN^w^ (mmol/L), median (IQR)	5.26 (4.49 to 6.14)	5.16 (4.38 to 6.07)	5.31 (4.56 to 6.19)	5.35 (4.60 to 6.21)	<.001
Miss, n (%)	57,766 (26.85)	26,123 (28.11)	15,098 (27.9)	16,545 (24.3)	
SCr^x^ (μmol/L), median (IQR)	69 (60 to 78)	67 (56 to 76)	71 (62 to 79)	71 (63 to 79)	<.001
Miss, n (%)	34,509 (16.04)	17,023 (18.32)	9576 (17.69)	7910 (11.62)	
UA^y^ (μmol/L), median (IQR)	360.70 (303 to 419)	329 (275 to 387)	368.70 (315.48 to 422)	395 (342 to 451)	<.001
Miss, n (%)	51,656 (24.01)	25,196 (27.11)	14,312 (26.44)	12,148 (17.84)	
AFP^z^ (ng/mL), median (IQR)	2.80 (2 to 3.96)	2.67 (1.93 to 3.83)	2.88 (2.04 to 4.07)	2.91 (2.08 to 4.06)	<.001
Miss, n (%)	49,032 (22.79)	23,987 (25.81)	13,612 (25.15)	11,433 (16.79)	
CEA^aa^ (ng/mL), median (IQR)	1.69 (1.18 to 2.43)	1.64 (1.13 to 2.39)	1.72 (1.21 to 2.46)	1.74 (1.23 to 2.47)	<.001
Miss, n (%)	30,271 (14.07)	14,986 (16.12)	8414 (15.55)	6871 (10.09)	
ALB^ab^ (g/L), median (IQR)	46 (44.30 to 47.70)	45.70 (44 to 47.50)	46 (44.30 to 47.60)	46.40 (44.80 to 48.10)	<.001
Miss, n (%)	61,574 (28.62)	27,589 (29.68)	15,966 (29.5)	18,019 (26.47)	
Tbli^ac^ (μmol/L), median (IQR)	14.30 (11.30 to 18.20)	14.20 (11.20 to 18.10)	14.30 (11.40 to 18.20)	14.50 (11.50 to 18.40)	<.001
Miss, n (%)	61,510 (28.59)	27,498 (29.59)	15,981 (29.53)	18,031 (26.49)	
Dbli^ad^ (μmol/L), median (IQR)	4.10 (2.90 to 5.50)	4 (2.80 to 5.50)	4.10 (2.90 to 5.40)	4.20 (3.10 to 5.50)	<.001
Miss, n (%)	61,144 (28.42)	27,345 (29.42)	15,876 (29.33)	17,923 (26.33)	
Ibli^ae^ (μmol/L), median (IQR)	10.30 (8 to 13.30)	10.20 (8 to 13.20)	10.30 (8.10 to 13.40)	10.30 (8.00 to 13.30)	.01
Miss, n (%)	54,840 (25.49)	25,432 (27.36)	14,123 (26.1)	15,285 (22.45)	
HBP^af^, n (%)					<.001
No	1,20,761 (56.13)	61,743 (66.43)	27,548 (50.90)	31,470 (46.23)	
Yes	78,808 (36.63)	24,426 (26.28)	20,956 (38.72)	33,426 (49.10)	
Miss	15,576 (7.24)	6775 (7.29)	5617 (10.38)	3184 (4.67)	
TyG^ag^, median (IQR)	8.68 (8.29 to 9.10)	8.35 (8.04 to 8.69)	8.68 (8.34 to 9.04)	8.97 (8.61 to 9.37)	<.001
Miss, n (%)	11,682 (5.43)	5595 (6.02)	3058 (5.65)	3029 (4.45)	
CMetS^ah^, median (IQR)	0.33 (−0.10 to 0.78)	−0.12 (−0.44 to 0.23)	0.29 (−0.03 to 0.64)	0.71 (0.36 to 1.12)	<.001
Miss, n (%)	50,925 (23.67)	24,686 (26.56)	13,579 (25.09)	12,660 (18.60)	

a *P* values for continuous variables were calculated using the Kruskal-Wallis H test, and those for categorical variables were calculated using Pearson chi-square test.

b CAP: controlled attenuation parameter.

c LSM: liver stiffness measurement.

d WC: waist circumference.

e SBP: systolic blood pressure.

f DBP: diastolic blood pressure.

g Lym: lymphocyte percentage.

h PLT: platelet count.

i Mono: monocyte percentage.

j Neu: neutrophil percentage.

k WBC: white blood cell count.

l Hb: hemoglobin.

m FBG: fasting blood glucose.

n TC: total cholesterol.

o HDL: high-density lipoprotein.

p LDL: low-density lipoprotein.

q VLDL: very-low-density lipoprotein.

r HbA_1c_: hemoglobin A_1c_.

s ALT: alanine aminotransferase.

t AST: aspartate aminotransferase.

u GGT: gamma-glutamyl transferase.

v ALP: alkaline phosphatase.

w BUN: blood urea nitrogen.

x SCr: serum creatinine.

y UA: uric acid.

z AFP: alpha-fetoprotein.

aa CEA: carcinoembryonic antigen.

ab ALB: albumin.

ac Tbli: total bilirubin.

ad Dbli: direct bilirubin.

ae Ibli: indirect bilirubin.

af HBP: high blood pressure.

ag TyG: triglyceride-glucose index.

ah CMetS: continuous metabolic syndrome score.

### Feature Selection

Following the multiple imputation–based feature selection procedure, 14 predictors were consistently identified as robust determinants of hepatic steatosis severity in the nonobese population. These final selected variables encompass multiple physiological dimensions: anthropometric measures (BMI and WC), liver enzymes (ALT, AST, and alkaline phosphatase [ALP]), renal function indicators (uric acid [UA] and serum creatinine [SCr]), lipid profiles (triglycerides and low-density lipoprotein [LDL] cholesterol), metabolic composites (CMetS and TyG), as well as age, SBP, and albumin. Some individual metabolic parameters (eg, high-density lipoprotein cholesterol and FBG) were likely excluded as their collective information was more comprehensively represented by the selected composite measure, CMetS. LASSO coefficient paths, cross-validation error curves for optimal lambda selection, and RFE-RF accuracy curves are presented in Multimedia Appendix 5.

### Model Performance Comparisons

**Figure 2.**
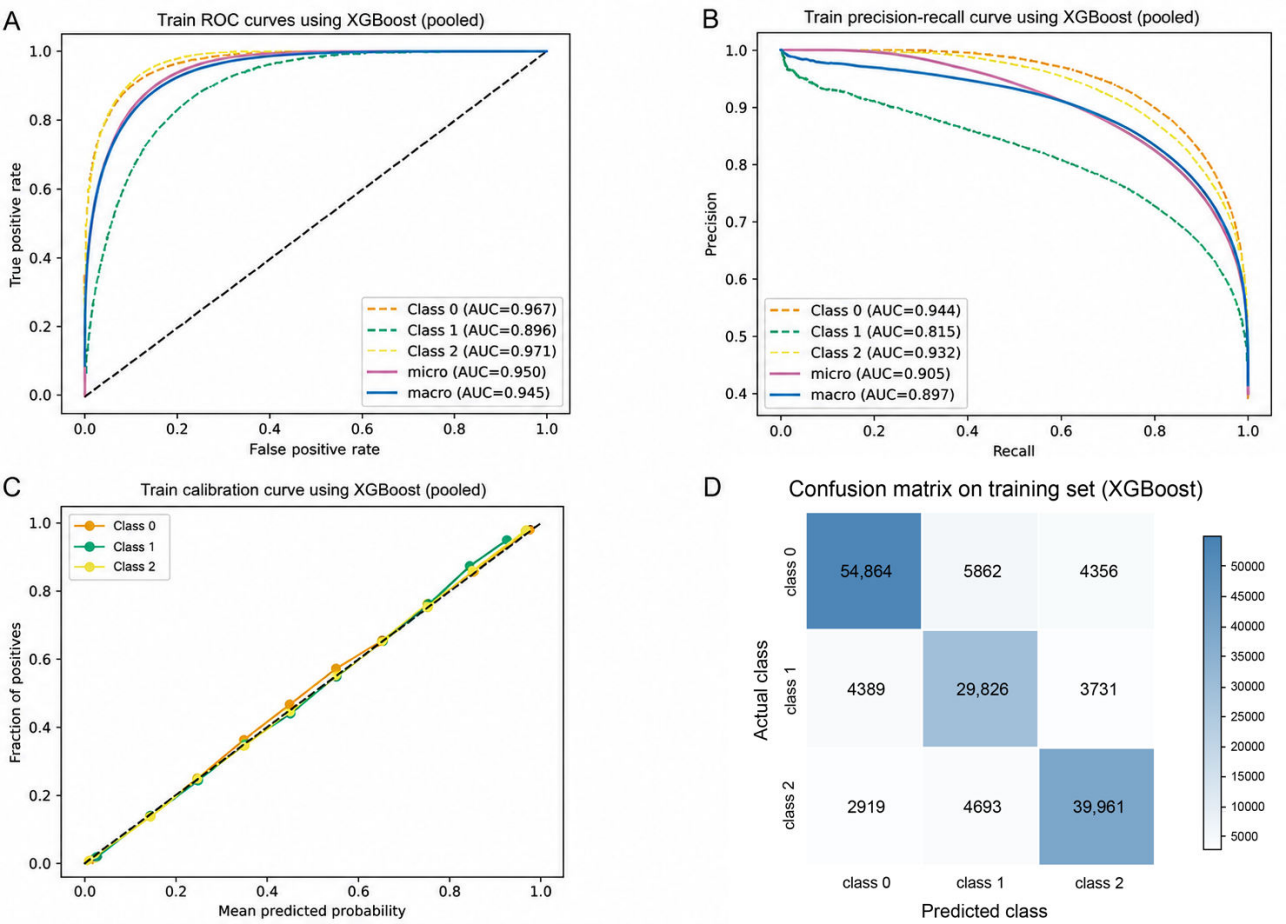
Performance of the XGBoost (Extreme Gradient Boosting) model in the training set. AUC: area under the curve; ROC: receiver operating characteristic.

**Figure 3.**
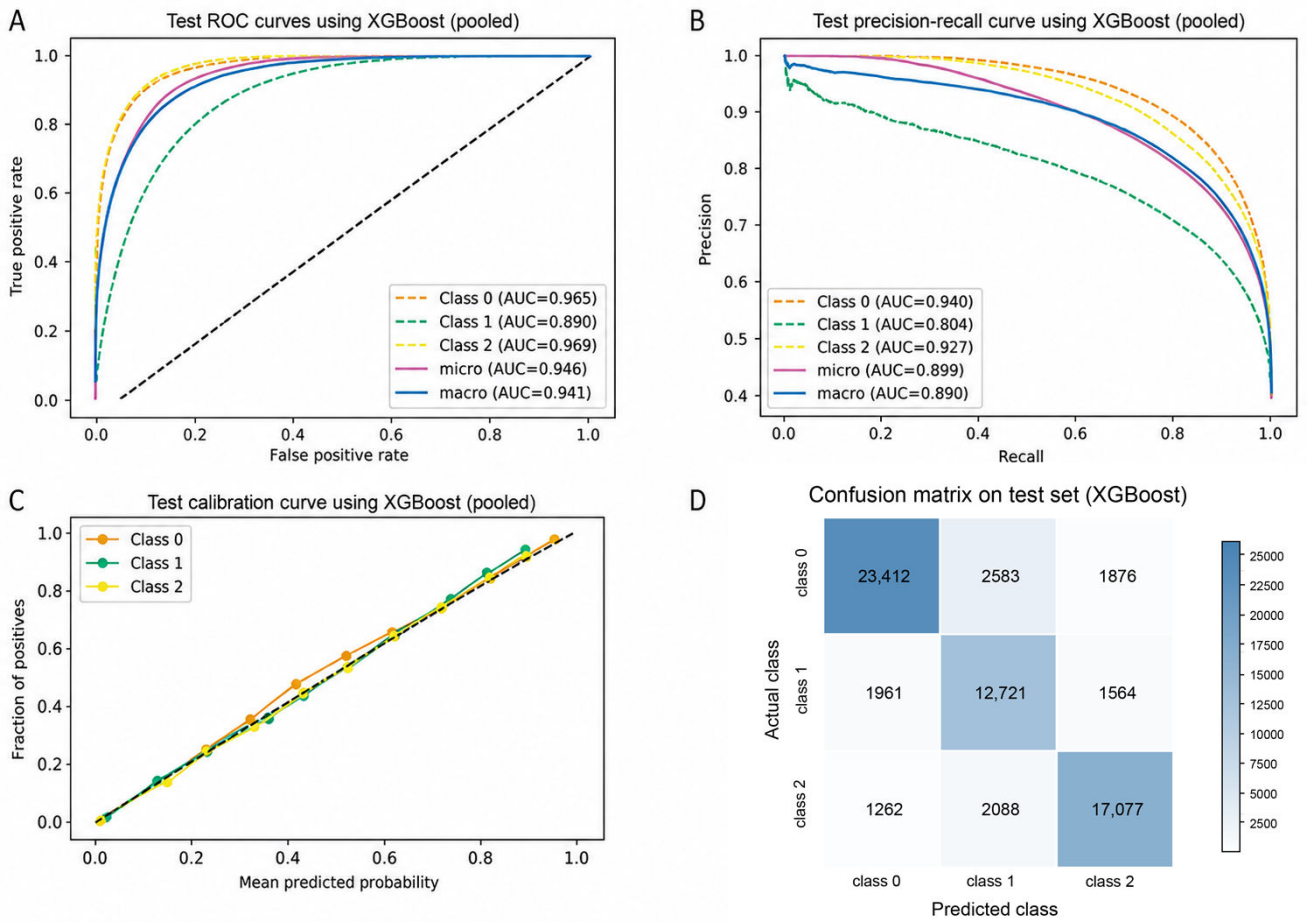
Performance of the XGBoost (Extreme Gradient Boosting) model in the test set. AUC: area under the curve; ROC: receiver operating characteristic.

Six ML models were developed for multiclass severity stratification (none, mild, and moderate to severe). All models demonstrated robust performance. In the training set, KNN achieved perfect discrimination (macro-average ROC-AUC=1.000) due to overfitting, followed by RF (0.959), XGBoost (0.945), MLP (0.942), SVM (0.917), and NB (0.913; Table 2 and Figure 2A). In the test set, XGBoost, MLP, and RF tied for the highest macroaverage ROC-AUC, each attaining 0.941 (Table 2 and Figure 3A). They were followed by KNN (0.934), SVM (0.917), and NB (0.913).

**Table 2.** Performance of each algorithm in the training and test set.

Performance metric	Training set						Test set					
KNN^a^	MLP^b^	NB^c^	RF^d^	SVM^e^	XGBoost^f^	KNN	MLP	NB	RF	SVM	XGBoost
Accuracy	0.998	0.823	0.766	0.847	0.799	0.828	0.810	0.821	0.767	0.817	0.798	0.824
Cohen_Kappa	0.997	0.716	0.640	0.760	0.657	0.721	0.700	0.712	0.641	0.711	0.654	0.713
macro_ROC_AUC^g^	1.000	0.942	0.913	0.959	0.917	0.945	0.934	0.941	0.913	0.941	0.917	0.941
micro_ROC_AUC	1.000	0.947	0.918	0.962	0.921	0.950	0.940	0.946	0.918	0.946	0.921	0.946
macro_PR_AUC^h^	1.000	0.892	0.831	0.924	0.841	0.897	0.875	0.890	0.831	0.890	0.839	0.890
micro_PR_AUC	1.000	0.900	0.845	0.929	0.851	0.905	0.886	0.898	0.845	0.898	0.849	0.899
Precision (none)	0.999	0.879	0.824	0.893	0.882	0.882	0.867	0.878	0.824	0.874	0.877	0.879
Recall (none)	0.999	0.849	0.834	0.862	0.872	0.843	0.827	0.849	0.836	0.838	0.875	0.840
Specificity (none)	0.999	0.911	0.864	0.921	0.911	0.915	0.904	0.911	0.864	0.908	0.907	0.912
*F*_1_-score (none)	0.999	0.863	0.829	0.877	0.877	0.862	0.847	0.864	0.830	0.855	0.876	0.859
ROC_AUC (none)	1.000	0.966	0.950	0.976	0.948	0.967	0.960	0.965	0.950	0.965	0.947	0.965
Precision (mild)	0.996	0.737	0.662	0.740	0.686	0.739	0.727	0.725	0.657	0.718	0.675	0.731
Recall (mild)	0.997	0.751	0.770	0.813	0.636	0.786	0.761	0.745	0.769	0.766	0.630	0.783
Specificity (mild)	0.999	0.910	0.868	0.904	0.902	0.906	0.904	0.905	0.865	0.899	0.898	0.903
F_1_-score (mild)	0.997	0.744	0.712	0.775	0.660	0.762	0.744	0.735	0.709	0.741	0.652	0.756
ROC_AUC (mild)	1.000	0.891	0.839	0.924	0.843	0.896	0.877	0.889	0.839	0.890	0.843	0.890
Precision (moderate to severe)	0.999	0.820	0.784	0.881	0.773	0.832	0.803	0.825	0.794	0.826	0.782	0.832
Recall (moderate to severe)	0.998	0.846	0.669	0.854	0.830	0.840	0.825	0.844	0.671	0.828	0.827	0.836
Specificity (moderate to severe)	0.999	0.914	0.915	0.947	0.887	0.922	0.907	0.917	0.919	0.919	0.893	0.922
*F*_1_-score (moderate to severe)	0.998	0.833	0.722	0.867	0.800	0.836	0.814	0.835	0.727	0.827	0.804	0.834
ROC_AUC (moderate to severe)	1.000	0.969	0.951	0.977	0.960	0.971	0.965	0.969	0.951	0.969	0.960	0.969

a KNN: k-nearest neighbors.

b MLP: multilayer perceptron.

c NB: naive Bayes.

d RF: random forest.

e SVM: support vector machine.

f XGBoost: Extreme Gradient Boosting.

g ROC-AUC: area under the receiver operating characteristic curve.

h PR-AUC: area under the precision-recall curve.

Precision-recall (PR) curves were analyzed to evaluate performance under class imbalance (Figure 2B for training and Figure 3B for test set of XGBoost). In the training set, KNN exhibited a perfect macro-average PR-AUC (1.000) as a result of overfitting. Among the remaining models, RF achieved the highest macro-average PR-AUC (0.924), followed by XGBoost (0.897), MLP (0.892), SVM (0.841), and NB (0.831). In the test set, XGBoost, MLP, and again RF achieved identical macro-average PR-AUC values of 0.890, marginally outperforming KNN (0.875), SVM (0.839), and NB (0.831). Calibration assessments (Figures 2C and 3C) demonstrated that XGBoost’s predicted probabilities showed good alignment with observed outcomes. Its confusion matrix (Figures 2D and 3D) also exhibited a favorable pattern, characterized by relatively low misclassification density and fewer severe grading errors between adjacent categories. Corresponding figures for the other models are presented in Multimedia Appendix 6, and complete performance data across all imputed datasets are provided in Multimedia Appendix 7.

As detailed in Table 2, XGBoost achieved the best overall performance on the test set. It attained the highest macroaverage PR-AUC (0.890) and macroaverage ROC-AUC (0.941), along with an accuracy of 0.824 and a Cohen κ of 0.713. Although KNN showed exceptional training set performance (macro ROC-AUC=1.000), its test set performance declined to 0.934, indicating a degree of overfitting. While the MLP and RF were competitive in several metrics (macro-ROC-AUC=0.941 and macro-PR-AUC=0.890), XGBoost was selected for further analysis due to its consistently superior performance across all 3 severity classes, particularly demonstrating higher precision and sensitivity for the clinically important mild steatosis category—essential for early detection—as well as its stronger interpretability via SHAP and lower computational cost. Optimal hyperparameters for all models are provided in Multimedia Appendix 8.

### Model Interpretation

**Figure 4.**
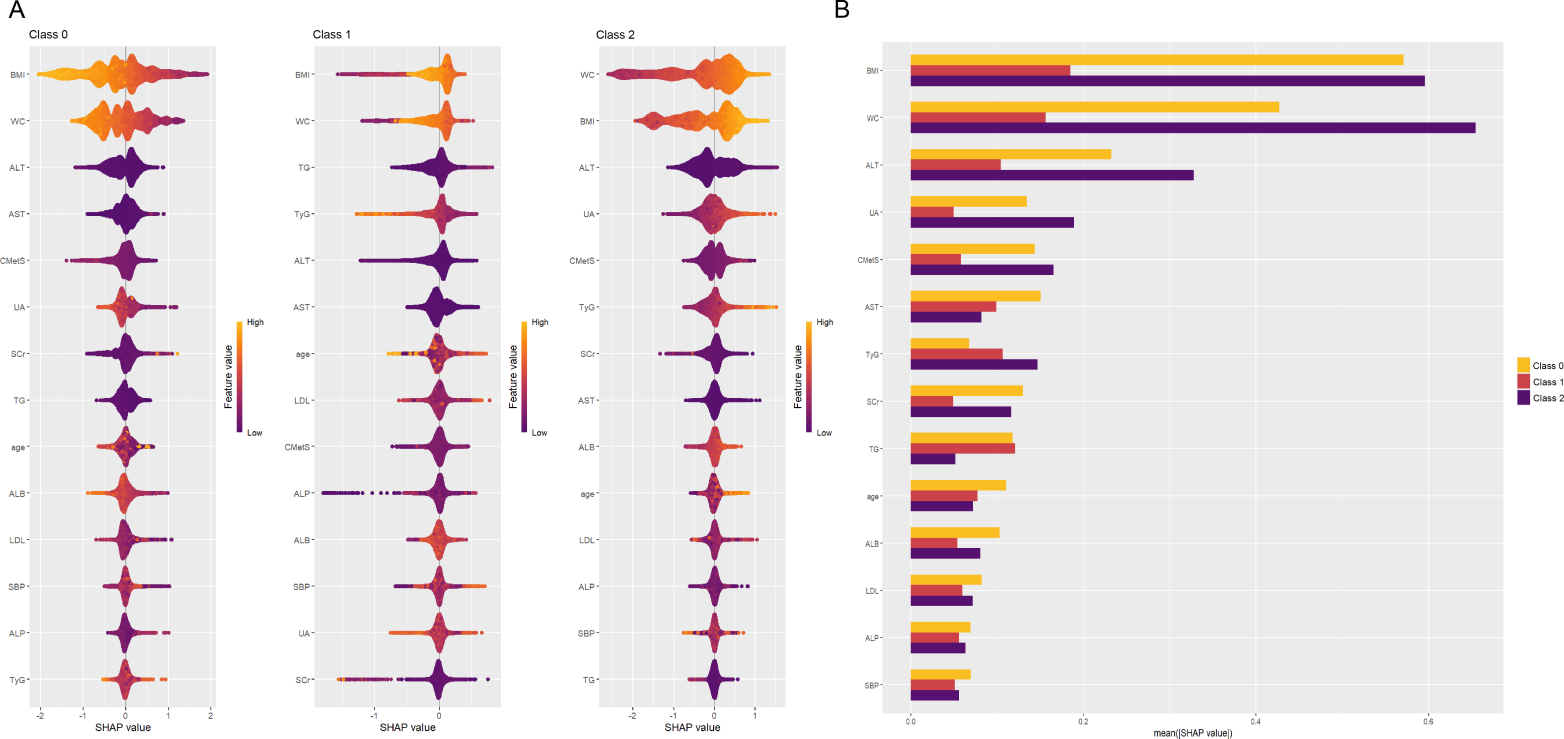
Global model interpretability using Shapley Additive Explanations. ALB: albumin; ALP: alkaline phosphatase; ALT: alanine aminotransferase; AST: aspartate aminotransferase; CMetS: continuous metabolic syndrome score; LDL: low-density lipoprotein cholesterol; SBP: systolic blood pressure; SCr: serum creatinine; SHAP: Shapley Additive Explanations; TG: triglyceride; TyG: triglyceride-glucose index; UA: uric acid; WC: waist circumference.

SHAP analysis was used on the optimal XGBoost model to provide global and local explanations of its decision logic. The bee swarm plot (Figure 4A) shows the distribution and impact direction of SHAP values per feature across samples; and the bar plot (Figure 4B) ranks features by mean absolute SHAP value. Both analyses identified BMI, WC, ALT, and UA as the most influential predictors across all severity grades. A positive SHAP value indicates that a higher feature value pushes the prediction toward a more severe category (eg, elevated BMI, WC, and TyG increased the predicted risk of moderate-to-severe steatosis).

**Figure 5.**
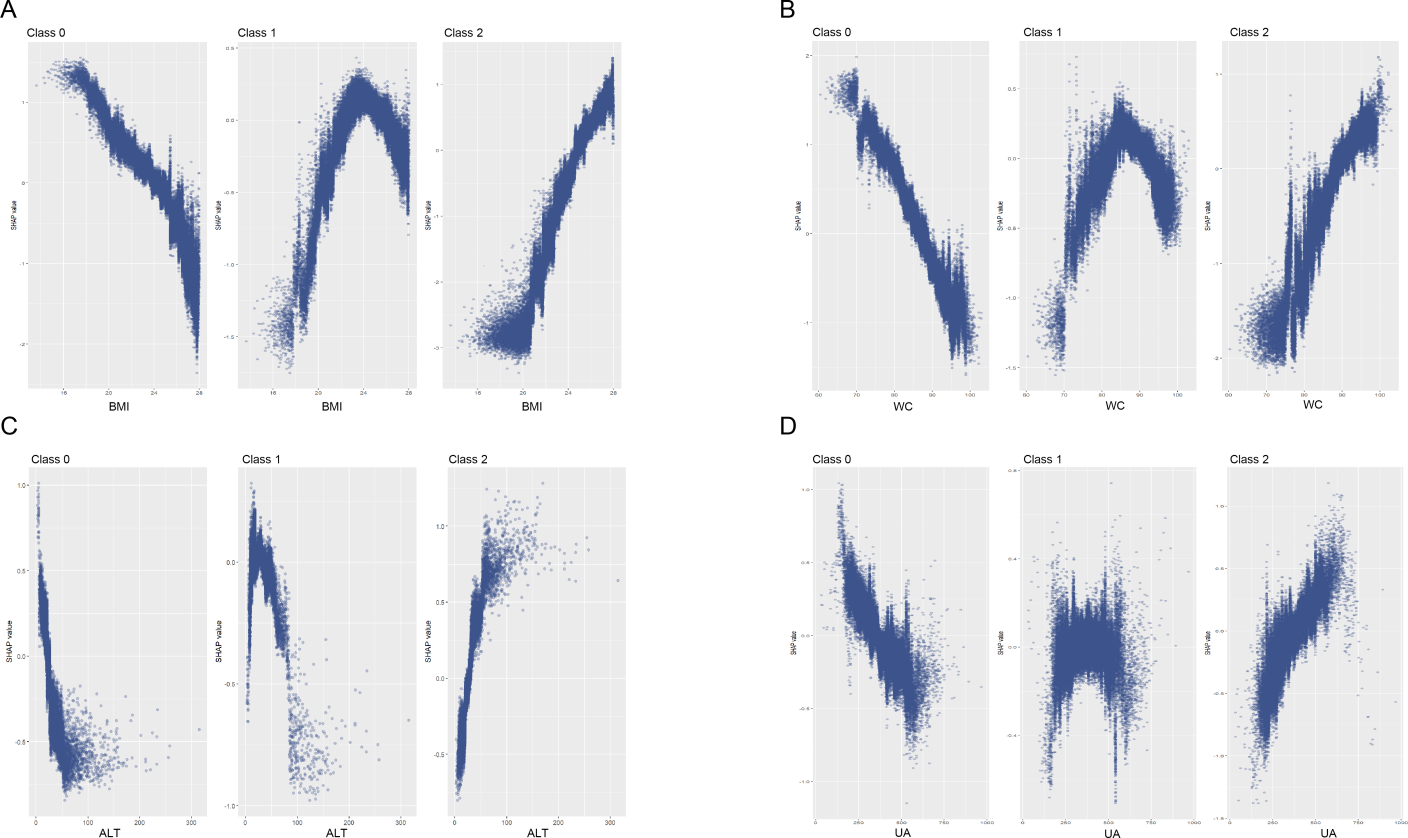
Shapley Additive Explanations dependence plots for key predictors. ALT: alanine aminotransferase; SHAP: Shapley Additive Explanations; UA: uric acid; WC: waist circumference.

SHAP dependence plots for the 4 most important features (Figure 5) illustrate the nonlinear relationships between feature values and their SHAP contributions. Consistently, elevated levels of BMI, WC, ALT, and UA were associated with positive SHAP values, increasing the predicted risk for moderate-to-severe steatosis.

**Figure 6.**
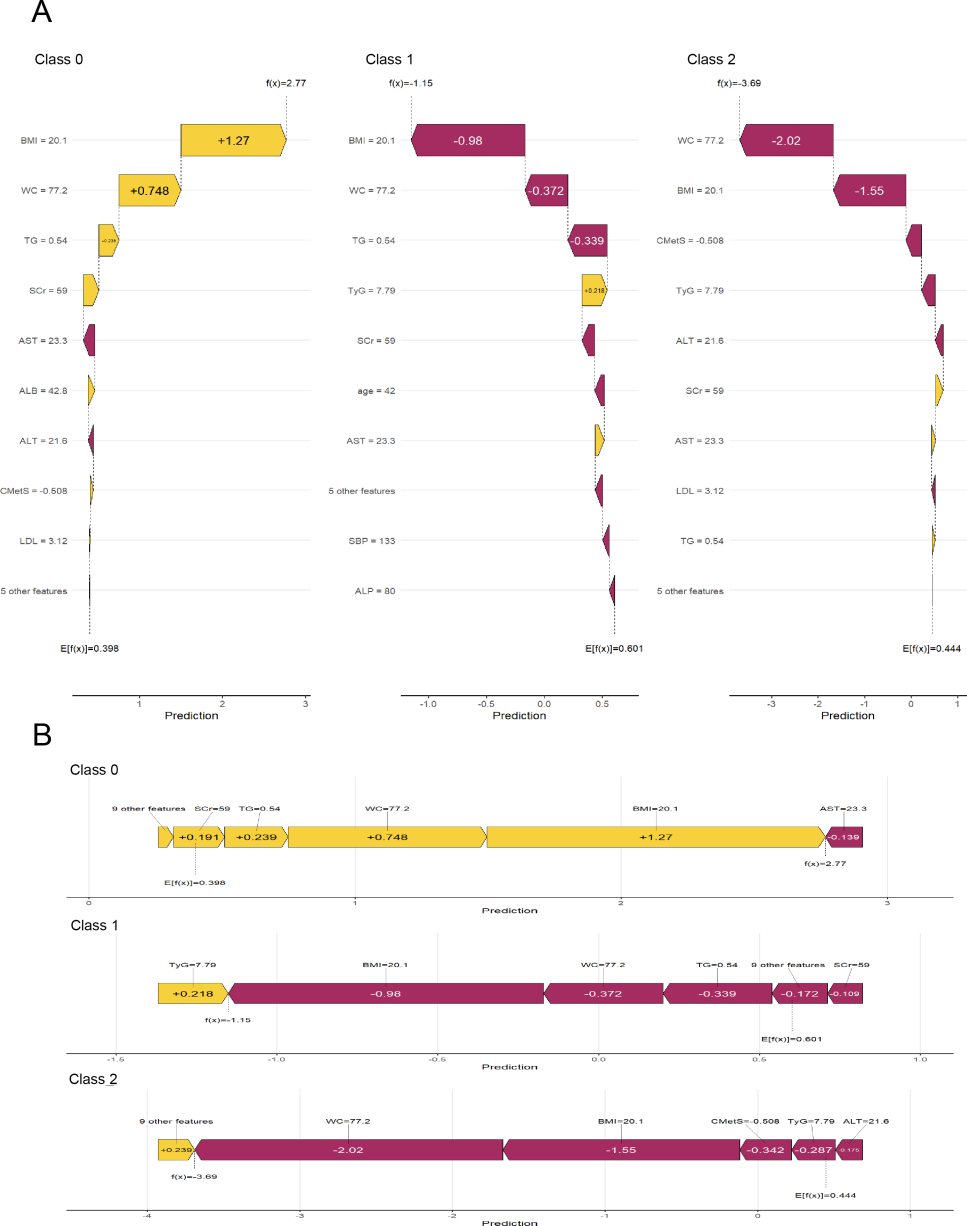
Local interpretability for individual predictions. ALB: albumin; ALP: alkaline phosphatase; ALT: alanine aminotransferase; AST: aspartate aminotransferase; CMetS: continuous metabolic syndrome score; LDL: low-density lipoprotein cholesterol; SBP: systolic blood pressure; SCr: serum creatinine; TG: triglycerides; TyG: triglyceride-glucose index; WC: waist circumference.

Local interpretation clarifies predictions for individual cases. The waterfall plot (Figure 6A) deconstructs how each feature shifts the model output from the baseline for a representative patient. The force plot (Figure 6B) visually maps the cumulative effect of all features in moving the prediction from the base value to the final outcome. These tools translate probabilistic outputs into clinically intelligible terms, allowing verification of biological plausibility and understanding of risk stratification rationale.

### Comprehensive Validation and Comparative Analysis

For independent validation of generalizability, the optimized XGBoost model was applied to an external cohort from the NHANES 2017 to 2020 cycle. Among non-Hispanic Asian participants (RIDRETH3=6) with complete CAP measurements and BMI <28 kg/m², the initial eligible sample comprised 1684 individuals. After excluding participants with any missing values in the variables required by the final model, the external validation cohort consisted of 726 individuals with fully observed data. The cohort exhibited the following distribution across hepatic steatosis grades based on CAP thresholds: none: 321 (44.2%), mild: 207 (28.5%), and moderate to severe: 198 (27.3%). For comparison, our internal cohort (n=215,145) demonstrated a distribution of none: 92,944 (43.2%), mild: 54,121 (25.2%), and moderate to severe: 68,080 (31.6%), indicating similar distribution patterns between cohorts (Multimedia Appendix 9). A comprehensive summary of the baseline demographic and clinical characteristics of this external validation cohort, stratified by steatosis grade, is provided in Multimedia Appendix 10. The model demonstrated robust performance in this independent external validation: macro-average ROC-AUC of 0.874 and micro-average ROC-AUC of 0.887 (Multimedia Appendix 11).

To assess the necessity of a dedicated model, a separate XGBoost model was trained on a general population dataset (including obese individuals) using the same features. When evaluated on the same held-out nonobese test set, the nonobese-specific model showed significantly superior discrimination (macroaverage ROC-AUC: 0.941 vs 0.852; *P*<.001; and microaverage ROC-AUC: 0.946 vs 0.869; *P*<.001), as detailed in Multimedia Appendix 11. This indicates that while a general model retains basic predictive capability, it fails to fully capture the distinct progression patterns in nonobese individuals, highlighting the value of population-specific tools.

To further evaluate clinical utility, a direct comparison was made with the established FLI. As FLI is a binary classifier, a dedicated binary XGBoost model was developed using a diagnostic threshold of CAP >248 dB/m to define “potential hepatic steatosis” (encompassing both mild and moderate-to-severe cases). Correspondingly, for FLI, a threshold of 30 was selected instead of the conventional 60, aligning with the objective of early identification in a nonobese screening context [26]. Both models were evaluated on both the internal test set and the external NHANES validation cohort. The performance of our binary XGBoost model is detailed in Multimedia Appendix 12. As summarized in the comparative table (Multimedia Appendix 13) and illustrated by the receiver operating characteristic (ROC) curve comparison (Multimedia Appendix 14), our model substantially outperformed the FLI. Specifically, it achieved areas under the curve (AUCs) of 0.947 (internal) and 0.892 (external), compared to the FLI’s AUCs of 0.809 and 0.792, respectively. This demonstrates the superior discriminative ability of our ML approach, which leverages a broader set of metabolic and clinical features, for identifying steatosis in nonobese individuals.

### Application of Model

**Figure 7.**
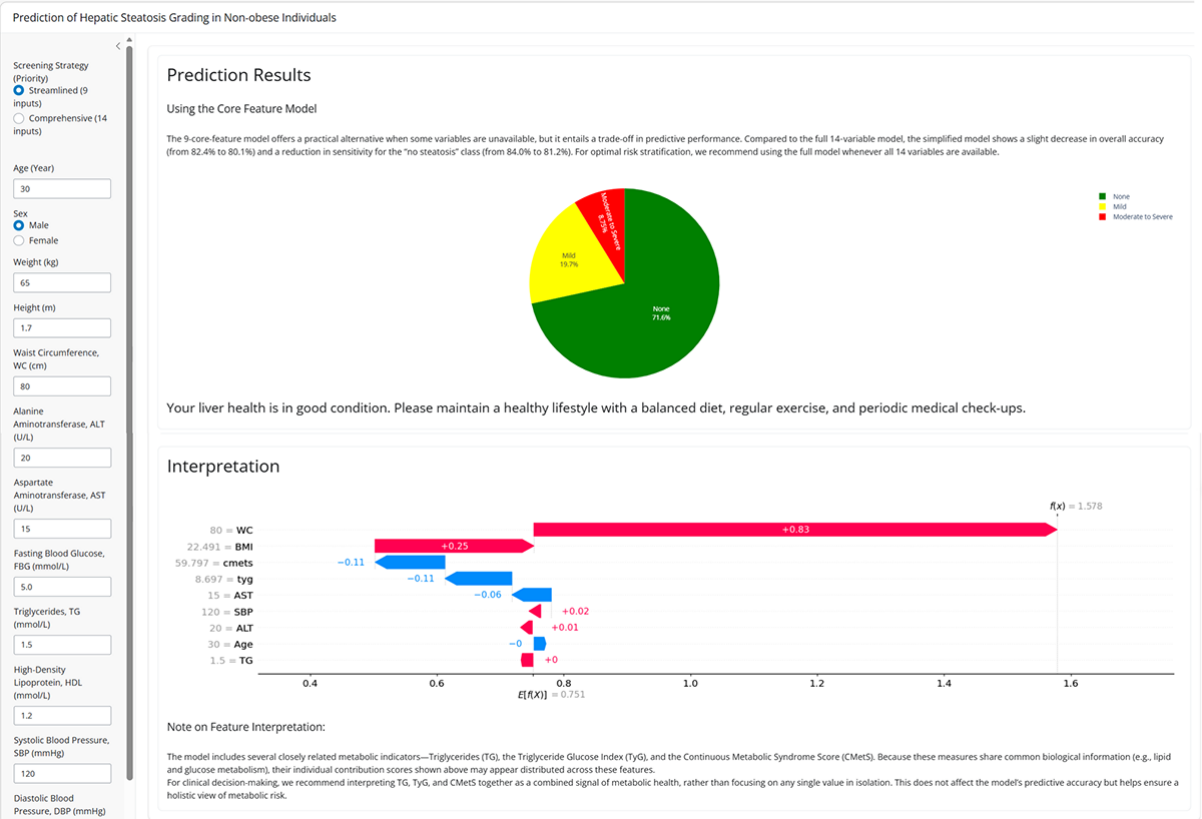
Online prediction platform interface.

To enhance clinical utility, a publicly accessible online prediction platform was developed [27]. By inputting routine indicators (eg, BMI, WC, liver function, and metabolic markers), users obtain real-time, individualized risk stratification (none, mild, or moderate-to-severe probability) and personalized management recommendations. The platform is designed with clinical practicality in mind. It provides predictions via the full 14-variable model when data are complete. To accommodate settings where tests such as albumin, uric acid, ALP, LDL cholesterol, or serum creatinine are unavailable, a simplified “9-core-feature” model has been implemented (see Multimedia Appendix 15 for the 9-core-feature model’s performance). Among these, UA and SCr were excluded primarily due to their lower routine accessibility in primary care settings, as well as their relatively high missing rates in the original cohort (24.01% and 16.04%, respectively); excluding them reduces the uncertainty associated with multiple imputation. Albumin, LDL cholesterol, and ALP were excluded based on their relatively reduced predictive contribution in the full model. It is important to clarify that although the simplified model algorithmically uses 9 derived features, several of these are composite indices—namely, BMI, the TyG, and the continuous metabolic syndrome score—whose computation relies on multiple raw clinical inputs. Consequently, deploying the 9-feature model in practice requires the entry of 12 distinct primary measurements: age, sex, weight, height, waist circumference, ALT, AST, FBG, triglycerides, high-density lipoprotein cholesterol, SBP, and diastolic blood pressure. As detailed in Multimedia Appendix 16, while the macro-average ROC-AUC decreased from 0.941 (full model) to 0.917 (9-core model), the simplified model retains excellent discriminatory performance suitable for initial risk stratification. This dual-model design ensures functionality across varying levels of resource availability. Figure 7 shows an example interface, which allows users to actively select between the core-variable or full-variable model based on data availability. The platform adheres to data privacy standards with a user-friendly interface, bridging the translational gap between artificial intelligence (AI) models and primary health care.

## Discussion

### Principal Findings

This study demonstrates that ML effectively predicts CAP-defined hepatic steatosis severity in nonobese populations, with the XGBoost model achieving superior performance. XGBoost yielded the highest macro-average ROC-AUC (0.941) and PR-AUC (0.890) among all models evaluated. More importantly, it maintained the best precision and sensitivity across all 3 severity classes. It should be noted that CAP is a surrogate marker for histologically confirmed steatosis, with inherent false-positive and false-negative rates compared to liver biopsy. Therefore, the model’s output represents a proxy measure of disease severity, and clinical confirmation may require further diagnostic evaluation.

Nonobese individuals were defined as those with a BMI <28 kg/m². Global epidemiological studies report that 10% to 30% of nonobese individuals have SLD, with prevalence varying geographically (7%‐21% in Europe and America and 3%‐27% in Asia) [4,28,29]. This variability reflects racial differences, inconsistent BMI criteria, and the subjectivity of ultrasound-based diagnosis. The moderate-to-severe steatosis prevalence in our cohort (31.6%) was slightly higher, possibly due to the urban hospital-based sample, as urban populations typically exhibit higher SLD prevalence [3]. Notably, nonobese SLD is associated with significant clinical risk. All-cause mortality in nonobese individuals with SLD (12.1/1000 person-years) is comparable to the overall SLD mortality rate (15.4/1000 person-years) [4], while liver-related mortality in this group may be disproportionately high [30]. These observations underscore that nonobese hepatic steatosis carries outcomes at least comparable to, if not more severe than, those in the general population. The absence of targeted prediction tools contributes to underdiagnosis and delayed intervention. It should be noted that while the cohort was defined as “nonobese” (BMI <28 kg/m²), the median BMI of the moderate-to-severe steatosis group (26.10 kg/m²) falls within the overweight range. Therefore, the predictive performance of the model is primarily representative of and applicable to an “overweight-to-nonobese” population, rather than a predominantly lean population (BMI <24 kg/m²). Nevertheless, our cohort included a substantial proportion of individuals within the normal weight or lean range, constituting approximately 46% of the total sample. The model retained discriminatory ability within this subgroup, indicating its capacity to identify relevant metabolic patterns across the spectrum of nonobese hepatic steatosis, including in leaner individuals.

In this study, we have identified BMI, WC, triglycerides, LDL, ALT, AST, age, SBP, ALP, albumin, UA, SCr, CMetS, and TyG as key factors for hepatic steatosis screening. It should be noted that our training data included mixed steatosis etiologies (eg, metabolic dysfunction–associated SLD and alcohol-related liver disease), and the model predicts the severity of hepatic steatosis without distinguishing its underlying etiology. Consequently, the relevance and contribution of these key predictors might vary across populations with differing prevalent causes. Additionally, key predictors such as albumin (28.6%) and UA (24.0%) had substantial missingness rates; despite rigorous multiple imputation, this remains a limitation as imputation may introduce noise. Several predictors are well-established risk factors for SLD, including obesity-related indicators (BMI and WC) [31], lipid metabolism markers (triglycerides and LDL) [32], and liver enzymes (ALT and AST) [33]. Age is a consistent correlate of SLD risk [34]. Hypertension, reflected by elevated SBP, is independently associated with hepatic steatosis [35]. Albumin may be linked to SLD through oxidative stress and inflammation, which induce posttranslational modifications that impair its antioxidant and anti-inflammatory functions, potentially exacerbating disease [36]. This aligns with findings that the albumin-to-alkaline phosphatase ratio inversely predicts SLD risk in nonobese Chinese cohorts [37]. Our study similarly incorporated ALP and albumin measurements for predicting SLD in nonobese populations, yielding findings consistent with these conclusions. SCr concentration is independently associated with SLD in middle-aged and older Chinese adults, with risk increasing even within the normal range [38], and the UA-to-SCr ratio rises with FLD severity [39]. Compared to the non-SLD group, the adjusted odds ratio for the mild SLD group was 1.147 (95% CI 1.099‐1.196), while the OR for the moderate-to-severe SLD group increased to 1.275 (95% CI 1.212‐1.341). Interestingly, robust studies have revealed a strong association between SLD and CKD [40], regardless of underlying confounding conditions, such as obesity, hypertension, and type 2 diabetes. The TyG index, calculated from FBG and triglycerides, has substantial evidence demonstrating its positive correlation with the severity of insulin resistance in various metabolic diseases [41]. Insulin resistance is also a shared characteristic of SLD across different severity levels. A study from China indicated that the TyG index can serve as a predictor for SLD [42], with an ROC-AUC reaching 0.78. This study further confirms the significance of the TyG index in predicting SLD. CMetS, a composite metric initially developed for cardiovascular risk stratification [25], emerged as an independent predictor here. By integrating several key metabolic components into a single measure, CMetS provides a more informative and holistic summary of metabolic dysfunction, capturing the multifaceted pathophysiological patterns associated with hepatic steatosis severity in this nonobese population. For external validation, the Chinese-derived CMetS formula was applied directly to the NHANES non-Hispanic Asian participants without recalibration. We acknowledge the potential limitation of this approach due to possible differences in metabolic baselines. However, the restricted non-Hispanic Asian subgroup shares phenotypic similarities with the derivation cohort, making this a reasonable test of transferability. Crucially, the model maintained robust performance (macro-average ROC-AUC=0.874), supporting the score’s utility in these metabolically similar groups. An important consideration when interpreting the SHAP results is the multicollinearity among key metabolic predictors—triglycerides, TyG, and CMetS. Although this does not affect the model’s predictive accuracy, it can lead to distributed attribution of shared metabolic risk across these correlated features in SHAP analysis, which may cause clinicians to underestimate the specific contribution of lipid levels such as triglycerides. Therefore, clinicians should interpret the SHAP contributions of triglycerides, TyG, and CMetS collectively as an integrated signal of metabolic dysfunction, rather than in isolation.

The model’s interpretability and accessibility through an online platform facilitate its potential integration as a pre-screening and triage tool in primary care. During routine checkups, practitioners can input readily available parameters to obtain immediate 3-tiered risk stratification. This output supports differentiated management: patients stratified as “none” may require routine follow-up; those with “mild” steatosis can be targeted for lifestyle counseling and monitoring; and those with “moderate-to-severe” disease can be prioritized for confirmatory testing (eg, CAP) or specialist referral. It should be noted that the model’s precision for the “mild” category is relatively limited (test set precision: 0.731 and sensitivity: 0.783). Therefore, we recommend that this classification—and cases with predicted probabilities near any diagnostic threshold—be interpreted cautiously in conjunction with the full clinical context and may warrant additional assessment. To address the practical challenge of acquiring the full 14-variable panel in some settings, we provide a validated simplified model based on 9 core variables, offering a pragmatic alternative for initial risk stratification. While the simplified model retains acceptable discriminatory performance suitable for initial risk stratification (macro-average ROC-AUC decreasing from 0.941 to 0.917), it entails a trade-off, with overall accuracy declining from 82.4% to 80.1% and sensitivity for the “no steatosis” class decreasing from 84.0% to 81.2%. Clinicians should be aware that this parsimonious version may underestimate the probability of mild steatosis, potentially leading to a higher false-negative rate in low-risk classifications. Notably, manual documentation errors often limit the application of comprehensive prediction models. Ambient AI scribes can enhance electronic health record completeness and accuracy by automatically capturing clinical encounters [43]. Integrating such tools into routine practice could facilitate consistent predictor collection. This, in turn, would allow broader deployment of our full 14-variable model and improve its real-world performance. This convergence of AI-enhanced data capture and ML-based risk stratification represents a valuable direction for future research and clinical implementation.

### Comparison With Prior Work

Prior studies have extensively validated ML for predicting SLD. Chen et al demonstrated an XGBoost model outperformed the FLI (ROC-AUC 0.882) for identifying ultrasound-defined moderate-to-severe fatty liver [18]. Subsequent work confirmed the robust performance of models using routine clinical indicators in broader populations (AUC 0.81‐0.89) [19-21]. These studies established a foundation for data-driven prediction but were primarily designed for general or obese populations and relied on ultrasound for binary classification, limiting nuanced risk stratification for nonobese individuals. A notable advance by Su et al [22] focused specifically on lean individuals (BMI <23 kg/m²), achieving an ROC-AUC of 0.885 with a neural network, highlighting the value of population-specific modeling. However, their approach remained a binary classifier based on ultrasound, which has limited sensitivity for mild steatosis. Other advances have integrated multiomics data; for example, Oh et al [44] identified a cross-ethnic gut microbiome signature for predicting cirrhosis (AUC up to 0.91). While offering high performance and mechanistic insight, such approaches rely on specialized assays (eg, metagenomic sequencing), posing challenges for routine, large-scale screening. This study extends prior work through several focused modifications: (1) using the CAP as a quantitative, more sensitive standard than ultrasound; (2) specifically developing the model for a nonobese cohort (BMI <28 kg/m²), with direct validation showing superiority over a general population model (macro-average ROC-AUC 0.941 vs 0.852; *P*<.001); (3) implementing multiclass severity prediction for finer risk stratification; and (4) using only routinely available variables deployed via an interpretable online tool to enhance clinical utility.

The translation of predictive models into clinical practice requires careful consideration of the uncertainties that may affect their reliability. These uncertainties are primarily 3-fold: external uncertainty, relating to the model’s generalizability across populations with differing data distributions; model uncertainty, encompassing both parametric instability and the structural limitations of the chosen algorithm in capturing complex disease pathophysiology; and missing data uncertainty, which arises from incomplete observations. In this study, multiple imputation was applied to the training set (5 datasets), while the test set was imputed only once to reflect real-world deployment. The ensemble of 5 models propagates imputation uncertainty from the training stage. However, a limitation is that single imputation of the test set does not quantify the uncertainty inherent in its own missing values. Novel modeling architectures—such as deep self-supervised learning with advanced feature selection for learning robust representations from unlabeled data [45], and frameworks integrating automated feature engineering with dimensionality reduction to handle data imperfections [46]—aim to develop more reliable and adaptable tools for multidimensional health risk stratification.

In summary, an interpretable XGBoost model was established to predict CAP-defined hepatic steatosis severity in nonobese individuals using routinely available data. By adopting CAP as a quantitative standard, the model enables multiclass risk stratification. Its interpretability, validated generalizability, and deployment as an accessible online platform support its potential integration into clinical workflows for early identification and management in this population.

### Limitations

This study has several limitations. First, generalizability may be limited as the data came predominantly from urban hospitals in Eastern China, lacking rural and diverse ethnic representation, despite robust performance in an external cohort. Additionally, evaluating the model exclusively on participants with complete laboratory records in the external validation may introduce selection bias, as individuals with missing data could differ systematically from those retained. Second, although we compared and selected the best-performing model among 6 candidate algorithms, alternative or more complex architectures may offer superior capabilities for explicit uncertainty quantification. Third, the ongoing debate over unified CAP diagnostic thresholds could affect the consistency of steatosis grading. Finally, the training data included mixed steatosis etiologies, and the model does not distinguish underlying causes. Future validation using larger, more diverse external cohorts with prospectively collected etiological data is needed to further confirm the stability of our performance estimates and enhance clinical utility.

### Conclusions

The interpretable XGBoost model was established for predicting CAP-defined hepatic steatosis severity in nonobese populations based on routinely available clinical and laboratory data in this study. This model demonstrated robust predictive performance in both the internal test set and an external validation cohort. To promote practical application, the model as an openly accessible online prediction platform was used.

## Supplementary material

10.2196/82529 Multimedia Appendix 1 Data missingness pattern for all collected variables in the final study cohort (n=215,145).

10.2196/82529 Multimedia Appendix 2 Distribution of the 6 most influential predictors across hepatic steatosis severity categories.

10.2196/82529 Multimedia Appendix 3 Baseline characteristics of participants in the training and test set.

10.2196/82529 Multimedia Appendix 4 Density plots comparing pre- and post-imputation distributions for key continuous predictors in the training set.

10.2196/82529 Multimedia Appendix 5 Feature selection: Least Absolute Shrinkage and Selection Operator regression and Recursive Feature Elimination based on Random Forest algorithms.

10.2196/82529 Multimedia Appendix 6 Performance of 5 alternative machine learning models.

10.2196/82529 Multimedia Appendix 7 Performance of each algorithm across the 5 imputed datasets.

10.2196/82529 Multimedia Appendix 8 The optimal hyperparameters of each algorithm.

10.2196/82529 Multimedia Appendix 9 Sample size distribution by hepatic steatosis grade in the internal cohort and external National Health and Nutrition Examination Survey validation cohort.

10.2196/82529 Multimedia Appendix 10 Baseline characteristics of the National Health and Nutrition Examination Survey external validation cohort.

10.2196/82529 Multimedia Appendix 11 External validation and model comparison using receiver operating characteristic (ROC) curves.

10.2196/82529 Multimedia Appendix 12 Performance of the binary XGBoost (Extreme Gradient Boosting) model for hepatic steatosis detection.

10.2196/82529 Multimedia Appendix 13 Performance comparison between the binary XGBoost (Extreme Gradient Boosting) model and Fatty Liver Index for hepatic steatosis detection.

10.2196/82529 Multimedia Appendix 14 Comparison of receiver operating characteristic curves between the binary XGBoost (Extreme Gradient Boosting) model and Fatty Liver Index (FLI) for hepatic steatosis detection.

10.2196/82529 Multimedia Appendix 15 Performance of the 9-core-feature XGBoost (Extreme Gradient Boosting) model on the test set.

10.2196/82529 Multimedia Appendix 16 Performance comparison between 14-feature and 9-core-feature XGBoost (Extreme Gradient Boosting) models for nonobese hepatic steatosis severity prediction.

## Abbreviations

AI: artificial intelligence
ALP: alkaline phosphatase
ALT: alanine aminotransferase
AST: aspartate aminotransferase
AUC: area under the curve
CAP: controlled attenuation parameter
CMetS: continuous metabolic syndrome score
FBG: fasting blood glucose
FLD: fatty liver disease
FLI: Fatty Liver Index
HDL: high-density lipoprotein
KNN: k-nearest neighbors
LASSO: Least Absolute Shrinkage and Selection Operator
LDL: low-density lipoprotein
MASLD: metabolic dysfunction–associated steatotic liver disease
ML: machine learning
MLP: multilayer perceptron
NB: naive Bayes
NHANES: National Health and Nutrition Examination Survey
PR: precision-recall
RF: random forest
RFE-RF: Recursive Feature Elimination based on Random Forest
ROC: receiver operating characteristic
ROC-AUC: area under the receiver operating characteristic curve
SBP: systolic blood pressure
SCr: serum creatinine
SHAP: Shapley Additive Explanations
SLD: steatotic liver disease
SVM: support vector machine
TyG: triglyceride-glucose index
UA: uric acid
WC: waist circumference
XGBoost: Extreme Gradient Boosting

## References

[1] Rinella ME, Neuschwander-Tetri BA, Siddiqui MS (2023). AASLD practice guidance on the clinical assessment and management of nonalcoholic fatty liver disease. Hepatology.

[2] Lou TW, Yang RX, Fan JG (2024). The global burden of fatty liver disease: the major impact of China. Hepatobiliary Surg Nutr.

[3] Browning JD, Szczepaniak LS, Dobbins R (2004). Prevalence of hepatic steatosis in an urban population in the United States: impact of ethnicity. Hepatology.

[4] Ye Q, Zou B, Yeo YH (2020). Global prevalence, incidence, and outcomes of non-obese or lean non-alcoholic fatty liver disease: a systematic review and meta-analysis. Lancet Gastroenterol Hepatol.

[5] Hagström H, Nasr P, Ekstedt M (2018). Risk for development of severe liver disease in lean patients with nonalcoholic fatty liver disease: a long-term follow-up study. Hepatol Commun.

[6] Wang AY, Dhaliwal J, Mouzaki M (2019). Lean non-alcoholic fatty liver disease. Clin Nutr.

[7] VanWagner LB, Armstrong MJ (2018). Lean NAFLD: a not so benign condition?. Hepatol Commun.

[8] Kim D, Kim WR (2017). Nonobese fatty liver disease. Clin Gastroenterol Hepatol.

[9] Paige JS, Bernstein GS, Heba E (2017). A pilot comparative study of quantitative ultrasound, conventional ultrasound, and MRI for predicting histology-determined steatosis grade in adult nonalcoholic fatty liver disease. AJR Am J Roentgenol.

[10] Li Y, Wang X, Zhang J, Zhang S, Jiao J (2022). Applications of artificial intelligence (AI) in researches on non-alcoholic fatty liver disease(NAFLD): a systematic review. Rev Endocr Metab Disord.

[11] Castera L, Friedrich-Rust M, Loomba R (2019). Noninvasive assessment of liver disease in patients with nonalcoholic fatty liver disease. Gastroenterology.

[12] Karlas T, Petroff D, Sasso M (2017). Individual patient data meta-analysis of controlled attenuation parameter (CAP) technology for assessing steatosis. J Hepatol.

[13] Eddowes PJ, Sasso M, Allison M (2019). Accuracy of FibroScan controlled attenuation parameter and liver stiffness measurement in assessing steatosis and fibrosis in patients with nonalcoholic fatty liver disease. Gastroenterology.

[14] Sasso M, Beaugrand M, de Ledinghen V (2010). Controlled attenuation parameter (CAP): a novel VCTE. Ultrasound Med Biol.

[15] Blond E, Disse E, Cuerq C (2017). EASL-EASD-EASO clinical practice guidelines for the management of non-alcoholic fatty liver disease in severely obese people: do they lead to over-referral?. Diabetologia.

[16] Pouwels S, Sakran N, Graham Y (2022). Non-alcoholic fatty liver disease (NAFLD): a review of pathophysiology, clinical management and effects of weight loss. BMC Endocr Disord.

[17] Dietrich P, Hellerbrand C (2014). Non-alcoholic fatty liver disease, obesity and the metabolic syndrome. Best Pract Res Clin Gastroenterol.

[18] Chen YY, Lin CY, Yen HH (2022). Machine-learning algorithm for predicting fatty liver disease in a Taiwanese population. J Pers Med.

[19] Deng J, Ji W, Liu H (2024). Development and validation of a machine learning-based framework for assessing metabolic-associated fatty liver disease risk. BMC Public Health.

[20] Weng S, Hu D, Chen J, Yang Y, Peng D (2023). Prediction of fatty liver disease in a Chinese population using machine-learning algorithms. Diagnostics (Basel).

[21] Huang G, Jin Q, Mao Y (2023). Predicting the 5-year risk of nonalcoholic fatty liver disease using machine learning models: prospective cohort study. J Med Internet Res.

[22] Su PY, Chen YY, Lin CY, Su WW, Huang SP, Yen HH (2023). Comparison of machine learning models and the fatty liver index in predicting lean fatty liver. Diagnostics (Basel).

[23] Bei‐Fan Z, the Cooperative Meta‐analysis Group of Working Group on Obesity in China (2002). Predictive values of body mass index and waist circumference for risk factors of certain related diseases in Chinese adults: study on optimal cut‐off points of body mass index and waist circumference in Chinese adults. Asia Pac J Clin Nutr.

[24] (2004). Appropriate body-mass index for Asian populations and its implications for policy and intervention strategies. The Lancet.

[25] Yang S, Yu B, Yu W (2023). Development and validation of an age-sex-ethnicity-specific metabolic syndrome score in the Chinese adults. Nat Commun.

[26] Bedogni G, Bellentani S, Miglioli L (2006). The fatty liver index: a simple and accurate predictor of hepatic steatosis in the general population. BMC Gastroenterol.

[27] Hepatic steatosis grading prediction platform.

[28] Kim D, Kim W, Joo SK (2019). Predictors of nonalcoholic steatohepatitis and significant fibrosis in non-obese nonalcoholic fatty liver disease. Liver Int.

[29] Shi Y, Wang Q, Sun Y (2020). The prevalence of lean/nonobese nonalcoholic fatty liver disease: a systematic review and meta-analysis. J Clin Gastroenterol.

[30] Lonardo A, Byrne CD, Caldwell SH, Cortez-Pinto H, Targher G (2016). Global epidemiology of nonalcoholic fatty liver disease: meta-analytic assessment of prevalence, incidence, and outcomes. Hepatology.

[31] Ayada I, van Kleef LA, Alferink LJM, Li P, de Knegt RJ, Pan Q (2022). Systematically comparing epidemiological and clinical features of MAFLD and NAFLD by meta-analysis: focusing on the non-overlap groups. Liver Int.

[32] Kyhl LK, Nordestgaard BG, Tybjærg-Hansen A, Nielsen SF (2023). High fat in blood and body and increased risk of clinically diagnosed non-alcoholic fatty liver disease in 105,981 individuals. Atherosclerosis.

[33] Xuan Y, Wu D, Zhang Q, Yu Z, Yu J, Zhou D (2024). Elevated ALT/AST ratio as a marker for NAFLD risk and severity: insights from a cross-sectional analysis in the United States. Front Endocrinol.

[34] Peng H, Pan L, Ran S (2023). Prediction of MAFLD and NAFLD using different screening indexes: a cross-sectional study in U.S. adults. Front Endocrinol.

[35] Yuan M, He J, Hu X (2024). Hypertension and NAFLD risk: insights from the NHANES 2017-2018 and Mendelian randomization analyses. Chin Med J (Engl).

[36] Wu N, Liu T, Tian M (2024). Albumin, an interesting and functionally diverse protein, varies from “native” to “effective” (Review). Mol Med Rep.

[37] Sheng G, Peng N, Hu C, Zhong L, Zhong M, Zou Y (2021). The albumin-to-alkaline phosphatase ratio as an independent predictor of future non-alcoholic fatty liver disease in a 5-year longitudinal cohort study of a non-obese Chinese population. Lipids Health Dis.

[38] Niu Y, Zhang W, Zhang H (2022). Serum creatinine levels and risk of nonalcohol fatty liver disease in a middle-aged and older Chinese population: a cross-sectional analysis. Diabetes Metab Res Rev.

[39] Choi J, Joe H, Oh JE, Cho YJ, Shin HS, Heo NH (2023). The correlation between NAFLD and serum uric acid to serum creatinine ratio. PLoS ONE.

[40] Byrne CD, Targher G (2020). NAFLD as a driver of chronic kidney disease. J Hepatol.

[41] Wei X, Min Y, Song G, Ye X, Liu L (2024). Association between triglyceride-glucose related indices with the all-cause and cause-specific mortality among the population with metabolic syndrome. Cardiovasc Diabetol.

[42] Zhang S, Du T, Zhang J (2017). The triglyceride and glucose index (TyG) is an effective biomarker to identify nonalcoholic fatty liver disease. Lipids Health Dis.

[43] Leung TI, Coristine AJ, Benis A (2025). AI scribes in health care: balancing transformative potential with responsible integration. JMIR Med Inform.

[44] Oh TG, Kim SM, Caussy C (2020). A universal gut-microbiome-derived signature predicts cirrhosis. Cell Metab.

[45] Tutsoy O, Koç GG (2024). Deep self-supervised machine learning algorithms with a novel feature elimination and selection approaches for blood test-based multi-dimensional health risks classification. BMC Bioinformatics.

[46] Tutsoy O, Sumbul HE (2024). A novel deep machine learning algorithm with dimensionality and size reduction approaches for feature elimination: thyroid cancer diagnoses with randomly missing data. Brief Bioinform.

[47] NHANES questionnaires, datasets, and related documentation. CDC.

[48] GitHub repository for non-obese hepatic steatosis prediction code. GitHub.

