# Supplementary material for: Hepatic Steatosis Severity Prediction in Nonobese Individuals: Machine Learning Model Development and Validation
Source: J Med Internet Res. 2026 Jun 19;28:e82529. doi: 10.2196/82529 (PMC13282044; doi:10.2196/82529)
Supplement: Multimedia Appendix 1 [file jmir-v28-e82529-s001.docx]

Multimedia Appendix 1. Data missingness pattern for all collected variables in the final study cohort (number of participants, n=215,145).


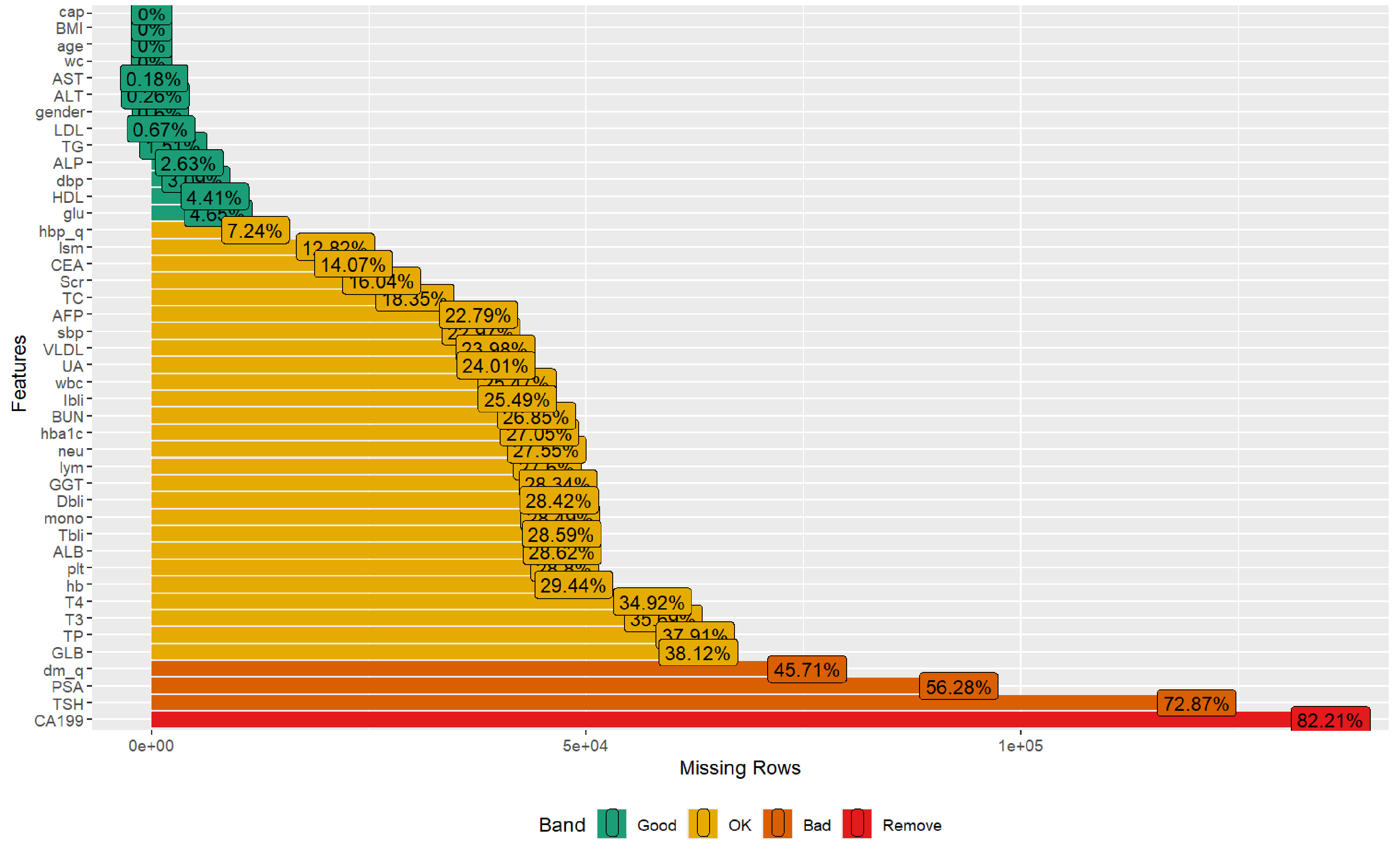

This figure illustrates the extent of missing data across candidate predictor variables in the final analytic cohort. Variables exceeding 30% missingness were excluded from further analysis.
