# Supplementary material for: Hepatic Steatosis Severity Prediction in Nonobese Individuals: Machine Learning Model Development and Validation
Source: J Med Internet Res. 2026 Jun 19;28:e82529. doi: 10.2196/82529 (PMC13282044; doi:10.2196/82529)
Supplement: Multimedia Appendix 2 [file jmir-v28-e82529-s002.docx]

Multimedia Appendix 2. Distribution of the six most influential predictors across hepatic steatosis severity categories.


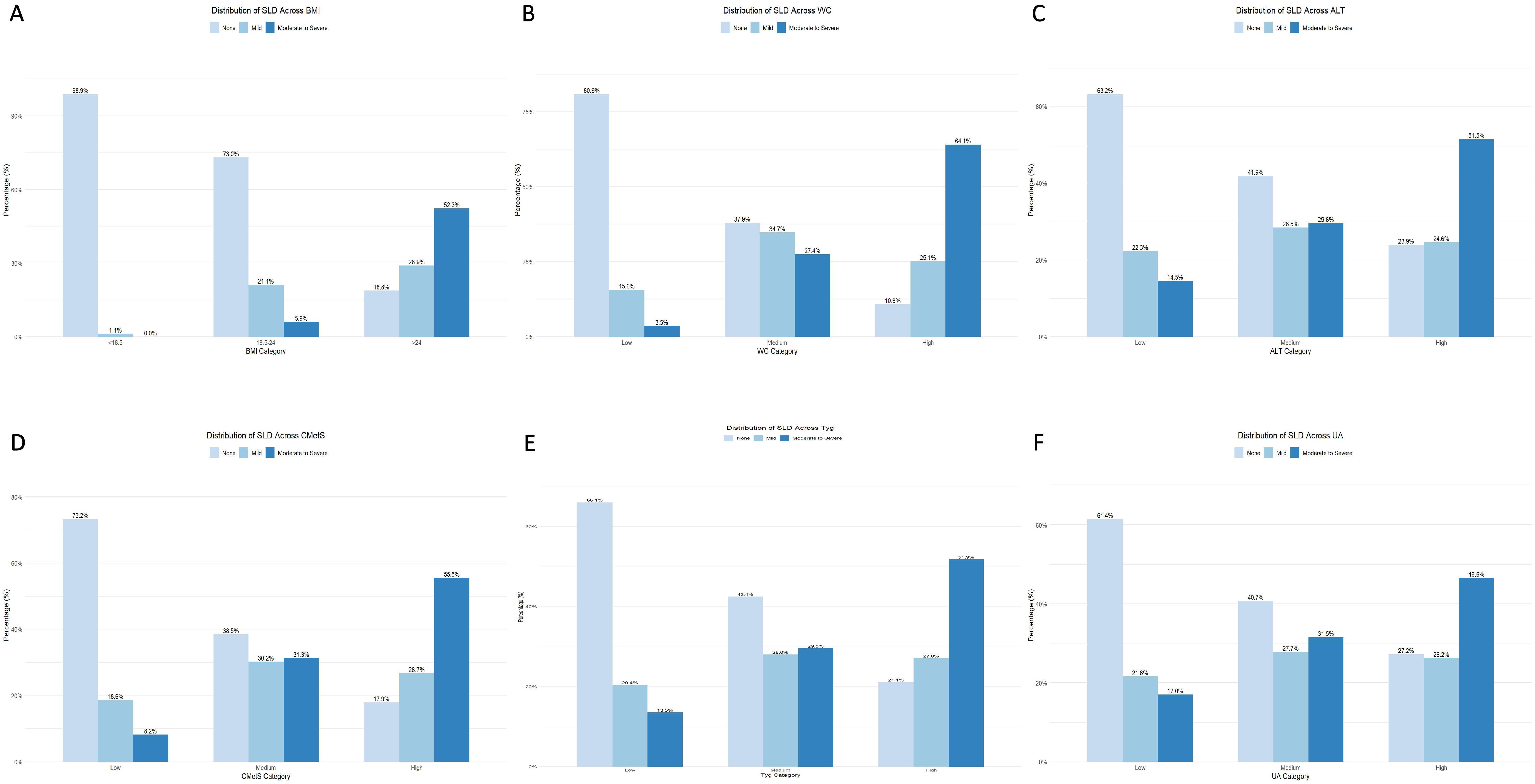

This figure presents the distribution of the six most influential predictors (such as BMI, waist circumference, and ALT) across the three hepatic steatosis severity groups (none, mild, moderate-to-severe), illustrating their trends and variations.
