# Supplementary material for: Hepatic Steatosis Severity Prediction in Nonobese Individuals: Machine Learning Model Development and Validation
Source: J Med Internet Res. 2026 Jun 19;28:e82529. doi: 10.2196/82529 (PMC13282044; doi:10.2196/82529)
Supplement: Multimedia Appendix 3 [file jmir-v28-e82529-s003.doc]

| Multimedia Appendix 3. Baseline characteristics of participants in the training and test set. | | | | |
| --- | --- | --- | --- | --- |
| Variables | Total (n = 215145) | Training set (n = 150601) | Test set (n = 64544) | *P*^ah^ value |
| Age (year) | 44.00 (34.00, 55.00) | 44.00 (34.00, 55.00) | 44.00 (34.00, 55.00) | .57 |
| Gender, n (%) |  |  |  | .94 |
| Female | 103979 (48.33) | 72831 (48.36) | 31148 (48.26) |  |
| Male | 109875 (51.07) | 76897 (51.06) | 32978 (51.09) |  |
| Miss | 1291 (0.60) | 873 (0.58) | 418 (0.65) |  |
| CAP^a^ (dB/m) | 251.00 (227.00, 275.00) | 251.00 (227.00, 275.00) | 251.00 (227.00, 275.00) | .98 |
| LSM^b^ (kPa) | 5.80 (4.90, 6.70) | 5.80 (4.90, 6.70) | 5.80 (4.90, 6.70) | .11 |
| Miss | 27582 (12.82) | 19276 (12.8) | 8306 (12.87) |  |
| WC^c^ (cm) | 88.00 (83.00, 92.00) | 88.00 (83.00, 92.00) | 88.00 (83.00, 92.00) | .48 |
| BMI^d^ (kg/m^2^) | 24.30 (22.47, 25.90) | 24.30 (22.47, 25.90) | 24.30 (22.49, 25.90) | .96 |
| SBP^e^ (mmHg) | 124.00 (113.00, 136.00) | 124.00 (113.00, 136.00) | 124.00 (113.00, 136.00) | .36 |
| Miss | 49419 (22.97) | 34578 (22.96) | 14841 (22.99) |  |
| DBP^f^ (mmHg) | 77.00 (70.00, 85.00) | 77.00 (70.00, 85.00) | 77.00 (70.00, 85.00) | .15 |
| Miss | 6648 (3.09) | 4694 (3.12) | 1954 (3.03) |  |
| Lym^g^ (%) | 33.60 (28.70, 38.70) | 33.50 (28.70, 38.70) | 33.60 (28.70, 38.60) | .60 |
| Miss | 59380 (27.6) | 41874 (27.8) | 17506 (27.12) |  |
| PLT^h^ (*10⁹/L) | 224.00 (191.00, 261.00) | 224.00 (190.50, 261.00) | 225.00 (191.00, 262.00) | .17 |
| Miss | 61962 (28.8) | 43697 (29.02) | 18265 (28.3) |  |
| Mono^i^ (%) | 6.50 (5.50, 7.70) | 6.50 (5.50, 7.70) | 6.50 (5.50, 7.70) | .56 |
| Miss | 61295 (28.49) | 43235 (28.71) | 18060 (27.98) |  |
| Neu^j^ (%) | 56.60 (51.20, 61.80) | 56.50 (51.20, 61.90) | 56.60 (51.30, 61.80) | .51 |
| Miss | 59272 (27.55) | 41807 (27.76) | 17465 (27.06) |  |
| WBC^k^ (*10⁹/L) | 5.85 (5.00, 6.84) | 5.85 (5.00, 6.83) | 5.84 (5.00, 6.84) | .94 |
| Miss | 54797 (25.47) | 38651 (25.66) | 16146 (25.02) |  |
| Hb^l^ (g/L) | 148.00 (136.00, 156.00) | 148.00 (136.00, 156.00) | 148.00 (136.00, 156.00) | .17 |
| Miss | 63339 (29.44) | 44674 (29.66) | 18665 (28.92) |  |
| FBG^m^ (mmol/L) | 5.08 (4.76, 5.47) | 5.08 (4.76, 5.47) | 5.07 (4.77, 5.48) | .74 |
| Miss | 10004 (4.65) | 7058 (4.69) | 2946 (4.56) |  |
| TG^n^ (mmol/L) | 1.32 (0.93, 1.92) | 1.32 (0.93, 1.92) | 1.32 (0.93, 1.92) | .81 |
| Miss | 3249 (1.51) | 2294 (1.52) | 955 (1.48) |  |
| TC^o^ (mmol/L) | 4.78 (4.22, 5.39) | 4.78 (4.22, 5.39) | 4.79 (4.23, 5.40) | .12 |
| Miss | 39479 (18.35) | 27853 (18.49) | 11626 (18.01) |  |
| HDL^p^ (mmol/L) | 1.13 (0.96, 1.35) | 1.13 (0.95, 1.35) | 1.13 (0.96, 1.35) | .12 |
| Miss | 9488 (4.41) | 6695 (4.45) | 2793 (4.33) |  |
| LDL^q^ (mmol/L) | 2.84 (2.35, 3.36) | 2.84 (2.35, 3.36) | 2.84 (2.36, 3.36) | .20 |
| Miss | 1441 (0.67) | 1018 (0.68) | 423 (0.66) |  |
| VLDL^r^ (mmol/L) | 0.72 (0.54, 0.95) | 0.72 (0.54, 0.95) | 0.72 (0.54, 0.95) | .31 |
| Miss | 51592 (23.98) | 36401 (24.17) | 15191 (23.54) |  |
| HbA1c^s^ (%) | 5.50 (5.30, 5.80) | 5.50 (5.30, 5.80) | 5.50 (5.30, 5.80) | .09 |
| Miss | 58197 (27.05) | 41056 (27.26) | 17141 (26.56) |  |
| ALT^t^ (U/L) | 21.00 (15.50, 31.00) | 21.00 (15.60, 31.00) | 21.00 (15.40, 31.00) | .61 |
| Miss | 559 (0.26) | 396 (0.26) | 163 (0.25) |  |
| AST^u^ (U/L) | 22.00 (19.00, 26.70) | 22.00 (19.00, 26.80) | 22.00 (19.00, 26.50) | .87 |
| Miss | 387 (0.18) | 274 (0.18) | 113 (0.18) |  |
| GGT^v^ (U/L) | 22.80 (15.00, 36.20) | 22.80 (15.00, 36.10) | 22.70 (15.00, 36.50) | .92 |
| Miss | 60972 (28.34) | 43011 (28.56) | 17961 (27.83) |  |
| ALP^w^ (IU/L) | 72.00 (61.00, 86.00) | 72.00 (61.00, 86.00) | 72.00 (61.00, 86.00) | .47 |
| Miss | 5658 (2.63) | 3995 (2.65) | 1663 (2.58) |  |
| BUN^x^ (mmol/L) | 5.26 (4.49, 6.14) | 5.26 (4.50, 6.15) | 5.25 (4.48, 6.13) | .07 |
| Miss | 57766 (26.85) | 40748 (27.06) | 17018 (26.37) |  |
| SCr^y^ (μmol/L) | 69.00 (60.00, 78.00) | 69.00 (60.00, 78.00) | 69.00 (60.00, 78.00) | .08 |
| Miss | 34509 (16.04) | 24353 (16.17) | 10156 (15.74) |  |
| UA^z^ (μmol/L) | 360.70 (303.00, 419.00) | 361.00 (303.00, 419.20) | 360.10 (302.10, 419.00) | .38 |
| Miss | 51656 (24.01) | 36443 (24.2) | 15213 (23.57) |  |
| AFP^aa^ (ng/mL) | 2.80 (2.00, 3.96) | 2.80 (2.00, 3.96) | 2.80 (2.00, 3.97) | .32 |
| Miss | 49032 (22.79) | 34590 (22.97) | 14442 (22.38) |  |
| CEA^ab^ (ng/mL) | 1.69 (1.18, 2.43) | 1.69 (1.18, 2.43) | 1.70 (1.18, 2.44) | .45 |
| Miss | 30271 (14.07) | 21360 (14.18) | 8911 (13.81) |  |
| ALB^ac^ (g/L) | 46.00 (44.30, 47.70) | 46.00 (44.30, 47.70) | 46.00 (44.30, 47.70) | .96 |
| Miss | 61574 (28.62) | 43439 (28.84) | 18135 (28.1) |  |
| Tbli^ad^ (μmol/L) | 14.30 (11.30, 18.20) | 14.30 (11.30, 18.20) | 14.30 (11.30, 18.20) | .81 |
| Miss | 61510 (28.59) | 43398 (28.82) | 18112 (28.06) |  |
| Dbli^ae^ (μmol/L) | 4.10 (2.90, 5.50) | 4.10 (2.90, 5.50) | 4.10 (2.95, 5.50) | .86 |
| Miss | 61144 (28.42) | 43141 (28.65) | 18003 (27.89) |  |
| Ibli^af^ (μmol/L) | 10.30 (8.00, 13.30) | 10.30 (8.00, 13.30) | 10.30 (8.00, 13.30) | .74 |
| Miss | 54840 (25.49) | 38692 (25.69) | 16148 (25.02) |  |
| HBP^ag^, n(%) |  |  |  | .69 |
| No | 120761 (56.13) | 84593 (56.17) | 36168 (56.04) |  |
| Yes | 78808 (36.63) | 55135 (36.61) | 23673 (36.67) |  |
| Miss | 15576 (7.24) | 10873 (7.22) | 4703 (7.29) |  |
| TyG^ai^ | 8.68 (8.29, 9.10) | 8.68 (8.29, 9.10) | 8.68 (8.29, 9.11) | .66 |
| Miss | 11682 (5.43) | 8223(5.46) | 3459(5.40) |  |
| CMetS^aj^ | 0.33 (-0.10, 0.78) | 0.33 (-0.10, 0.78) | 0.33 (-0.10, 0.79) | .75 |
| Miss | 50925 (23.67) | 35677(23.69) | 15248 (23.62) |  |
| Note: ᵃCAP: controlled attenuation parameter; ᵇLSM: liver stiffness measurement; ᶜWC: waist circumference; ᵈBMI: body mass index; ᵉSBP: systolic blood pressure; ᶠDBP: diastolic blood pressure; ᵍLym: lymphocyte percentage; ʰPLT: platelet count; ⁱMono: monocyte percentage; ʲNeu: neutrophil percentage; ᵏWBC: white blood cell count; ˡHb: hemoglobin; ᵐFBG: fasting blood glucose; ⁿTG: triglycerides; ᵒTC: total cholesterol; ᵖHDL: high-density lipoprotein cholesterol; ᵠLDL: low-density lipoprotein cholesterol; ʳVLDL: very low-density lipoprotein cholesterol; ˢHbA1c: hemoglobin A1c; ᵗALT: alanine aminotransferase; ᵘAST: aspartate aminotransferase; ᵛGGT: gamma-glutamyl transferase; ʷALP: alkaline phosphatase; ˣBUN: blood urea nitrogen; ʸSCr: serum creatinine; ᶻUA: uric acid; ᵃᵃAFP: alpha-fetoprotein; ᵃᵇCEA: carcinoembryonic antigen; ᵃᶜALB: albumin; ᵃᵈTbli: total bilirubin; ᵃᵉDbli: direct bilirubin; ᵃᶠIbli: indirect bilirubin; ᵃᵍHBP: high blood pressure; ^ai^TyG: the triglyceride-glucose index; ^aj^CMetS: the continuous metabolic syndrome score. Continuous variables were characterized as median (IQR), while categorical variables were described in terms of frequency (percentage). P^ah^ values for continuous variables were calculated using the Mann-Whitney U test and those for categorical variables were calculated using Pearson’s chi-squared test. | | | | |
