# Supplementary material for: Hepatic Steatosis Severity Prediction in Nonobese Individuals: Machine Learning Model Development and Validation
Source: J Med Internet Res. 2026 Jun 19;28:e82529. doi: 10.2196/82529 (PMC13282044; doi:10.2196/82529)
Supplement: Multimedia Appendix 4 [file jmir-v28-e82529-s004.docx]

Multimedia Appendix 4. Density plots comparing pre- and post-imputation distributions for key continuous predictors in the training set.


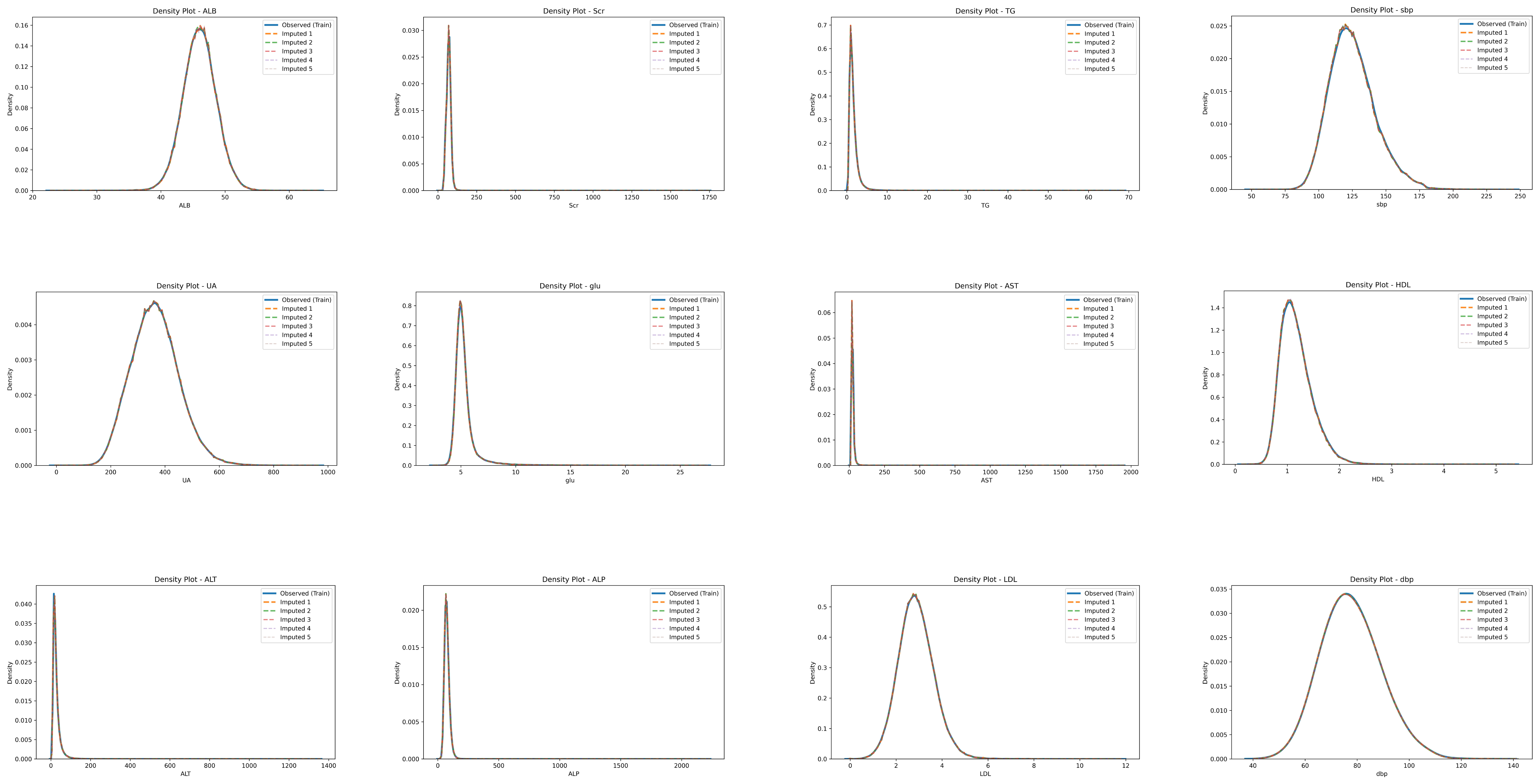


Density plots illustrating the distribution of original observed values and five multiply imputed datasets for 12 key continuous variables in the training set. The close alignment between the observed and imputed density curves across all variables indicates that the multiple imputation procedure successfully preserved the underlying distributional characteristics of each variable, supporting the validity of the imputation model.
