# Supplementary material for: Hepatic Steatosis Severity Prediction in Nonobese Individuals: Machine Learning Model Development and Validation
Source: J Med Internet Res. 2026 Jun 19;28:e82529. doi: 10.2196/82529 (PMC13282044; doi:10.2196/82529)
Supplement: Multimedia Appendix 5 [file jmir-v28-e82529-s005.docx]

Multimedia Appendix 5. Feature Selection: LASSO Regression and RFE-RF Algorithms.

After multiple imputation of the training set (5 datasets, mice R package with random forest imputation), LASSO regression and RFE‑RF were applied independently to each imputed dataset. In each dataset, the predictors selected by both methods (intersection) were retained. Finally, only those features appearing in at least three of the five imputed datasets were chosen as the final set of predictors for model development. This approach ensures robustness and consistency of the selected features across the uncertainty introduced by missing data imputation.


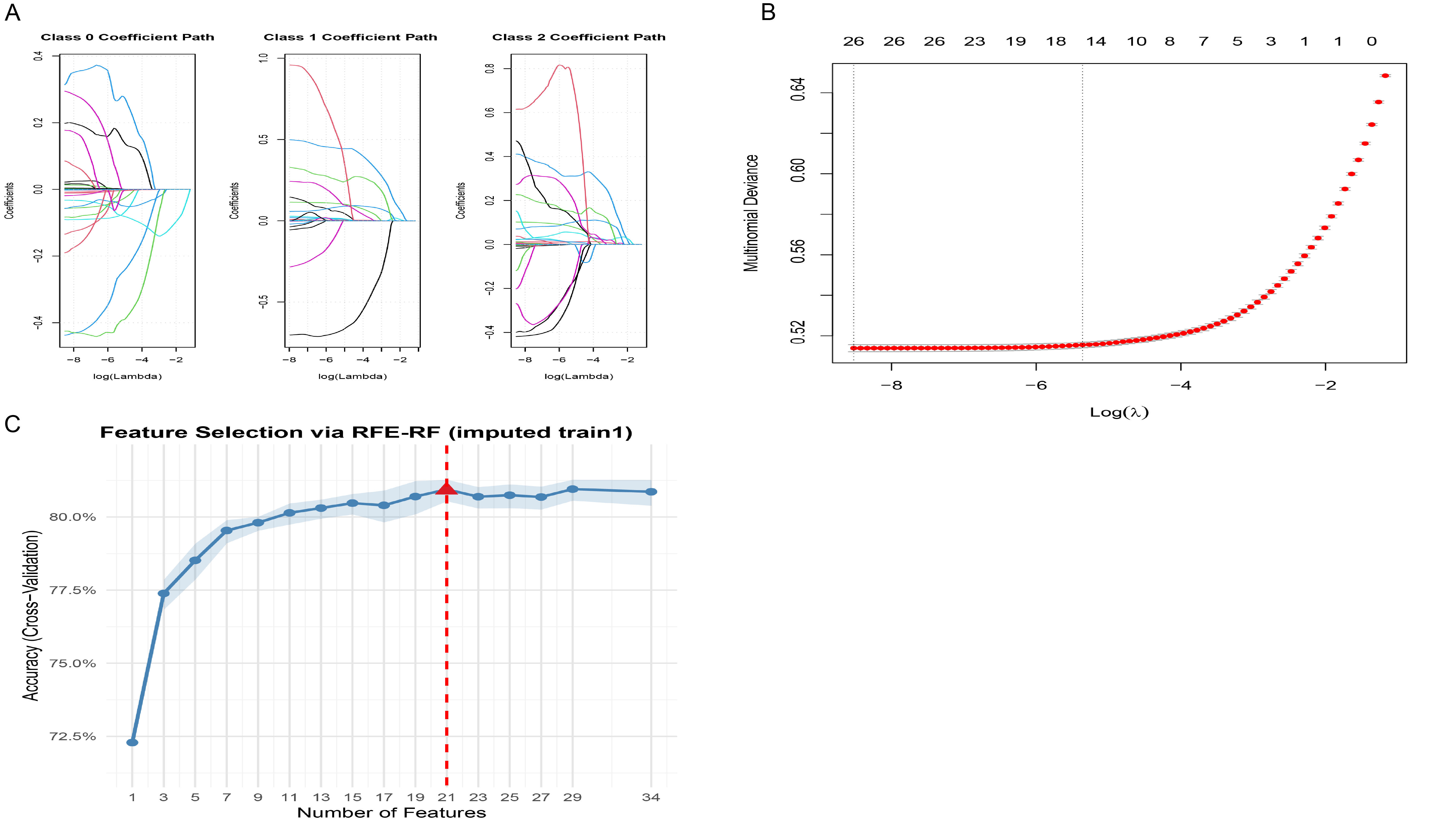


This figure demonstrates the application of Least Absolute Shrinkage and Selection Operator (LASSO) regression and Recursive Feature Elimination based on Random Forest importance (RFE-RF) in the imputed dataset 1 for identifying key predictors associated with hepatic steatosis severity. (A) LASSO regression coefficient profile: Coefficients of candidate variables shrink to zero as the regularization parameter (λ) increases, indicating variable elimination. (B) LASSO cross-validation curve: Binomial deviance is plotted against log(λ), with the vertical dashed lines marking the optimal λ (minimum deviance) and the more conservative λ within 1 standard deviation of the minimum. (C) RFE-RF feature selection curve: Model accuracy (on the validation set) is plotted against the number of selected features, showing the trend of performance change as features are recursively eliminated. LASSO: Least Absolute Shrinkage and Selection Operator, RFE-RF: Recursive Feature Elimination based on Random Forest importance.


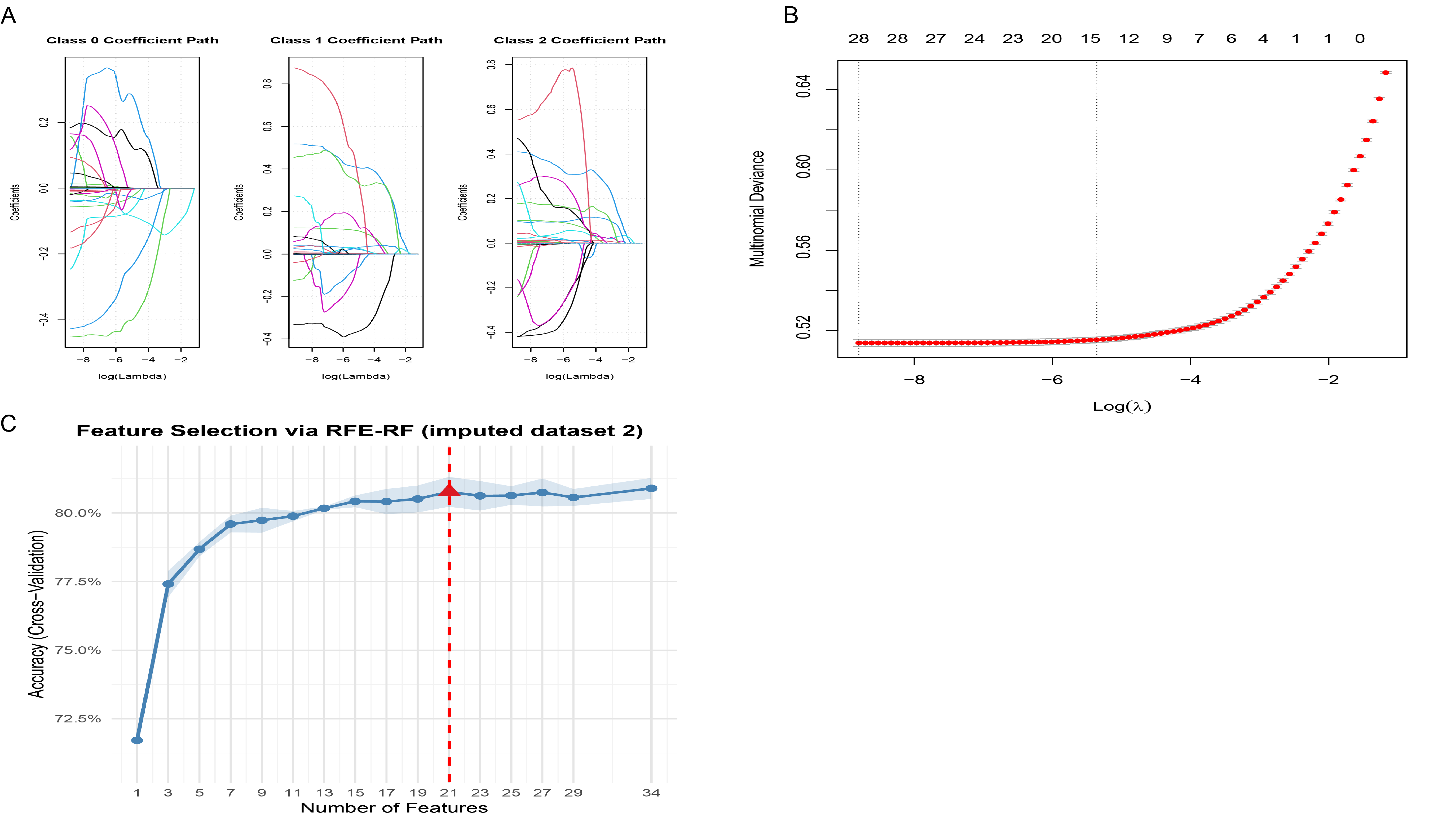


This figure demonstrates the application of Least Absolute Shrinkage and Selection Operator (LASSO) regression and Recursive Feature Elimination based on Random Forest importance (RFE-RF) in the imputed dataset 2 for identifying key predictors associated with hepatic steatosis severity. (A) LASSO regression coefficient profile: Coefficients of candidate variables shrink to zero as the regularization parameter (λ) increases, indicating variable elimination. (B) LASSO cross-validation curve: Binomial deviance is plotted against log(λ), with the vertical dashed lines marking the optimal λ (minimum deviance) and the more conservative λ within 1 standard deviation of the minimum. (C) RFE-RF feature selection curve: Model accuracy (on the validation set) is plotted against the number of selected features, showing the trend of performance change as features are recursively eliminated. LASSO: Least Absolute Shrinkage and Selection Operator, RFE-RF: Recursive Feature Elimination based on Random Forest importance.


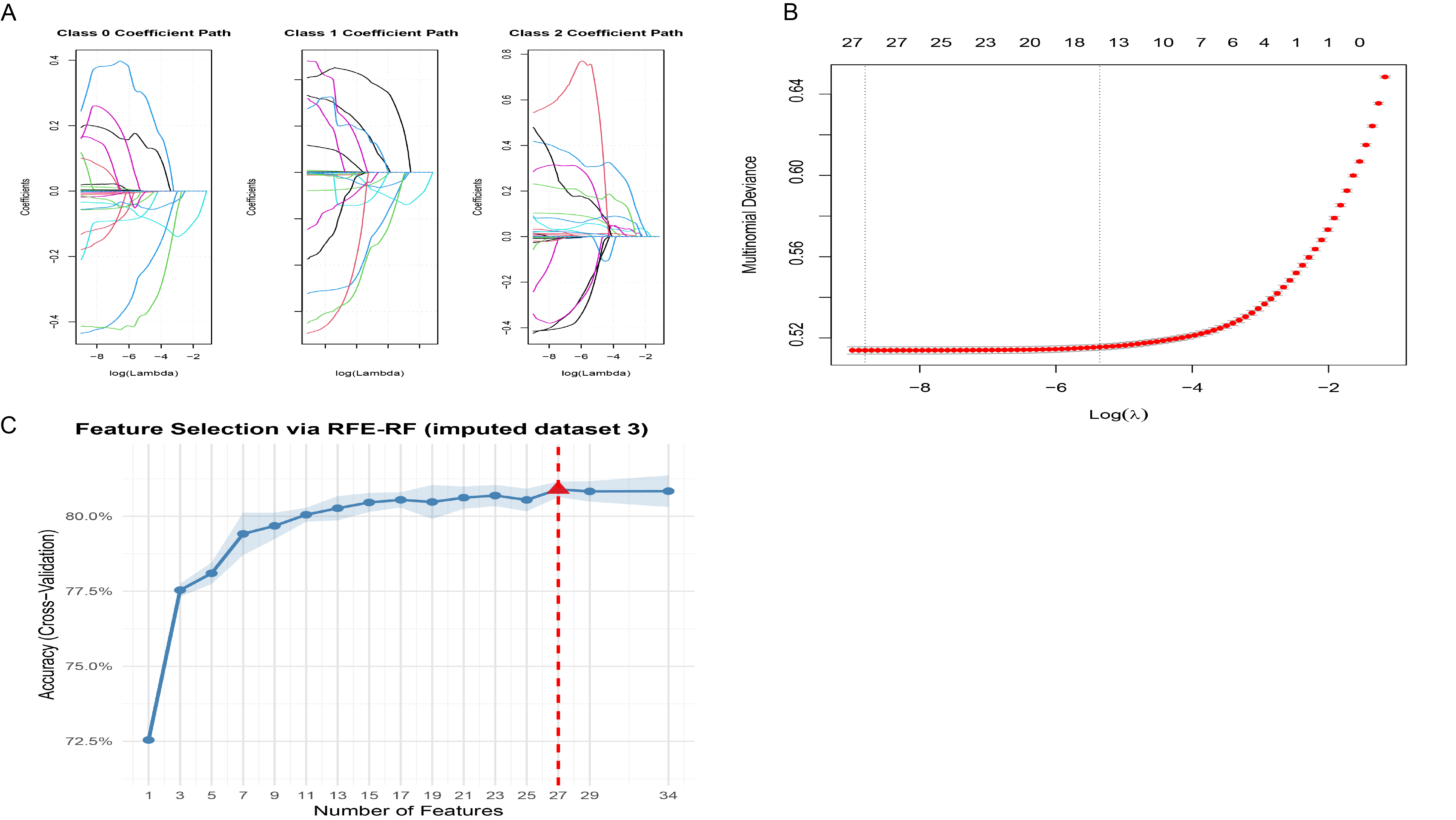
 This figure demonstrates the application of Least Absolute Shrinkage and Selection Operator (LASSO) regression and Recursive Feature Elimination based on Random Forest importance (RFE-RF) in the imputed dataset 3 for identifying key predictors associated with hepatic steatosis severity. (A) LASSO regression coefficient profile: Coefficients of candidate variables shrink to zero as the regularization parameter (λ) increases, indicating variable elimination. (B) LASSO cross-validation curve: Binomial deviance is plotted against log(λ), with the vertical dashed lines marking the optimal λ (minimum deviance) and the more conservative λ within 1 standard deviation of the minimum. (C) RFE-RF feature selection curve: Model accuracy (on the validation set) is plotted against the number of selected features, showing the trend of performance change as features are recursively eliminated. LASSO: Least Absolute Shrinkage and Selection Operator, RFE-RF: Recursive Feature Elimination based on Random Forest importance.


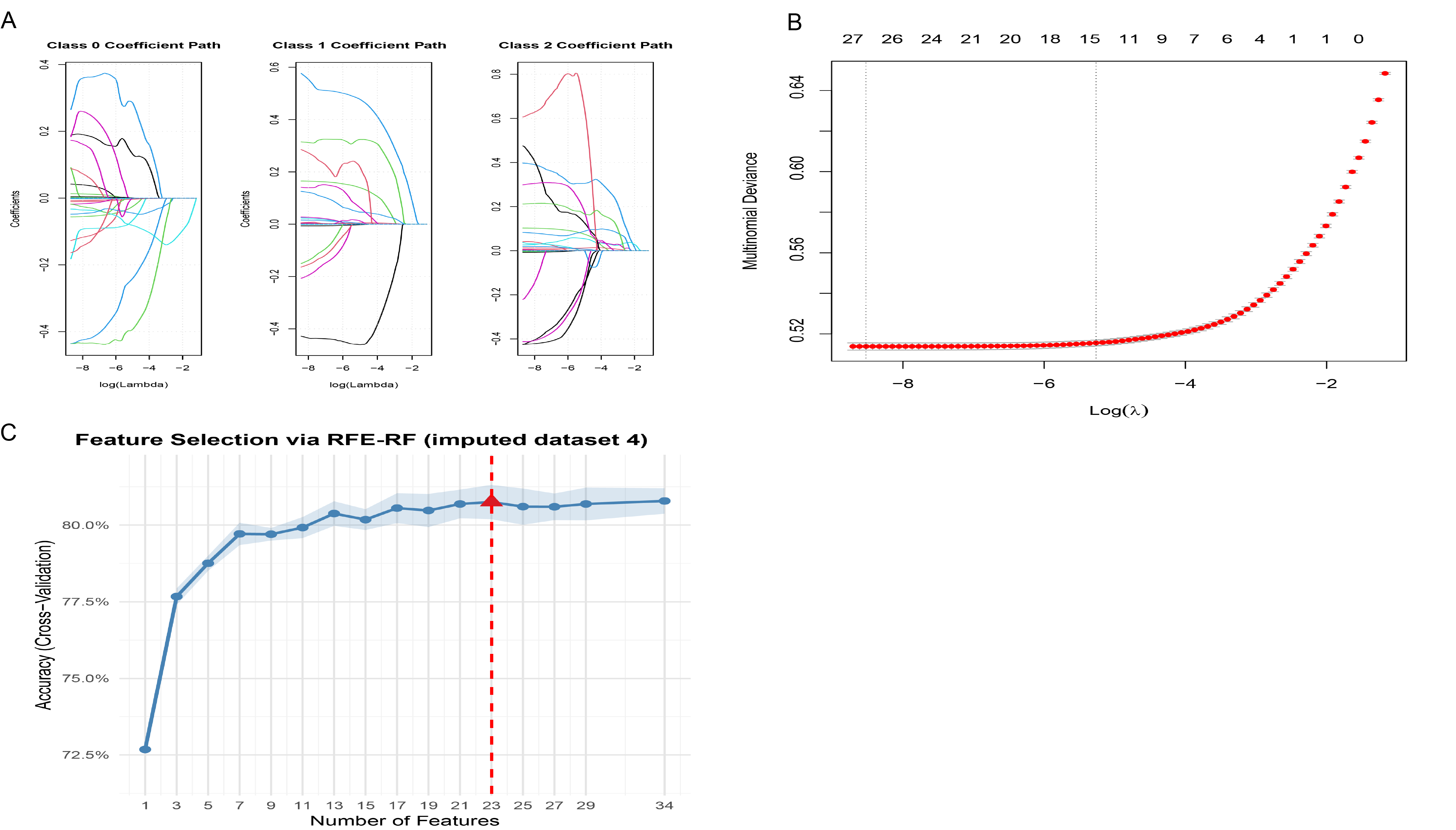


This figure demonstrates the application of Least Absolute Shrinkage and Selection Operator (LASSO) regression and Recursive Feature Elimination based on Random Forest importance (RFE-RF) in the imputed dataset 4 for identifying key predictors associated with hepatic steatosis severity. (A) LASSO regression coefficient profile: Coefficients of candidate variables shrink to zero as the regularization parameter (λ) increases, indicating variable elimination. (B) LASSO cross-validation curve: Binomial deviance is plotted against log(λ), with the vertical dashed lines marking the optimal λ (minimum deviance) and the more conservative λ within 1 standard deviation of the minimum. (C) RFE-RF feature selection curve: Model accuracy (on the validation set) is plotted against the number of selected features, showing the trend of performance change as features are recursively eliminated. LASSO: Least Absolute Shrinkage and Selection Operator, RFE-RF: Recursive Feature Elimination based on Random Forest importance.


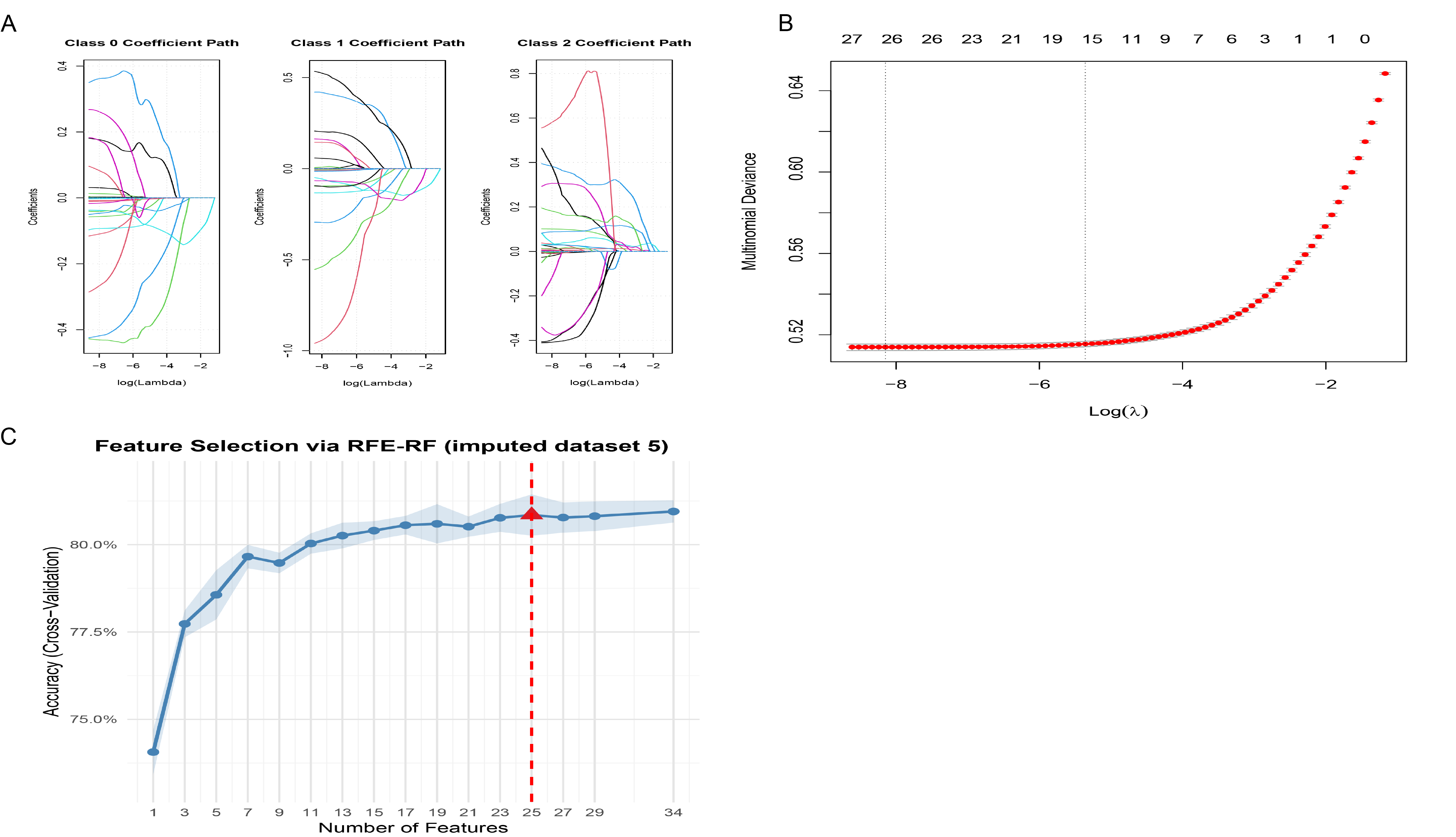


This figure demonstrates the application of Least Absolute Shrinkage and Selection Operator (LASSO) regression and Recursive Feature Elimination based on Random Forest importance (RFE-RF) in the imputed dataset 5 for identifying key predictors associated with hepatic steatosis severity. (A) LASSO regression coefficient profile: Coefficients of candidate variables shrink to zero as the regularization parameter (λ) increases, indicating variable elimination. (B) LASSO cross-validation curve: Binomial deviance is plotted against log(λ), with the vertical dashed lines marking the optimal λ (minimum deviance) and the more conservative λ within 1 standard deviation of the minimum. (C) RFE-RF feature selection curve: Model accuracy (on the validation set) is plotted against the number of selected features, showing the trend of performance change as features are recursively eliminated. LASSO: Least Absolute Shrinkage and Selection Operator, RFE-RF: Recursive Feature Elimination based on Random Forest importance.
