# Supplementary material for: Hepatic Steatosis Severity Prediction in Nonobese Individuals: Machine Learning Model Development and Validation
Source: J Med Internet Res. 2026 Jun 19;28:e82529. doi: 10.2196/82529 (PMC13282044; doi:10.2196/82529)

Multimedia Appendix 6. Performance of Five Alternative Machine Learning Models.

ROC curves for the five comparator algorithms on training set: (A) k-nearest neighbors (KNN), (B) multilayer perceptron (MLP), (C) naive Bayes (NB), (D) random forest (RF), and (E) support vector machine (SVM). Class 0: Non‑steatosis; Class 1: Mild steatosis; Class 2: Moderate‑to‑severe steatosis; ROC: Receiver Operating Characteristic.


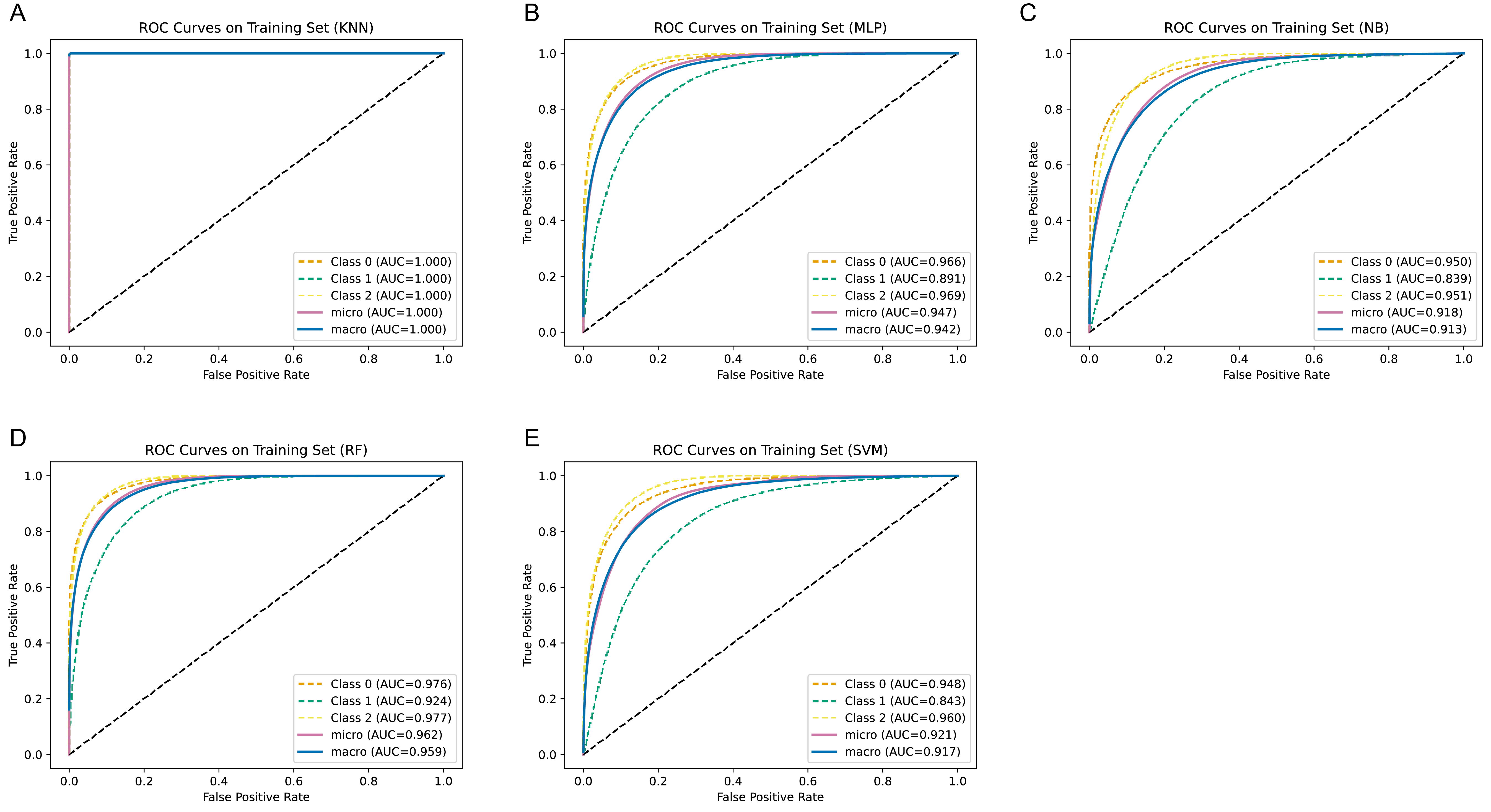


Precision-Recall (PR) curves for the five comparator algorithms on training set: (A) k-nearest neighbors (KNN), (B) multilayer perceptron (MLP), (C) naive Bayes (NB), (D) random forest (RF), and (E) support vector machine (SVM). Class 0: Non‑steatosis; Class 1: Mild steatosis; Class 2: Moderate‑to‑severe steatosis


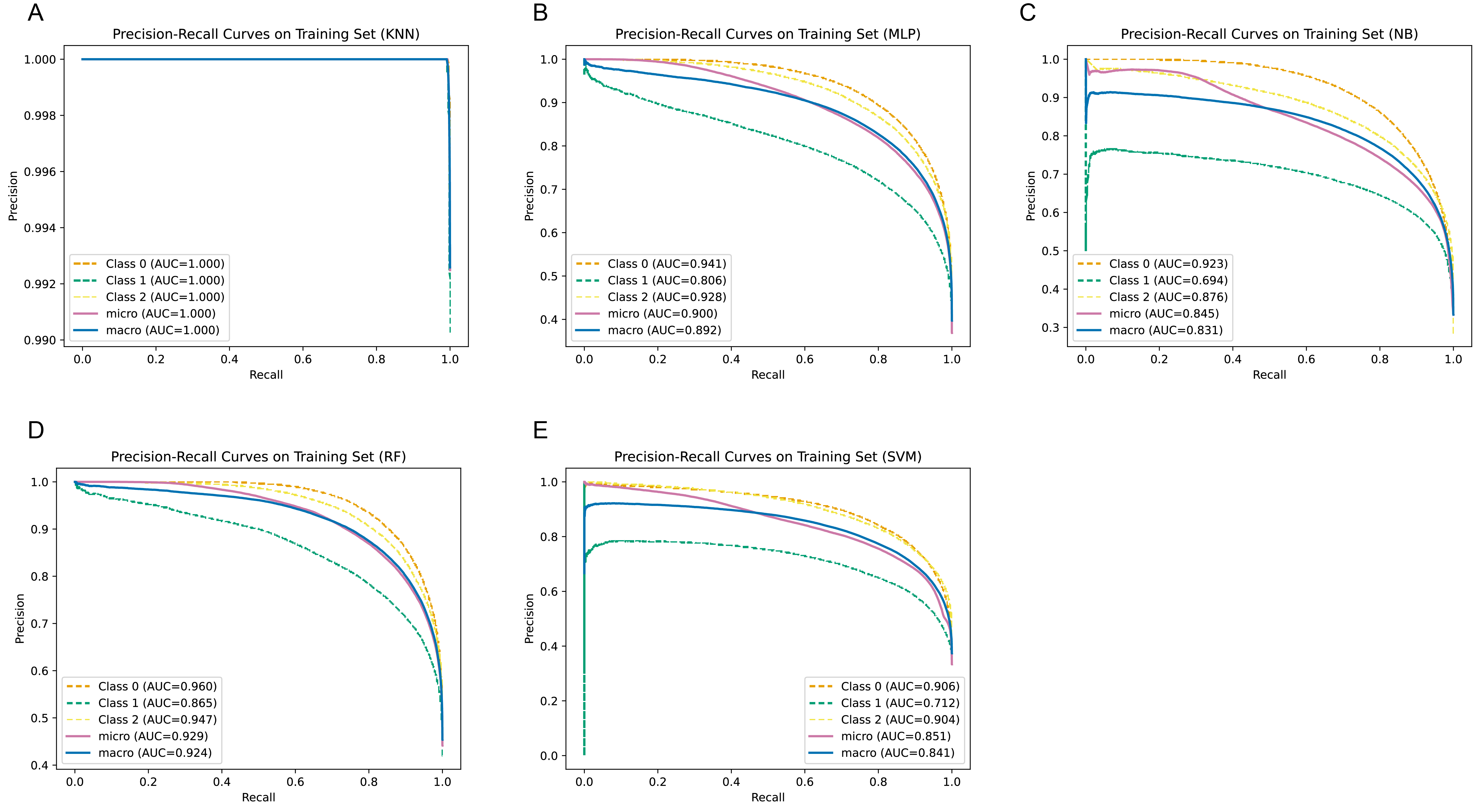


Calibration plots for the five comparator algorithms on training set: (A) k-nearest neighbors (KNN), (B) multilayer perceptron (MLP), (C) naive Bayes (NB), (D) random forest (RF), and (E) support vector machine (SVM).


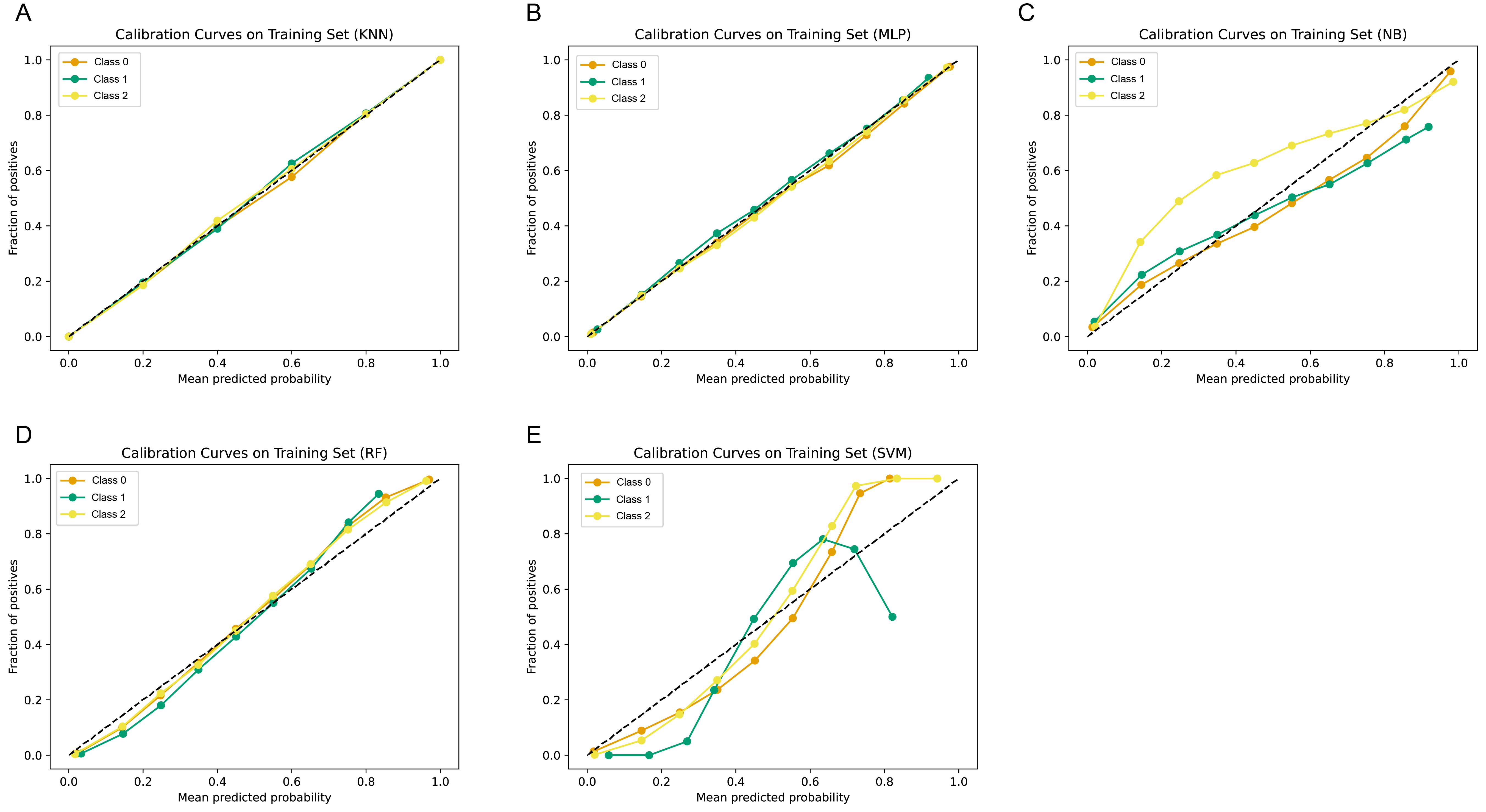


Confusion matrices for the five comparator algorithms on training set: (A) k-nearest neighbors (KNN), (B) multilayer perceptron (MLP), (C) naive Bayes (NB), (D) random forest (RF), and (E) support vector machine (SVM). Class 0: Non‑steatosis; Class 1: Mild steatosis; Class 2: Moderate‑to‑severe steatosis.


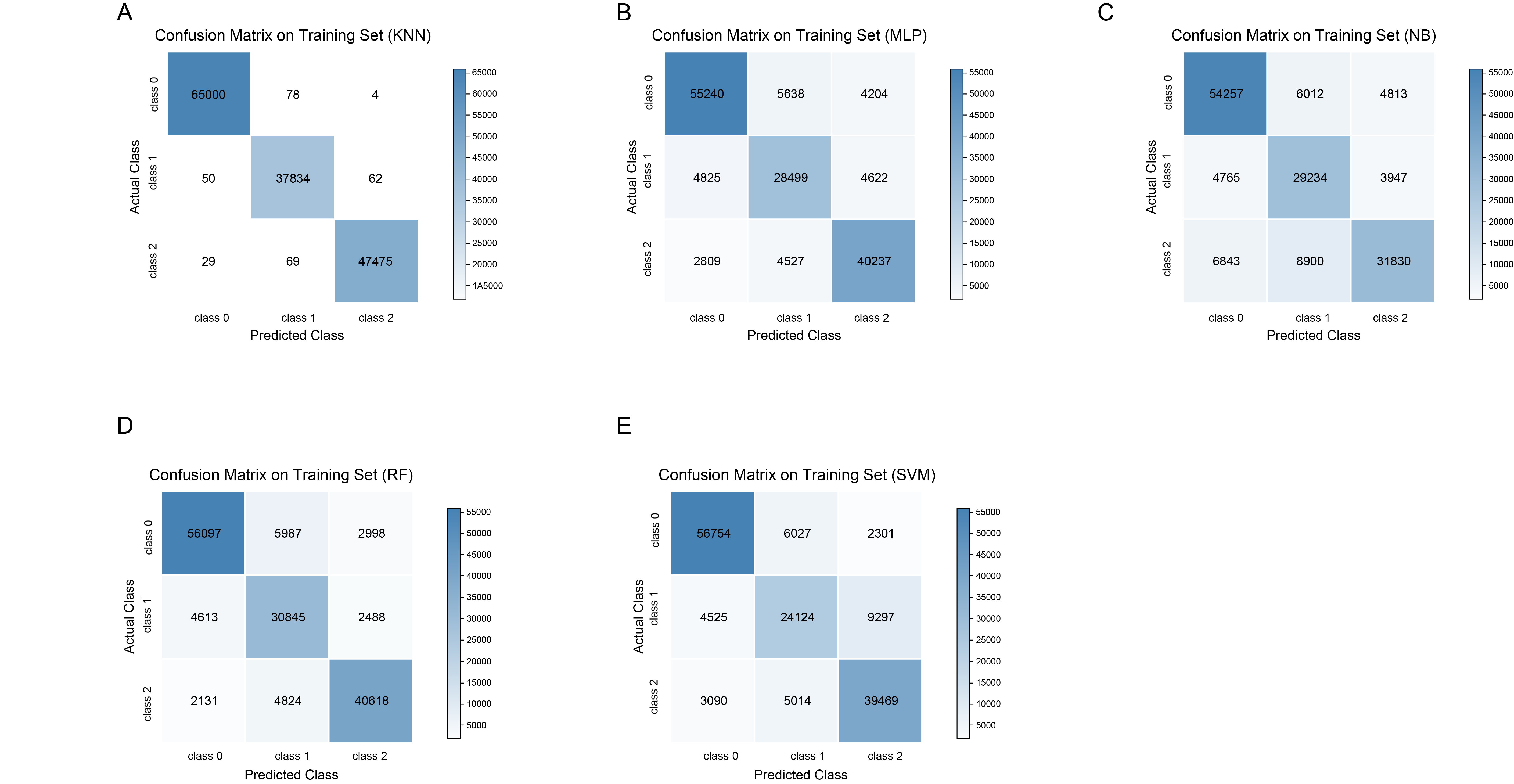


ROC curves for the five comparator algorithms on test set: (A) k-nearest neighbors (KNN), (B) multilayer perceptron (MLP), (C) naive Bayes (NB), (D) random forest (RF), and (E) support vector machine (SVM). Class 0: Non‑steatosis; Class 1: Mild steatosis; Class 2: Moderate‑to‑severe steatosis; ROC: Receiver Operating Characteristic.


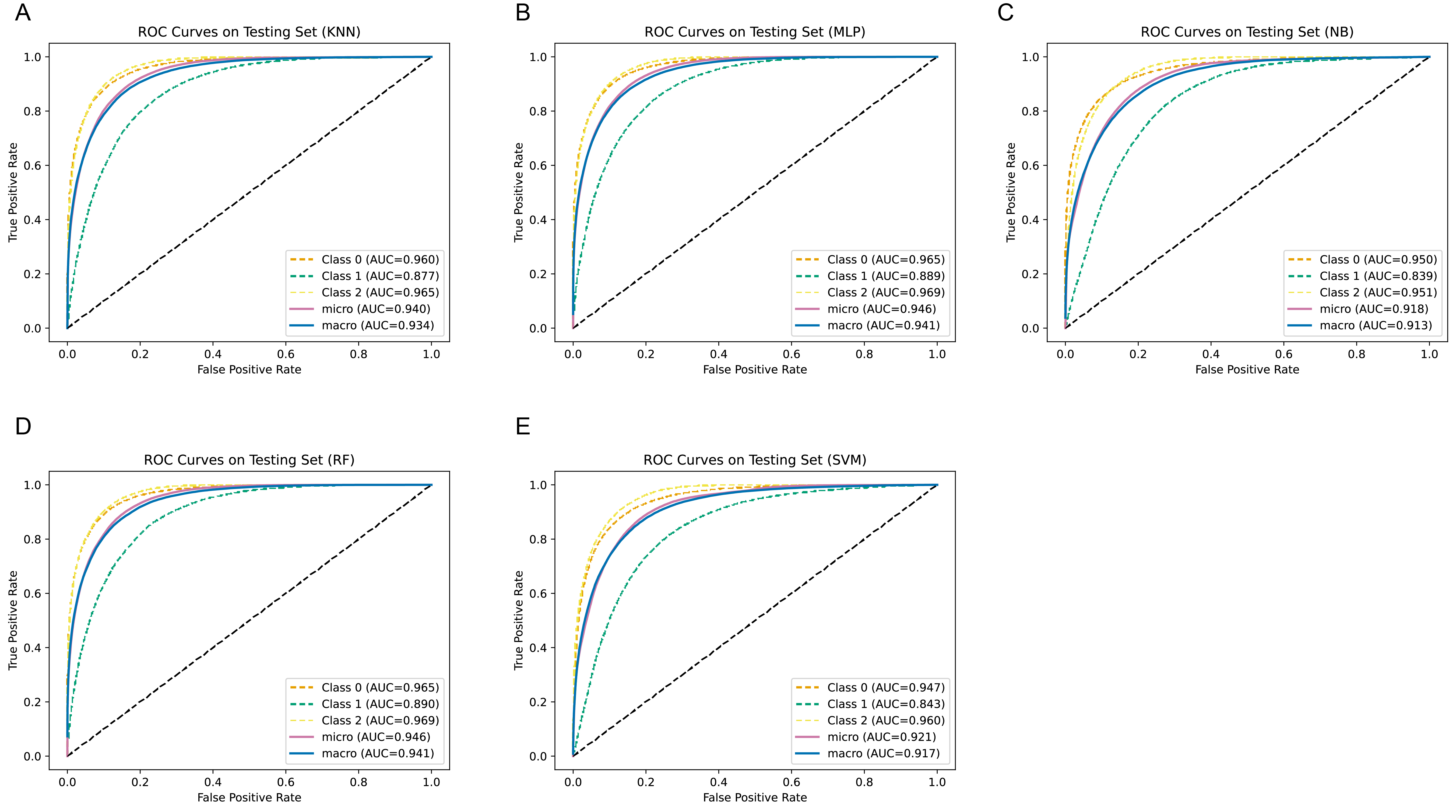


Precision-Recall (PR) curves for the five comparator algorithms on test set: (A) k-nearest neighbors (KNN), (B) multilayer perceptron (MLP), (C) naive Bayes (NB), (D) random forest (RF), and (E) support vector machine (SVM). Class 0: Non‑steatosis; Class 1: Mild steatosis; Class 2: Moderate‑to‑severe steatosis.


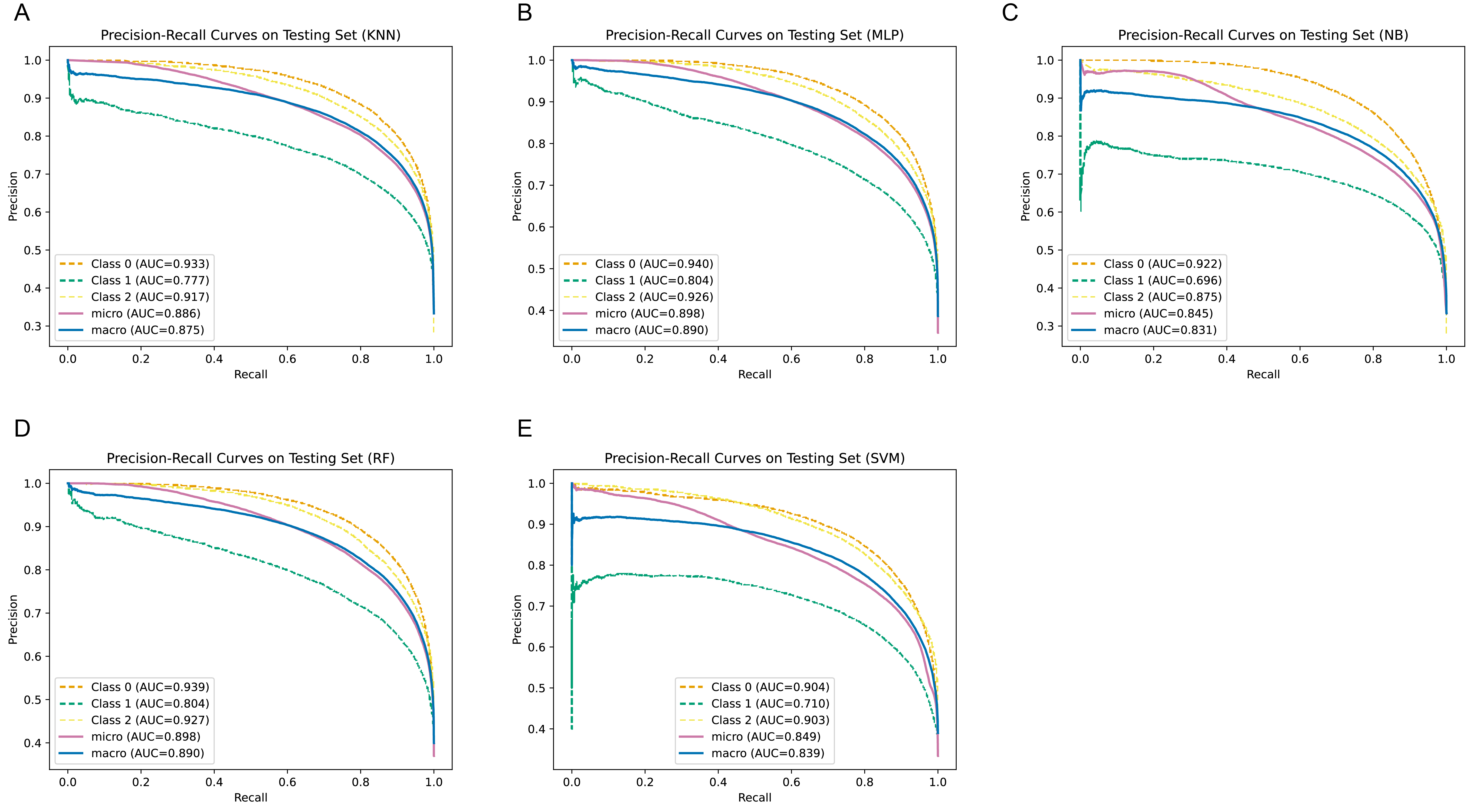


Calibration plots for the five comparator algorithms on test set: (A) k-nearest neighbors (KNN), (B) multilayer perceptron (MLP), (C) naive Bayes (NB), (D) random forest (RF), and (E) support vector machine (SVM).


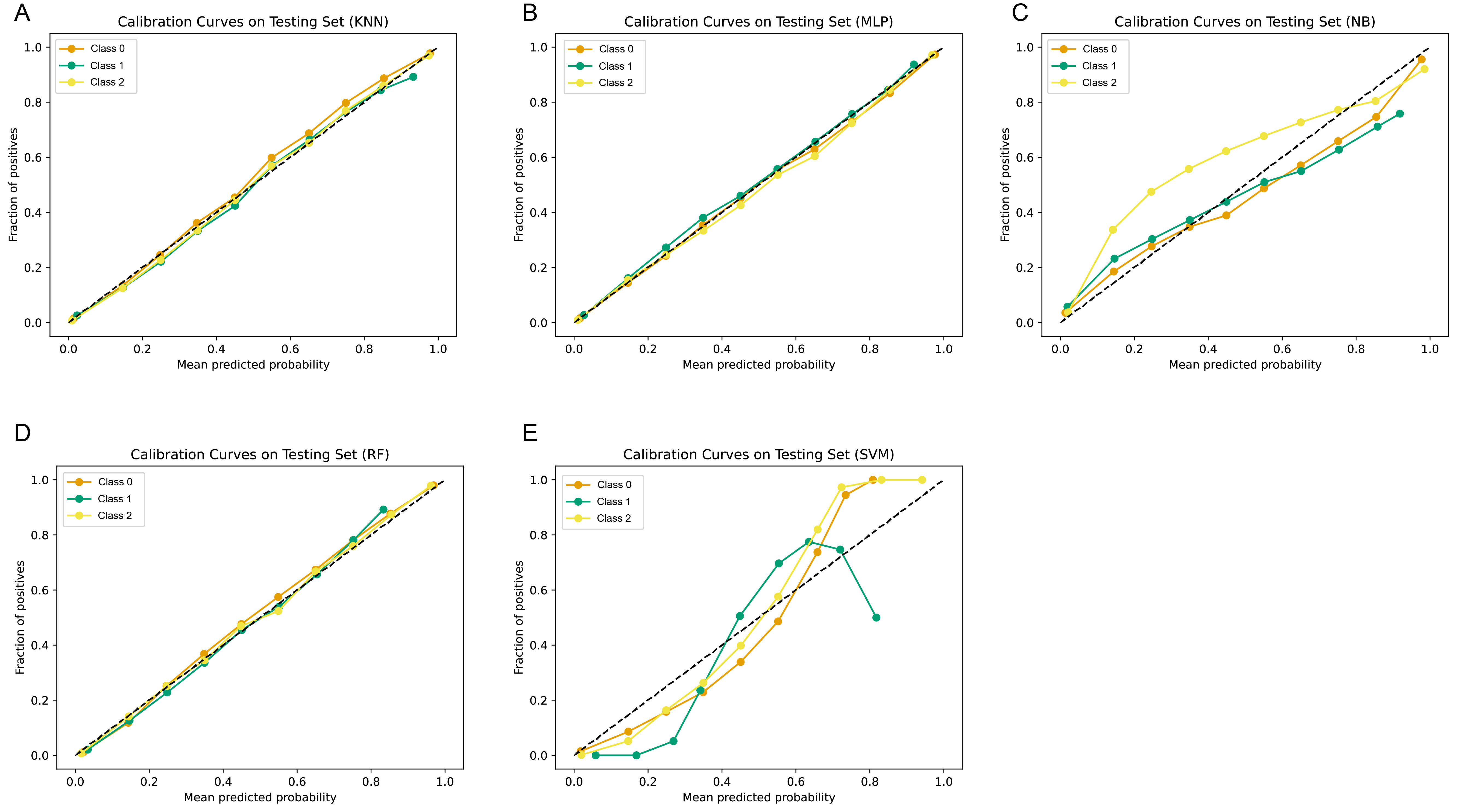


Confusion matrices for the five comparator algorithms on test set: (A) k-nearest neighbors (KNN), (B) multilayer perceptron (MLP), (C) naive Bayes (NB), (D) random forest (RF), and (E) support vector machine (SVM). Class 0: Non‑steatosis; Class 1: Mild steatosis; Class 2: Moderate‑to‑severe steatosis.


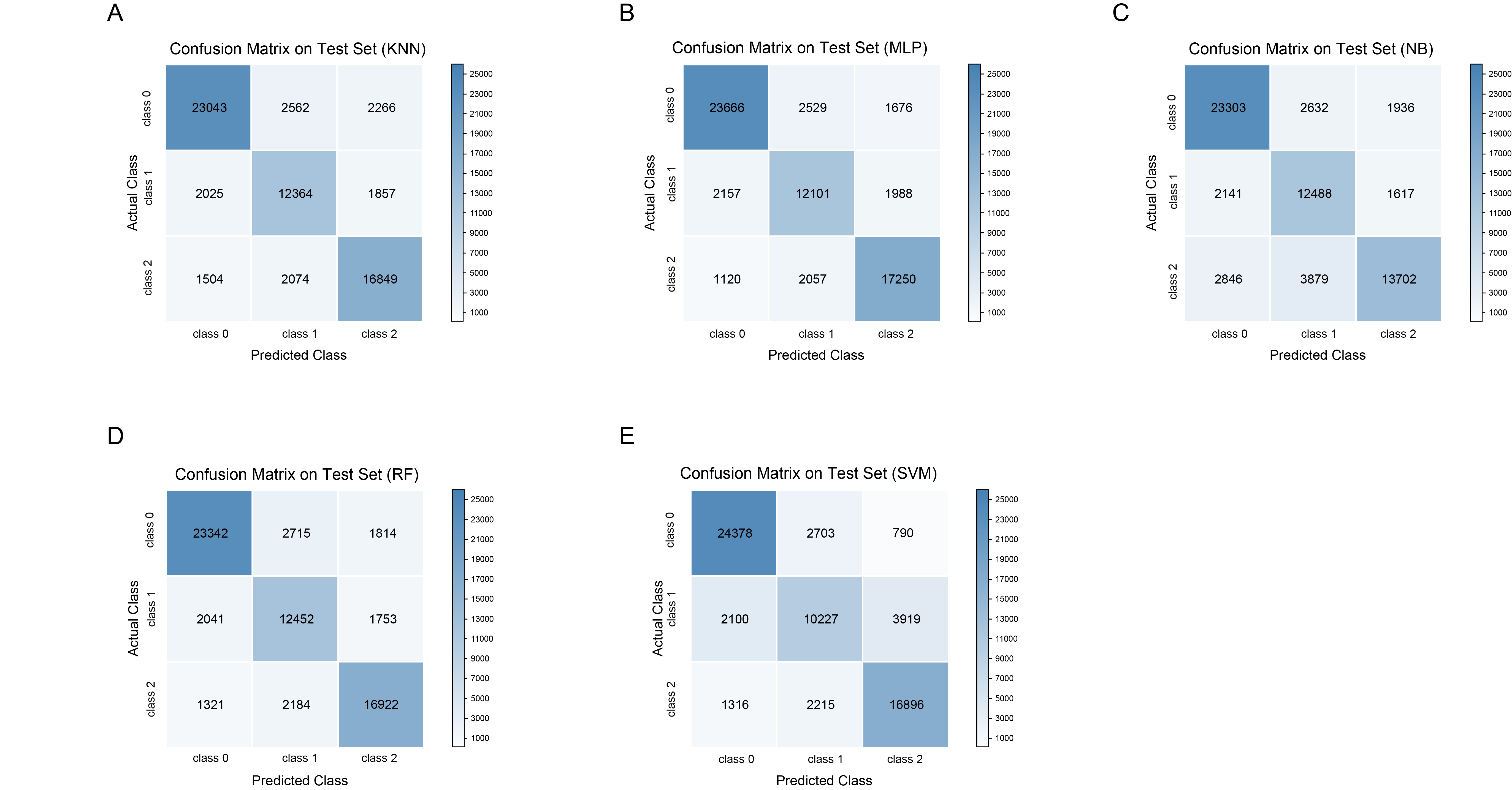

Supplement: Multimedia Appendix 6 [file jmir-v28-e82529-s006.docx]
