# Supplementary material for: Hepatic Steatosis Severity Prediction in Nonobese Individuals: Machine Learning Model Development and Validation
Source: J Med Internet Res. 2026 Jun 19;28:e82529. doi: 10.2196/82529 (PMC13282044; doi:10.2196/82529)
Supplement: Multimedia Appendix 7 [file jmir-v28-e82529-s007.docx]

| Multimedia Appendix 7.1. Performance of each algorithm across the five imputed datasets (training sets) | | | | | | | | | | | | |  |  |  |  |  |  |  |  |  |
| --- | --- | --- | --- | --- | --- | --- | --- | --- | --- | --- | --- | --- | --- | --- | --- | --- | --- | --- | --- | --- | --- |
| Model | Accuracy | Cohen_Kappa | macro_ROC^g^_AUC | micro_ROC_AUC | macro_PR^h^_AUC | micro_PR_AUC | Precision (None) | Recall (None) | Specificity (None) | F1 (None) | ROC_AUC (None) | Precision (Mild) | Recall (Mild) | Specificity (Mild) | F1 (Mild) | ROC_AUC (Mild) | Precision (Moderate to Severe) | Recall (Moderate to Severe) | Specificity (Moderate to Severe) | F1 (Moderate to Severe) | ROC_AUC (Moderate to Severe) |
| Pooled-XGBoost^a^ | 0.828 | 0.721 | 0.945 | 0.950 | 0.897 | 0.905 | 0.882 | 0.843 | 0.915 | 0.862 | 0.967 | 0.739 | 0.786 | 0.906 | 0.762 | 0.896 | 0.832 | 0.840 | 0.922 | 0.836 | 0.971 |
| Imp_1-XGBoost | 0.827 | 0.721 | 0.945 | 0.950 | 0.897 | 0.905 | 0.882 | 0.842 | 0.915 | 0.863 | 0.967 | 0.740 | 0.787 | 0.905 | 0.762 | 0.896 | 0.831 | 0.840 | 0.921 | 0.836 | 0.971 |
| Imp_2-XGBoost | 0.828 | 0.722 | 0.945 | 0.950 | 0.897 | 0.905 | 0.881 | 0.842 | 0.915 | 0.862 | 0.967 | 0.739 | 0.785 | 0.906 | 0.763 | 0.896 | 0.831 | 0.839 | 0.921 | 0.836 | 0.971 |
| Imp_3-XGBoost | 0.829 | 0.722 | 0.945 | 0.950 | 0.897 | 0.905 | 0.881 | 0.842 | 0.914 | 0.863 | 0.967 | 0.739 | 0.787 | 0.907 | 0.763 | 0.896 | 0.832 | 0.839 | 0.922 | 0.837 | 0.971 |
| Imp_4-XGBoost | 0.827 | 0.720 | 0.945 | 0.950 | 0.897 | 0.905 | 0.883 | 0.843 | 0.915 | 0.861 | 0.967 | 0.738 | 0.785 | 0.907 | 0.763 | 0.896 | 0.833 | 0.839 | 0.923 | 0.837 | 0.971 |
| Imp_5-XGBoost | 0.829 | 0.720 | 0.945 | 0.950 | 0.897 | 0.905 | 0.882 | 0.842 | 0.915 | 0.861 | 0.967 | 0.740 | 0.787 | 0.906 | 0.761 | 0.896 | 0.831 | 0.839 | 0.921 | 0.835 | 0.971 |
| Pooled-RF^b^ | 0.847 | 0.760 | 0.959 | 0.962 | 0.924 | 0.929 | 0.893 | 0.862 | 0.921 | 0.877 | 0.976 | 0.740 | 0.813 | 0.904 | 0.775 | 0.924 | 0.881 | 0.854 | 0.947 | 0.867 | 0.977 |
| Imp_1-RF | 0.848 | 0.760 | 0.959 | 0.962 | 0.924 | 0.929 | 0.893 | 0.861 | 0.920 | 0.877 | 0.976 | 0.740 | 0.814 | 0.903 | 0.776 | 0.924 | 0.881 | 0.853 | 0.946 | 0.867 | 0.977 |
| Imp_2-RF | 0.847 | 0.761 | 0.959 | 0.962 | 0.924 | 0.929 | 0.894 | 0.862 | 0.920 | 0.877 | 0.976 | 0.739 | 0.814 | 0.905 | 0.776 | 0.924 | 0.882 | 0.855 | 0.947 | 0.868 | 0.977 |
| Imp_3-RF | 0.846 | 0.760 | 0.959 | 0.962 | 0.924 | 0.929 | 0.893 | 0.861 | 0.920 | 0.876 | 0.976 | 0.739 | 0.814 | 0.903 | 0.774 | 0.924 | 0.882 | 0.855 | 0.946 | 0.867 | 0.977 |
| Imp_4-RF | 0.847 | 0.760 | 0.959 | 0.962 | 0.924 | 0.929 | 0.894 | 0.862 | 0.921 | 0.877 | 0.976 | 0.741 | 0.814 | 0.905 | 0.775 | 0.924 | 0.881 | 0.853 | 0.947 | 0.866 | 0.977 |
| Imp_5-RF | 0.846 | 0.759 | 0.959 | 0.962 | 0.924 | 0.929 | 0.893 | 0.861 | 0.922 | 0.878 | 0.976 | 0.740 | 0.813 | 0.904 | 0.774 | 0.924 | 0.882 | 0.855 | 0.948 | 0.867 | 0.977 |
| Pooled-SVM^c^ | 0.799 | 0.657 | 0.917 | 0.921 | 0.841 | 0.851 | 0.882 | 0.872 | 0.911 | 0.877 | 0.948 | 0.686 | 0.636 | 0.902 | 0.660 | 0.843 | 0.773 | 0.830 | 0.887 | 0.800 | 0.960 |
| Imp_1-SVM | 0.799 | 0.657 | 0.917 | 0.921 | 0.841 | 0.851 | 0.883 | 0.873 | 0.912 | 0.876 | 0.948 | 0.686 | 0.637 | 0.903 | 0.660 | 0.843 | 0.773 | 0.831 | 0.886 | 0.801 | 0.960 |
| Imp_2-SVM | 0.800 | 0.657 | 0.917 | 0.921 | 0.841 | 0.851 | 0.882 | 0.871 | 0.910 | 0.878 | 0.948 | 0.685 | 0.636 | 0.903 | 0.661 | 0.843 | 0.774 | 0.829 | 0.887 | 0.800 | 0.960 |
| Imp_3-SVM | 0.798 | 0.658 | 0.917 | 0.921 | 0.841 | 0.851 | 0.882 | 0.873 | 0.911 | 0.877 | 0.948 | 0.687 | 0.637 | 0.901 | 0.660 | 0.843 | 0.774 | 0.830 | 0.887 | 0.801 | 0.960 |
| Imp_4-SVM | 0.800 | 0.656 | 0.917 | 0.921 | 0.841 | 0.851 | 0.881 | 0.873 | 0.911 | 0.876 | 0.948 | 0.687 | 0.637 | 0.902 | 0.659 | 0.843 | 0.774 | 0.830 | 0.887 | 0.800 | 0.960 |
| Imp_5-SVM | 0.800 | 0.656 | 0.917 | 0.921 | 0.841 | 0.851 | 0.883 | 0.871 | 0.910 | 0.877 | 0.948 | 0.686 | 0.636 | 0.901 | 0.661 | 0.843 | 0.773 | 0.830 | 0.888 | 0.800 | 0.960 |
| Pooled-MLP^d^ | 0.823 | 0.716 | 0.942 | 0.947 | 0.892 | 0.900 | 0.879 | 0.849 | 0.911 | 0.863 | 0.966 | 0.737 | 0.751 | 0.910 | 0.744 | 0.891 | 0.820 | 0.846 | 0.914 | 0.833 | 0.969 |
| Imp_1-MLP | 0.824 | 0.717 | 0.942 | 0.947 | 0.892 | 0.900 | 0.880 | 0.850 | 0.910 | 0.862 | 0.966 | 0.736 | 0.752 | 0.910 | 0.744 | 0.891 | 0.821 | 0.847 | 0.915 | 0.834 | 0.969 |
| Imp_2-MLP | 0.822 | 0.716 | 0.942 | 0.947 | 0.892 | 0.900 | 0.879 | 0.848 | 0.911 | 0.862 | 0.966 | 0.738 | 0.751 | 0.911 | 0.744 | 0.891 | 0.821 | 0.846 | 0.914 | 0.832 | 0.969 |
| Imp_3-MLP | 0.824 | 0.717 | 0.942 | 0.947 | 0.892 | 0.900 | 0.878 | 0.849 | 0.911 | 0.863 | 0.966 | 0.736 | 0.751 | 0.910 | 0.744 | 0.891 | 0.820 | 0.845 | 0.915 | 0.833 | 0.969 |
| Imp_4-MLP | 0.824 | 0.717 | 0.942 | 0.947 | 0.892 | 0.900 | 0.880 | 0.849 | 0.911 | 0.864 | 0.966 | 0.736 | 0.752 | 0.909 | 0.745 | 0.891 | 0.821 | 0.847 | 0.914 | 0.832 | 0.969 |
| Imp_5-MLP | 0.822 | 0.717 | 0.942 | 0.947 | 0.892 | 0.900 | 0.879 | 0.850 | 0.910 | 0.862 | 0.966 | 0.736 | 0.750 | 0.911 | 0.745 | 0.891 | 0.821 | 0.847 | 0.913 | 0.833 | 0.969 |
| Pooled-KNN^e^ | 0.998 | 0.997 | 1.000 | 1.000 | 1.000 | 1.000 | 0.999 | 0.999 | 0.999 | 0.999 | 1.000 | 0.996 | 0.997 | 0.999 | 0.997 | 1.000 | 0.999 | 0.998 | 0.999 | 0.998 | 1.000 |
| Imp_1-KNN | 0.998 | 0.997 | 1.000 | 1.000 | 1.000 | 1.000 | 0.998 | 0.998 | 0.999 | 1.000 | 1.000 | 0.995 | 0.996 | 0.999 | 0.998 | 1.000 | 0.999 | 0.999 | 0.998 | 0.997 | 1.000 |
| Imp_2-KNN | 0.999 | 0.998 | 1.000 | 1.000 | 1.000 | 1.000 | 0.998 | 0.999 | 1.000 | 0.999 | 1.000 | 0.997 | 0.996 | 0.999 | 0.996 | 1.000 | 0.999 | 0.997 | 0.999 | 0.998 | 1.000 |
| Imp_3-KNN | 0.998 | 0.997 | 1.000 | 1.000 | 1.000 | 1.000 | 0.998 | 0.998 | 1.000 | 0.998 | 1.000 | 0.997 | 0.996 | 0.999 | 0.996 | 1.000 | 0.999 | 0.998 | 0.998 | 0.997 | 1.000 |
| Imp_4-KNN | 0.998 | 0.998 | 1.000 | 1.000 | 1.000 | 1.000 | 0.998 | 0.998 | 0.998 | 0.999 | 1.000 | 0.996 | 0.998 | 1.000 | 0.997 | 1.000 | 0.999 | 0.998 | 1.000 | 0.998 | 1.000 |
| Imp_5-KNN | 0.997 | 0.997 | 1.000 | 1.000 | 1.000 | 1.000 | 0.999 | 0.999 | 0.998 | 0.999 | 1.000 | 0.996 | 0.997 | 1.000 | 0.998 | 1.000 | 0.998 | 0.997 | 0.999 | 0.997 | 1.000 |
| Pooled-NB^f^ | 0.766 | 0.640 | 0.913 | 0.918 | 0.831 | 0.845 | 0.824 | 0.834 | 0.864 | 0.829 | 0.950 | 0.662 | 0.770 | 0.868 | 0.712 | 0.839 | 0.784 | 0.669 | 0.915 | 0.722 | 0.951 |
| Imp_1-NB | 0.767 | 0.639 | 0.913 | 0.918 | 0.831 | 0.845 | 0.823 | 0.834 | 0.864 | 0.830 | 0.950 | 0.661 | 0.769 | 0.867 | 0.711 | 0.839 | 0.784 | 0.670 | 0.914 | 0.721 | 0.951 |
| Imp_2-NB | 0.765 | 0.640 | 0.913 | 0.918 | 0.831 | 0.845 | 0.824 | 0.835 | 0.865 | 0.830 | 0.950 | 0.661 | 0.771 | 0.869 | 0.712 | 0.839 | 0.785 | 0.668 | 0.914 | 0.722 | 0.951 |
| Imp_3-NB | 0.767 | 0.640 | 0.913 | 0.918 | 0.831 | 0.845 | 0.825 | 0.833 | 0.864 | 0.830 | 0.950 | 0.663 | 0.770 | 0.869 | 0.711 | 0.839 | 0.785 | 0.670 | 0.915 | 0.721 | 0.951 |
| Imp_4-NB | 0.766 | 0.641 | 0.913 | 0.918 | 0.831 | 0.845 | 0.823 | 0.835 | 0.864 | 0.828 | 0.950 | 0.663 | 0.771 | 0.868 | 0.713 | 0.839 | 0.784 | 0.670 | 0.916 | 0.722 | 0.951 |
| Imp_5-NB | 0.766 | 0.639 | 0.913 | 0.918 | 0.831 | 0.845 | 0.825 | 0.835 | 0.863 | 0.829 | 0.950 | 0.661 | 0.769 | 0.868 | 0.713 | 0.839 | 0.784 | 0.670 | 0.916 | 0.721 | 0.951 |
| Note: aXGBoost: extreme gradient boosting; bRF: random forest; cSVM: support vector machine; dMLP: multilayer perceptron; eKNN: k-nearest neighbors; fNB: naive Bayes; ᵍROC-AUC: area under the receiver operating characteristic curve; ʰPR-AUC: area under the precision-recall curve. | | | | | | | | | | | | | | | | | | | | | |

| Multimedia Appendix 7.2. Performance of each algorithm across the five imputed datasets (test sets) | | | | | | | | | | | | |  |  |  |  |  |  |  |  |  |
| --- | --- | --- | --- | --- | --- | --- | --- | --- | --- | --- | --- | --- | --- | --- | --- | --- | --- | --- | --- | --- | --- |
| Model | Accuracy | Cohen_Kappa | macro_ROC^g^_AUC | micro_ROC_AUC | macro_PR^h^_AUC | micro_PR_AUC | Precision (None) | Recall (None) | Specificity (None) | F1 (None) | ROC_AUC (None) | Precision (Mild) | Recall (Mild) | Specificity (Mild) | F1 (Mild) | ROC_AUC (Mild) | Precision (Moderate to Severe) | Recall (Moderate to Severe) | Specificity (Moderate to Severe) | F1 (Moderate to Severe) | ROC_AUC (Moderate to Severe) |
| Pooled-XGBoost^a^ | 0.824 | 0.713 | 0.941 | 0.946 | 0.890 | 0.899 | 0.879 | 0.840 | 0.912 | 0.859 | 0.965 | 0.731 | 0.783 | 0.903 | 0.756 | 0.890 | 0.832 | 0.836 | 0.922 | 0.834 | 0.969 |
| Imp_1-XGBoost | 0.825 | 0.712 | 0.941 | 0.946 | 0.890 | 0.899 | 0.879 | 0.839 | 0.914 | 0.858 | 0.965 | 0.731 | 0.782 | 0.902 | 0.754 | 0.890 | 0.832 | 0.836 | 0.922 | 0.836 | 0.969 |
| Imp_2-XGBoost | 0.823 | 0.712 | 0.941 | 0.946 | 0.890 | 0.899 | 0.880 | 0.840 | 0.914 | 0.860 | 0.965 | 0.729 | 0.782 | 0.901 | 0.755 | 0.890 | 0.832 | 0.836 | 0.922 | 0.835 | 0.969 |
| Imp_3-XGBoost | 0.825 | 0.714 | 0.941 | 0.946 | 0.890 | 0.899 | 0.880 | 0.839 | 0.912 | 0.859 | 0.965 | 0.730 | 0.783 | 0.903 | 0.755 | 0.890 | 0.834 | 0.837 | 0.923 | 0.834 | 0.969 |
| Imp_4-XGBoost | 0.825 | 0.712 | 0.941 | 0.946 | 0.890 | 0.899 | 0.878 | 0.841 | 0.913 | 0.859 | 0.965 | 0.731 | 0.784 | 0.901 | 0.755 | 0.890 | 0.832 | 0.835 | 0.922 | 0.835 | 0.969 |
| Imp_5-XGBoost | 0.824 | 0.714 | 0.941 | 0.946 | 0.890 | 0.899 | 0.880 | 0.841 | 0.914 | 0.858 | 0.965 | 0.731 | 0.784 | 0.903 | 0.755 | 0.890 | 0.834 | 0.835 | 0.924 | 0.835 | 0.969 |
| Pooled-RF^b^ | 0.817 | 0.711 | 0.941 | 0.946 | 0.890 | 0.898 | 0.874 | 0.838 | 0.908 | 0.855 | 0.965 | 0.718 | 0.766 | 0.899 | 0.741 | 0.890 | 0.826 | 0.828 | 0.919 | 0.827 | 0.969 |
| Imp_1-RF | 0.818 | 0.711 | 0.941 | 0.946 | 0.890 | 0.898 | 0.873 | 0.837 | 0.907 | 0.856 | 0.965 | 0.718 | 0.766 | 0.900 | 0.742 | 0.890 | 0.827 | 0.828 | 0.918 | 0.827 | 0.969 |
| Imp_2-RF | 0.818 | 0.710 | 0.941 | 0.946 | 0.890 | 0.898 | 0.875 | 0.839 | 0.909 | 0.854 | 0.965 | 0.718 | 0.767 | 0.898 | 0.742 | 0.890 | 0.825 | 0.828 | 0.918 | 0.827 | 0.969 |
| Imp_3-RF | 0.818 | 0.710 | 0.941 | 0.946 | 0.890 | 0.898 | 0.875 | 0.838 | 0.907 | 0.855 | 0.965 | 0.719 | 0.767 | 0.898 | 0.742 | 0.890 | 0.825 | 0.828 | 0.920 | 0.827 | 0.969 |
| Imp_4-RF | 0.817 | 0.710 | 0.941 | 0.946 | 0.890 | 0.898 | 0.873 | 0.837 | 0.908 | 0.855 | 0.965 | 0.718 | 0.766 | 0.900 | 0.742 | 0.890 | 0.825 | 0.829 | 0.918 | 0.827 | 0.969 |
| Imp_5-RF | 0.817 | 0.711 | 0.941 | 0.946 | 0.890 | 0.898 | 0.873 | 0.839 | 0.909 | 0.855 | 0.965 | 0.719 | 0.767 | 0.899 | 0.740 | 0.890 | 0.825 | 0.827 | 0.919 | 0.826 | 0.969 |
| Pooled-SVM^c^ | 0.798 | 0.654 | 0.917 | 0.921 | 0.839 | 0.849 | 0.877 | 0.875 | 0.907 | 0.876 | 0.947 | 0.675 | 0.630 | 0.898 | 0.652 | 0.843 | 0.782 | 0.827 | 0.893 | 0.804 | 0.960 |
| Imp_1-SVM | 0.798 | 0.655 | 0.917 | 0.921 | 0.839 | 0.849 | 0.877 | 0.874 | 0.906 | 0.875 | 0.947 | 0.675 | 0.630 | 0.899 | 0.653 | 0.843 | 0.782 | 0.828 | 0.893 | 0.804 | 0.960 |
| Imp_2-SVM | 0.797 | 0.653 | 0.917 | 0.921 | 0.839 | 0.849 | 0.876 | 0.874 | 0.908 | 0.877 | 0.947 | 0.674 | 0.630 | 0.897 | 0.653 | 0.843 | 0.783 | 0.828 | 0.894 | 0.805 | 0.960 |
| Imp_3-SVM | 0.797 | 0.654 | 0.917 | 0.921 | 0.839 | 0.849 | 0.876 | 0.874 | 0.906 | 0.875 | 0.947 | 0.675 | 0.631 | 0.897 | 0.651 | 0.843 | 0.781 | 0.828 | 0.892 | 0.805 | 0.960 |
| Imp_4-SVM | 0.799 | 0.653 | 0.917 | 0.921 | 0.839 | 0.849 | 0.876 | 0.876 | 0.906 | 0.877 | 0.947 | 0.676 | 0.630 | 0.897 | 0.653 | 0.843 | 0.783 | 0.827 | 0.894 | 0.805 | 0.960 |
| Imp_5-SVM | 0.798 | 0.654 | 0.917 | 0.921 | 0.839 | 0.849 | 0.878 | 0.876 | 0.908 | 0.875 | 0.947 | 0.675 | 0.631 | 0.898 | 0.651 | 0.843 | 0.783 | 0.828 | 0.894 | 0.804 | 0.960 |
| Pooled-MLP^d^ | 0.821 | 0.712 | 0.941 | 0.946 | 0.890 | 0.898 | 0.878 | 0.849 | 0.911 | 0.864 | 0.965 | 0.725 | 0.745 | 0.905 | 0.735 | 0.889 | 0.825 | 0.844 | 0.917 | 0.835 | 0.969 |
| Imp_1-MLP | 0.822 | 0.713 | 0.941 | 0.946 | 0.890 | 0.898 | 0.879 | 0.850 | 0.910 | 0.863 | 0.965 | 0.724 | 0.745 | 0.906 | 0.734 | 0.889 | 0.824 | 0.844 | 0.918 | 0.834 | 0.969 |
| Imp_2-MLP | 0.822 | 0.711 | 0.941 | 0.946 | 0.890 | 0.898 | 0.879 | 0.849 | 0.912 | 0.863 | 0.965 | 0.724 | 0.744 | 0.905 | 0.734 | 0.889 | 0.825 | 0.844 | 0.917 | 0.834 | 0.969 |
| Imp_3-MLP | 0.821 | 0.713 | 0.941 | 0.946 | 0.890 | 0.898 | 0.877 | 0.849 | 0.911 | 0.863 | 0.965 | 0.726 | 0.746 | 0.905 | 0.736 | 0.889 | 0.824 | 0.844 | 0.918 | 0.836 | 0.969 |
| Imp_4-MLP | 0.821 | 0.711 | 0.941 | 0.946 | 0.890 | 0.898 | 0.878 | 0.849 | 0.912 | 0.863 | 0.965 | 0.726 | 0.745 | 0.905 | 0.735 | 0.889 | 0.824 | 0.843 | 0.918 | 0.836 | 0.969 |
| Imp_5-MLP | 0.822 | 0.711 | 0.941 | 0.946 | 0.890 | 0.898 | 0.877 | 0.850 | 0.912 | 0.864 | 0.965 | 0.726 | 0.745 | 0.905 | 0.736 | 0.889 | 0.826 | 0.845 | 0.917 | 0.835 | 0.969 |
| Pooled-KNN^e^ | 0.810 | 0.700 | 0.934 | 0.940 | 0.875 | 0.886 | 0.867 | 0.827 | 0.904 | 0.847 | 0.960 | 0.727 | 0.761 | 0.904 | 0.744 | 0.877 | 0.803 | 0.825 | 0.907 | 0.814 | 0.965 |
| Imp_1-KNN | 0.810 | 0.699 | 0.934 | 0.940 | 0.875 | 0.886 | 0.867 | 0.828 | 0.905 | 0.847 | 0.960 | 0.728 | 0.761 | 0.903 | 0.744 | 0.877 | 0.804 | 0.826 | 0.908 | 0.813 | 0.965 |
| Imp_2-KNN | 0.809 | 0.700 | 0.934 | 0.940 | 0.875 | 0.886 | 0.866 | 0.826 | 0.904 | 0.847 | 0.960 | 0.727 | 0.762 | 0.905 | 0.744 | 0.877 | 0.802 | 0.825 | 0.906 | 0.815 | 0.965 |
| Imp_3-KNN | 0.809 | 0.701 | 0.934 | 0.940 | 0.875 | 0.886 | 0.866 | 0.828 | 0.904 | 0.846 | 0.960 | 0.726 | 0.761 | 0.905 | 0.745 | 0.877 | 0.803 | 0.825 | 0.908 | 0.813 | 0.965 |
| Imp_4-KNN | 0.810 | 0.700 | 0.934 | 0.940 | 0.875 | 0.886 | 0.867 | 0.827 | 0.903 | 0.846 | 0.960 | 0.728 | 0.760 | 0.903 | 0.743 | 0.877 | 0.803 | 0.826 | 0.906 | 0.815 | 0.965 |
| Imp_5-KNN | 0.811 | 0.699 | 0.934 | 0.940 | 0.875 | 0.886 | 0.867 | 0.827 | 0.903 | 0.848 | 0.960 | 0.728 | 0.762 | 0.903 | 0.745 | 0.877 | 0.803 | 0.826 | 0.906 | 0.814 | 0.965 |
| Pooled-NB^f^ | 0.767 | 0.641 | 0.913 | 0.918 | 0.831 | 0.845 | 0.824 | 0.836 | 0.864 | 0.830 | 0.950 | 0.657 | 0.769 | 0.865 | 0.709 | 0.839 | 0.794 | 0.671 | 0.919 | 0.727 | 0.951 |
| Imp_1-NB | 0.767 | 0.640 | 0.913 | 0.918 | 0.831 | 0.845 | 0.824 | 0.835 | 0.864 | 0.829 | 0.950 | 0.657 | 0.770 | 0.864 | 0.709 | 0.839 | 0.793 | 0.670 | 0.920 | 0.728 | 0.951 |
| Imp_2-NB | 0.767 | 0.640 | 0.913 | 0.918 | 0.831 | 0.845 | 0.824 | 0.836 | 0.864 | 0.831 | 0.950 | 0.657 | 0.770 | 0.865 | 0.709 | 0.839 | 0.794 | 0.672 | 0.919 | 0.728 | 0.951 |
| Imp_3-NB | 0.768 | 0.640 | 0.913 | 0.918 | 0.831 | 0.845 | 0.825 | 0.836 | 0.865 | 0.830 | 0.950 | 0.658 | 0.770 | 0.866 | 0.709 | 0.839 | 0.795 | 0.672 | 0.919 | 0.728 | 0.951 |
| Imp_4-NB | 0.766 | 0.640 | 0.913 | 0.918 | 0.831 | 0.845 | 0.825 | 0.836 | 0.863 | 0.831 | 0.950 | 0.656 | 0.770 | 0.864 | 0.708 | 0.839 | 0.795 | 0.671 | 0.919 | 0.728 | 0.951 |
| Imp_5-NB | 0.766 | 0.642 | 0.913 | 0.918 | 0.831 | 0.845 | 0.825 | 0.836 | 0.863 | 0.831 | 0.950 | 0.656 | 0.769 | 0.864 | 0.708 | 0.839 | 0.794 | 0.671 | 0.918 | 0.727 | 0.951 |
| Note: aXGBoost: extreme gradient boosting; bRF: random forest; cSVM: support vector machine; dMLP: multilayer perceptron; eKNN: k-nearest neighbors; fNB: naive Bayes; ᵍROC-AUC: area under the receiver operating characteristic curve; ʰPR-AUC: area under the precision-recall curve. | | | | | | | | | | | | | | | | | | | | | |
