# Supplementary material for: Hepatic Steatosis Severity Prediction in Nonobese Individuals: Machine Learning Model Development and Validation
Source: J Med Internet Res. 2026 Jun 19;28:e82529. doi: 10.2196/82529 (PMC13282044; doi:10.2196/82529)
Supplement: Multimedia Appendix 8 [file jmir-v28-e82529-s008.doc]

| Multimedia Appendix 8. The optimal hyperparameters of each algorithm. | |
| --- | --- |
| Model | Hyperparameters |
| KNN^a^ | {'n_neighbors': 25, 'weights': 'distance', 'metric': 'minkowski', 'p': 2, 'n_jobs': -1, 'scale': TRUE} |
| MLP^b^ | {'activation': 'relu', 'alpha': 0.0001, 'batch_size': 1024, 'beta_1': 0.9, 'beta_2': 0.999, 'early_stopping': True, 'epsilon': 1e-08, 'hidden_layer_sizes': (128, 32), 'learning_rate': 'adaptive', 'learning_rate_init': 0.001, 'max_fun': 15000, 'max_iter': 200, 'momentum': 0.9, 'n_iter_no_change': 10, 'nesterovs_momentum': True, 'power_t': 0.5, 'random_state': 42, 'shuffle': True, 'solver': 'adam', 'tol': 0.0001, 'validation_fraction': 0.1, 'verbose': False, 'warm_start': False} |
| NB^c^ | {'priors': None, 'var_smoothing': 1e-09} |
| RF^d^ | {'bootstrap': TRUE, 'ccp_alpha': 0.0, 'class_weight': None, 'criterion': 'gini', 'max_depth': 12, 'max_features': 'sqrt', 'max_leaf_nodes': None, 'max_samples': None, 'min_impurity_decrease': 0.0, 'min_samples_leaf': 4, 'min_samples_split': 10, 'min_weight_fraction_leaf': 0.0, 'n_estimators': 500, 'n_jobs': -1, 'oob_score': FALSE, 'random_state': 42, 'verbose': 0, 'warm_start': FALSE} |
| SVM^e^ | {"loss": "hinge","penalty": "l2", "alpha": 0.0001, "max_iter": 1000, "tol": 0.001, "learning_rate": "optimal"} |
| XGBoost^f^ | {'objective': 'multi:softprob', 'num_class': 3, 'eval_metric': 'mlogloss', 'n_estimators': 800, 'learning_rate': 0.03, 'max_depth': 3, 'subsample': 0.8, 'colsample_bytree': 0.8, 'min_child_weight': 3, 'gamma': 0.1, 'reg_alpha': 0.1, 'reg_lambda': 1.0, 'random_state': 42} |

Note: Visualization of predicted versus true class assignments for six models. ᵃKNN: k-nearest neighbors; ᵇMLP: multilayer perceptron; ᶜNB: naive Bayes; ᵈRF: random forest; ᵉSVM: support vector machine; ᶠXGBoost: extreme gradient boosting.
