# Supplementary material for: Hepatic Steatosis Severity Prediction in Nonobese Individuals: Machine Learning Model Development and Validation
Source: J Med Internet Res. 2026 Jun 19;28:e82529. doi: 10.2196/82529 (PMC13282044; doi:10.2196/82529)

Multimedia Appendix 9. Sample size distribution by hepatic steatosis grade in the internal cohort and external NHANES validation cohort.


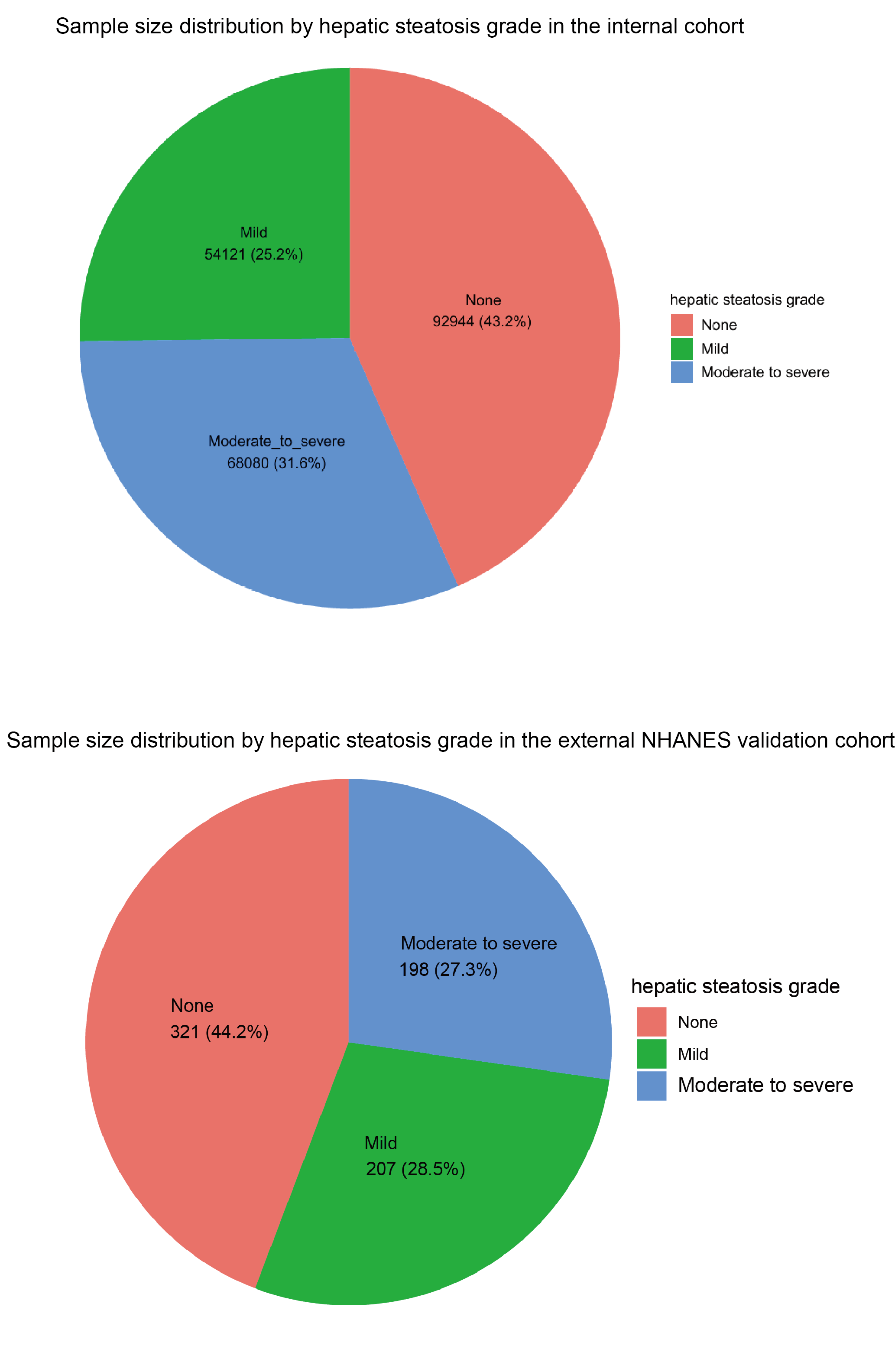

Supplement: Multimedia Appendix 9 [file jmir-v28-e82529-s009.docx]
