# Supplementary material for: Hepatic Steatosis Severity Prediction in Nonobese Individuals: Machine Learning Model Development and Validation
Source: J Med Internet Res. 2026 Jun 19;28:e82529. doi: 10.2196/82529 (PMC13282044; doi:10.2196/82529)
Supplement: Multimedia Appendix 10 [file jmir-v28-e82529-s010.docx]

| Multimedia Appendix 10. Baseline characteristics of the NHANES external validation cohort | | | | | |
| --- | --- | --- | --- | --- | --- |
| Variables | Total (n = 726) | None (n = 321) | Mild (n = 207) | Moderate to severe (n = 198) | P^q^ |
| Age (year) | 48.00 (33.00, 61.00) | 40.00 (28.00,55.00) | 50.00 (39.00,63.00) | 55.00 (46.00,63.25) | <.001 |
| Gender, n (%) |  |  |  |  | <.001 |
| Female | 384 (52.89) | 197 (61.37) | 105 (50.72) | 82 (41.41) |  |
| Male | 342 (47.11) | 124 (38.63) | 102 (49.28) | 116 (58.59) |  |
| SBP^a^ (mmHg) | 120.33  (109.00, 131.17) | 114.83 (103.83,125.67) | 122.00 (112.67,133.67) | 124.83  (115.25,136.58) | <.001 |
| DBP^b^ (mmHg) | 73.00 (66.00, 79.33) | 69.33 (63.67,76.33) | 74.33 (66.67,79.33) | 75.67 (70.08,82.50) | <.001 |
| BMI^c^ (kg/m^2^) | 23.80 (21.72, 25.70) | 22.40 (20.60,24.40) | 24.20 (22.70,25.70) | 25.20 (23.60,26.80) | <.001 |
| WC^d^ (cm) | 85.80 (78.93, 91.90) | 80.25 (74.30,86.00) | 87.20 (82.60,92.00) | 91.75 (86.88,95.30) | <.001 |
| HDL^e^ (mmol/L) | 1.24 (1.02, 1.48) | 1.36 (1.14,1.59) | 1.25 (1.08,1.41) | 1.13 (0.95,1.24) | <.001 |
| TG^f^ (mmol/L) | 1.22 (0.84, 1.78) | 0.92 (0.64,1.31) | 1.28 (0.95,1.85) | 1.63(1.11,2.49) | <.001 |
| LDL^g^ (mmol/L) | 2.77 (2.20, 3.41) | 2.72 (2.12,3.31) | 2.82 (2.17,3.62) | 3.00 (2.40,3.54) | .059 |
| FBG^h^ (mmol/L) | 5.29 (5.01, 5.62) | 5.21(4.78,5.42) | 5.36 (5.05,5.76) | 5.47 (5.02,6.03) | <.001 |
| ALT^i^ (U/L) | 19.00 (13.00, 29.00) | 17.00 (12.00,22.00) | 20.00 (15.00,28.00) | 26.00 (18.00,34.00) | <.001 |
| ALB^j^ (g/L) | 44.50 (44.10, 46.70) | 44.30 (44.00,46.50) | 44.80 (44.50,46.20) | 45.30 (44.20,47.10) | .203 |
| ALP^k^ (IU/L) | 69.00 (56.00, 83.00) | 64.00 (53.00,80.00) | 69.00 (57.00,81.00) | 73.00 (61.00,88.00) | <.001 |
| AST^l^ (U/L) | 20.00 (16.00, 24.00) | 19.00 (16.00,22.00) | 20.00 (17.00,23.00) | 23.00 (19.00,28.00) | <.001 |
| SCr^m^ (μmol/L) | 67.00 (56.00, 80.00) | 65.00 (54.00,78.00) | 69.00 (59.00,79.00) | 70.00 (61.00,82.00) | .116 |
| UA^n^ (μmol/L) | 348.00  (291.00, 405.00) | 345.00 (281.00,402.00) | 357.00 (302.00,418.00) | 381.00  (335.00,447.00) | .96 |
| CMetS^o^ | 0.21 (-0.56, 0.40) | -0.28 (-0.60,0.11) | 0.22 (-0.14,0.59) | 0.64 (0.25,0.99) | <.001 |
| TyG^p^ | 8.45 (7.99, 8.92) | 8.10 (7.77,8.53) | 8.46 (8.03,8.87) | 9.00 (8.64,9.41) | <.001 |
| Note: ᵃSBP: systolic blood pressure; ᵇDBP: diastolic blood pressure; ᶜBMI: body mass index; ᵈWC: waist circumference; ᵉHDL: high-density lipoprotein cholesterol; ᶠTG: triglycerides; ᵍLDL: low-density lipoprotein cholesterol; ʰFBG: fasting blood glucose; ⁱALT: alanine aminotransferase; ʲALB: albumin; ᵏALP: alkaline phosphatase; ˡAST: aspartate aminotransferase; ᵐSCr: serum creatinine; ⁿUA: uric acid; ᵒCMetS: the continuous metabolic syndrome score; ᵖTyG: the triglyceride-glucose index. Continuous variables were characterized as median (IQR), while categorical variables were described in terms of frequency (percentage). P^q^ values for continuous variables were calculated using the Kruskal-Wallis H test and those for categorical variables were calculated using Pearson’s chi-squared test. | | | | | |
