# Supplementary material for: Hepatic Steatosis Severity Prediction in Nonobese Individuals: Machine Learning Model Development and Validation
Source: J Med Internet Res. 2026 Jun 19;28:e82529. doi: 10.2196/82529 (PMC13282044; doi:10.2196/82529)
Supplement: Multimedia Appendix 11 [file jmir-v28-e82529-s011.docx]

Multimedia Appendix 11. External validation and model comparison using receiver operating characteristic (ROC) curves.


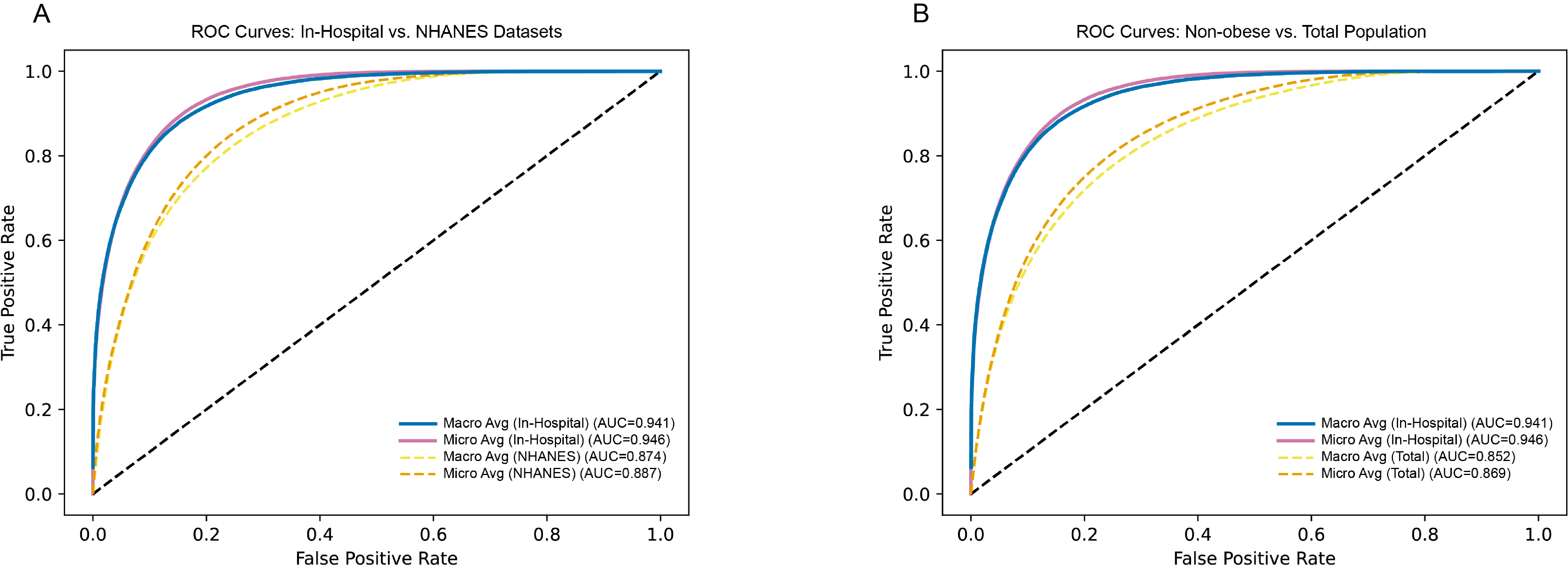


(A) Receiver Operating Characteristic (ROC) curves for the XGBoost model applied to the external National Health and Nutrition Examination Survey (NHANES) validation cohort.
(B) ROC comparison between the non-obese-specific model and a general-population model on the same non-obese test set.
