# Supplementary material for: Hepatic Steatosis Severity Prediction in Nonobese Individuals: Machine Learning Model Development and Validation
Source: J Med Internet Res. 2026 Jun 19;28:e82529. doi: 10.2196/82529 (PMC13282044; doi:10.2196/82529)
Supplement: Multimedia Appendix 12 [file jmir-v28-e82529-s012.docx]

Multimedia Appendix 12. Performance of the Binary XGBoost Model for Hepatic Steatosis Detection.


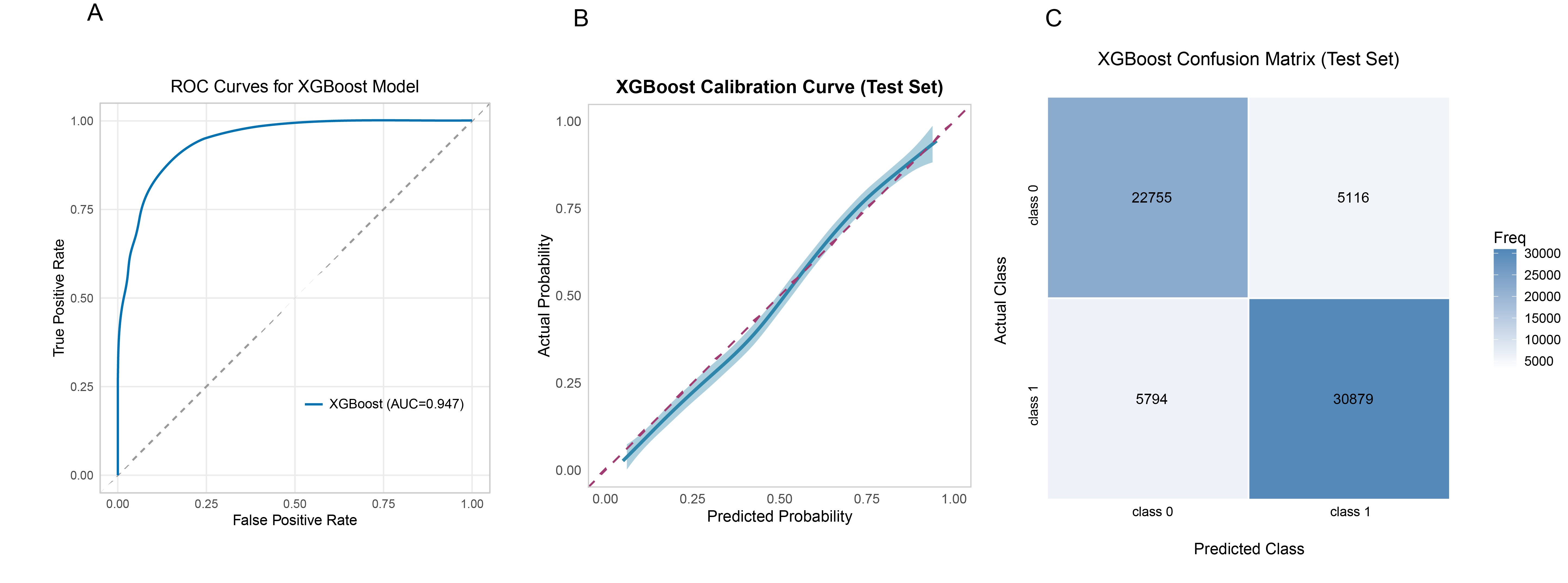


(A) Receiver Operating Characteristic (ROC) curve showing the model’s discriminative ability. (B) Calibration curve demonstrating the agreement between predicted probabilities and observed frequencies of moderate-to-severe steatosis. (C) Confusion matrix displaying the distribution of correct and incorrect classifications. XGBoost: eXtreme Gradient Boosting, CAP: Controlled Attenuation Parameter, ROC: Receiver Operating Characteristic, AUC: Area Under the Curve; Class 0: Non‑steatosis; Class 1: Mild‑to‑severe steatosis.
