# Supplementary material for: Hepatic Steatosis Severity Prediction in Nonobese Individuals: Machine Learning Model Development and Validation
Source: J Med Internet Res. 2026 Jun 19;28:e82529. doi: 10.2196/82529 (PMC13282044; doi:10.2196/82529)
Supplement: Multimedia Appendix 13 [file jmir-v28-e82529-s013.docx]

Multimedia Appendix 13. Performance Comparison Between the Binary XGBoost Model and Fatty Liver Index for Hepatic Steatosis Detection.

|  | Internal Test Set | | | | | External Validation Set | | | | |  |
| --- | --- | --- | --- | --- | --- | --- | --- | --- | --- | --- | --- |
|  | AUC^a^ | Accuracy | Precision | Recall | F1 Score | AUC | Accuracy | Precision | Recall | F1 Score |  |
| XGBoost^b^ binary model | 0.947 | 0.831 | 0.858 | 0.842 | 0.850 | 0.892 | 0.806 | 0.834 | 0.821 | 0.828 |  |
|  |  |  |  |  |  |  |  |  |  |  |  |
| FLI^c^ Score | 0.809 | 0.732 | 0.673 | 0.729 | 0.700 | 0.792 | 0.715 | 0.678 | 0.716 | 0.696 |  |

Note: This table summarizes the key performance metrics of the binary XGBoost model and FLI for detecting hepatic steatosis in the internal test set and external NHANES non-Hispanic Asian cohort. ᵃAUC: area under the curve; ᵇXGBoost: extreme gradient boosting; ᶜFLI: fatty liver index.
