# Supplementary material for: Hepatic Steatosis Severity Prediction in Nonobese Individuals: Machine Learning Model Development and Validation
Source: J Med Internet Res. 2026 Jun 19;28:e82529. doi: 10.2196/82529 (PMC13282044; doi:10.2196/82529)
Supplement: Multimedia Appendix 14 [file jmir-v28-e82529-s014.docx]

Multimedia Appendix 14. Comparison of ROC Curves Between the Binary XGBoost Model and Fatty Liver Index (FLI) for Hepatic Steatosis Detection.


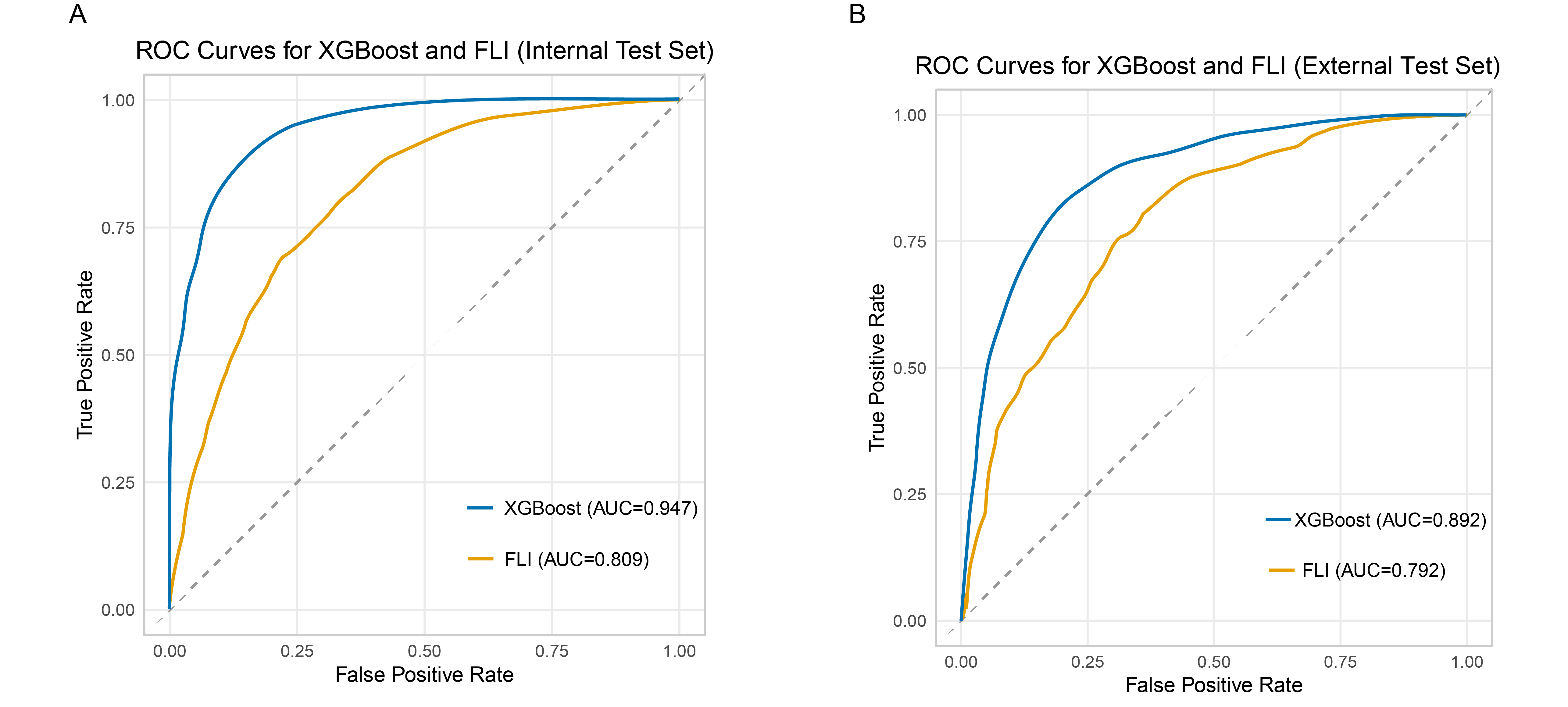


ROC curves comparing the binary XGBoost model and FLI for detecting mild steatosis and above (CAP >248 dB/m) in the internal test set (A) (XGBoost AUC=0.947, FLI AUC=0.809) and the external NHANES non-Hispanic Asian cohort (B) (XGBoost AUC=0.892, FLI AUC=0.792). ROC: Receiver Operating Characteristic; AUC: Area Under the Curve; FLI: Fatty Liver Index; CAP: Controlled Attenuation Parameter.
