# Supplementary material for: Hepatic Steatosis Severity Prediction in Nonobese Individuals: Machine Learning Model Development and Validation
Source: J Med Internet Res. 2026 Jun 19;28:e82529. doi: 10.2196/82529 (PMC13282044; doi:10.2196/82529)
Supplement: Multimedia Appendix 15 [file jmir-v28-e82529-s015.docx]

Multimedia Appendix 15. Performance of the 9-core-feature XGBoost Model on the Test Set.


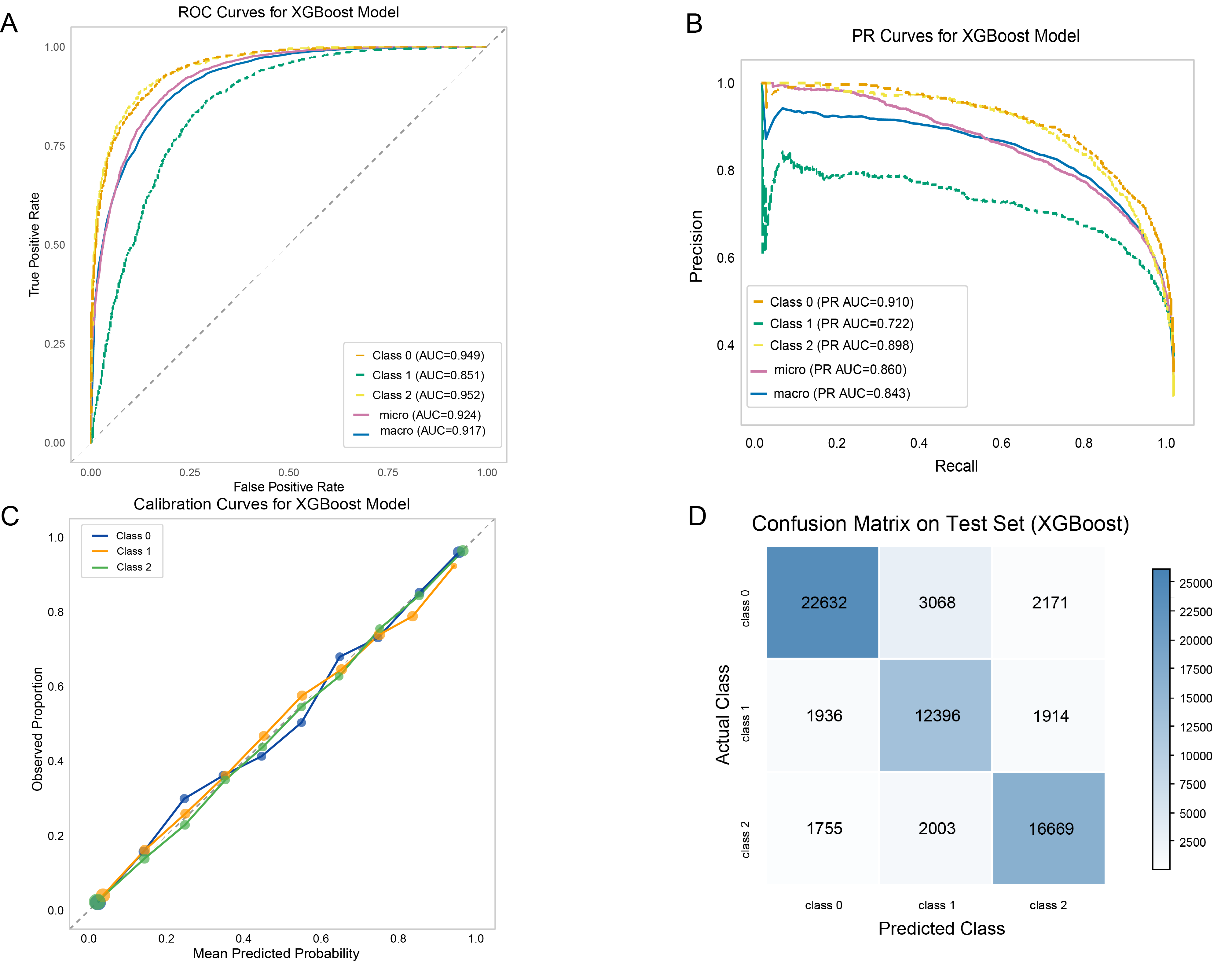


(A) Multi-class ROC curves of the 9-core-feature model, with AUC values for each steatosis grade and average performance. (B) PR curves of the 9-core-feature model, showing precision-recall trade-off and PR-AUC values. (C) Calibration plots for the XGBoost algorithms on the test set. (D) Heatmap of correct/incorrect classifications across steatosis grades (0=None, 1=Mild, 2=Moderate-to-Severe). ROC: Receiver Operating Characteristic; AUC: Area Under the Curve; PR: Precision‑Recall; XGBoost: eXtreme Gradient Boosting; Class 0: Non‑steatosis; Class 1: Mild steatosis; Class 2: Moderate‑to‑severe steatosis.
