# Supplementary material for: Hepatic Steatosis Severity Prediction in Nonobese Individuals: Machine Learning Model Development and Validation
Source: J Med Internet Res. 2026 Jun 19;28:e82529. doi: 10.2196/82529 (PMC13282044; doi:10.2196/82529)
Supplement: Multimedia Appendix 16 [file jmir-v28-e82529-s016.docx]

Multimedia Appendix 16. Performance Comparison Between 14-feature and 9-core-feature XGBoost Models for Non-obese Hepatic Steatosis Severity Prediction.

|  | 14-feature Model | 9-core-feature Model |
| --- | --- | --- |
| Accuracy | 0.824 | 0.801 |
| Cohen's Kappa | 0.713 | 0.708 |
| macro average ROC-AUC^a^ | 0.941 | 0.917 |
| micro average ROC-AUC | 0.946 | 0.924 |
| macro average PR-AUC^b^ | 0.890 | 0.843 |
| micro average PR-AUC | 0.899 | 0.860 |
| Precision (None) | 0.879 | 0.860 |
| Recall(None) | 0.840 | 0.812 |
| Specificity (None) | 0.912 | 0.899 |
| F1 Score (None) | 0.859 | 0.835 |
| ROC-AUC (None) | 0.965 | 0.949 |
| Precision (Mild) | 0.731 | 0.710 |
| Recall (Mild) | 0.783 | 0.763 |
| Specificity (Mild) | 0.903 | 0.895 |
| F1 Score (Mild) | 0.756 | 0.735 |
| ROC-AUC (Mild) | 0.890 | 0.851 |
| Precision (Moderate to Severe) | 0.832 | 0.803 |
| Recall (Moderate to Severe) | 0.836 | 0.816 |
| Specificity (Moderate to Severe) | 0.922 | 0.907 |
| F1 Score (Moderate to Severe) | 0.834 | 0.810 |
| ROC-AUC (Moderate to Severe) | 0.969 | 0.952 |

Note: Key performance metrics of two models (14-feature vs. 9-core-feature) on the test set.: ^a^ROC-AUC: Area Under the Receiver Operating Characteristic Curve, ^b^PR-AUC: Area Under the Precision-Recall Curve.
